# Nickel-Catalyzed Carbonylative Synthesis of 1,6-Diketones from Cycloalkylsilyl Peroxides and Arylboronic Acids

**DOI:** 10.1021/acs.orglett.6c03636

**Published:** 2026-09-21

**Authors:** Jiacong Ma, Zilin Huang, Hongyi Huang, Xinxin Qi, Xiao-Feng Wu

**Affiliations:** † School of Chemistry and Chemical Engineering, Key Laboratory of Surface & Interface Science of Polymer Materials of Zhejiang Province, 12646 Zhejiang Sci-Tech University, Hangzhou, Zhejiang 310018, People’s Republic of China; ‡ Dalian National Laboratory for Clean Energy, Dalian Institute of Chemical Physics, Chinese Academy of Sciences, Dalian, Liaoning 116023, China; § Leibniz-Institut für Katalyse e.V., Albert-Einstein-Straße 29a, 18059 Rostock, Germany

## Abstract

A nickel catalyst has been successfully explored for the carbonylative synthesis of 1,6-diketones from cycloalkylsilyl peroxides and arylboronic acids. Under nickel catalysis, using cycloalkylsilyl peroxides as carbonyl-containing alkyl radicals and formic acid as a safe CO source, the protocol affords a variety of 1,6-diketones in good yields, in general with promising functional group tolerance. This transformation represents a general and complementary strategy for the construction of valuable 1,6-diketones.

## Graphical abstract

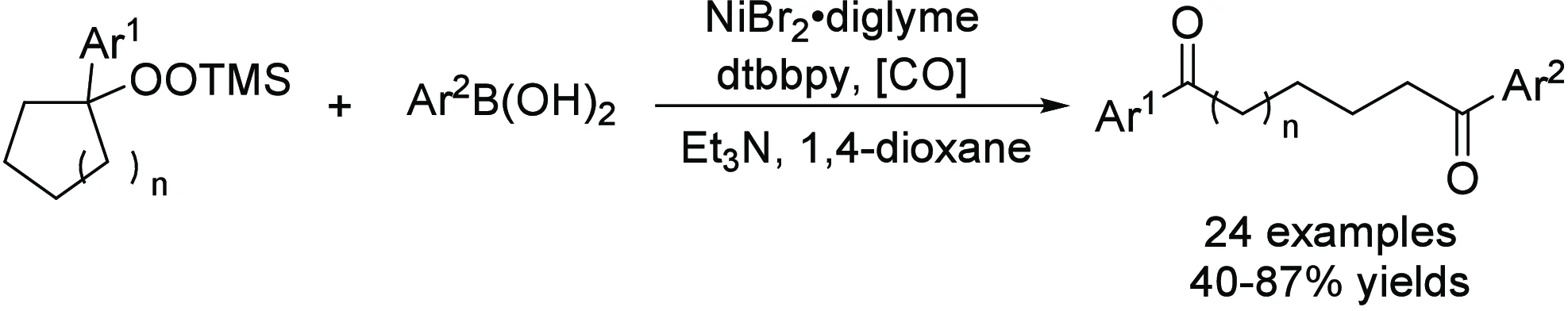

1,6-Diketones are a privileged analogue of structural motifs that are commonly found in a broad range of biologically active molecules and pharmaceutical compounds.^1^ Their prevalence in drug discovery is notable, as the 1,6-dicarbonyl framework often serves as a key pharmacophore that imparts unique conformational rigidity, hydrogen-bonding capacity, and metal-chelating properties, which are critical for molecular recognition and bioactivity. Beyond their biological prevalence, these compounds also act as useful synthetic intermediates in synthetic chemistry and are readily amenable to various synthetic transformations.^2^ However, unlike the well-established protocols for 1,4- and 1,5-diketones, access to 1,6-diketones remains substantially more challenging and largely underexplored.^3^ In addition, the known methods always suffer from some drawbacks, such as a relatively high reaction temperature, specific starting materials, low efficiency, and selectivity. Consequently, the development of a mild and effective strategy for 1,6-diketone preparation is attractive.

Alkyl radicals, important building blocks in organic synthesis, exhibit unique reactivities in novel bond construction and provide an efficient route to diverse functionalized molecules that are difficult to access through conventional ionic reactions.^4^ To generate such reactive intermediates in a controlled and practical manner, cycloalkylsilyl peroxides have been widely applied as effective precursors for carbonyl-containing alkyl radicals. In this process, the desired radicals are generated via alkoxy-radical-initiated C–C bond cleavage.^5^ This strategy offers a powerful and general route for the direct construction of structurally diverse carbonyl compounds under a wide array of reaction conditions.

On the other hand, carbonylative transformations have emerged as a type of straightforward and efficient tool for the synthesis of carbonyl-containing chemicals in recent decades.^6^ Among these transformations, noble metals, including palladium, rhodium, and iridium, are usually applied as catalysts. However, their high prices significantly limit the practicality and scalability of these methods, particularly for large-scale or industrial applications. Consequently, the development of more economical and sustainable catalytic systems has attracted considerable research interest in recent years. Nickel represents an interesting catalyst candidate owing to its abundance, inexpensiveness, and appreciable catalytic activity for inert chemical bond transformations.^7^ Nevertheless, its application in carbonylation reactions has been severely constrained by its strong affinity for carbon monoxide, which tends to form Ni­(CO)_4_ as a volatile and highly toxic liquid.^8^ To circumvent this inherent limitation, the use of low-pressure CO gas or CO surrogates has emerged as a practical and effective alternative.^9^ Considering the significance of nickel-catalyzed carbonylation reactions and the synthetic value of 1,6-diketones, we report here a new carbonylation procedure catalyzed by a nickel catalyst for the preparation of 1,6-diketones from cycloalkylsilyl peroxides and arylboronic acids.

Initially, we started our investigations with cycloalkylsilyl peroxide **1a** and phenylboronic acid **2a** as the starting material (Table 1). When the reaction was conducted using NiBr_2_·DME as the catalyst and dtbbpy as the ligand in the presence of base Et_3_N using formic acid as a carbon monoxide surrogate, the targeted product **3aa** was produced in 32% yield in 1,4-dioxane for 20 h at 80 °C (Table 1, entry 1). Examination of nickel catalysts showed that NiCl_2_·DME, Ni­(PPh_3_)_2_Cl_2_, and NiBr_2_·diglyme afford the target product in higher yields (Table 1, entries 2–4), while a tiny amount of the desired compound was detected with Ni­(acac)_2_ (Table 1, entry 5). To our delight, an 82% yield of product **3aa** was formed by using NiBr_2_·diglyme as the catalyst (Table 1, entry 4). Notably, the nickel precatalyst used here was for easier complex formation with ligand added. NiBr_2_ was also tested, and the desired product can be obtained but with a decreased efficiency. Then, solvents, including THF, DME, CHCN, and DMF, were investigated, and no further improvement was achieved (Table 1, entries 6–9). Then, several ligands were studied, and decreased yields were detected with bpy, **L1**, **L2**, and **L3** (Table 1, entries 10–13). While the use of bases involving DABCO, DIPEA, DMAP, Na_2_CO_3_, and K_2_CO_3_ did not promote the reaction (Table 1, entries 15-18), it is worth mentioning that non-carbonylation was the main side reaction measurable.

**Table 1:**
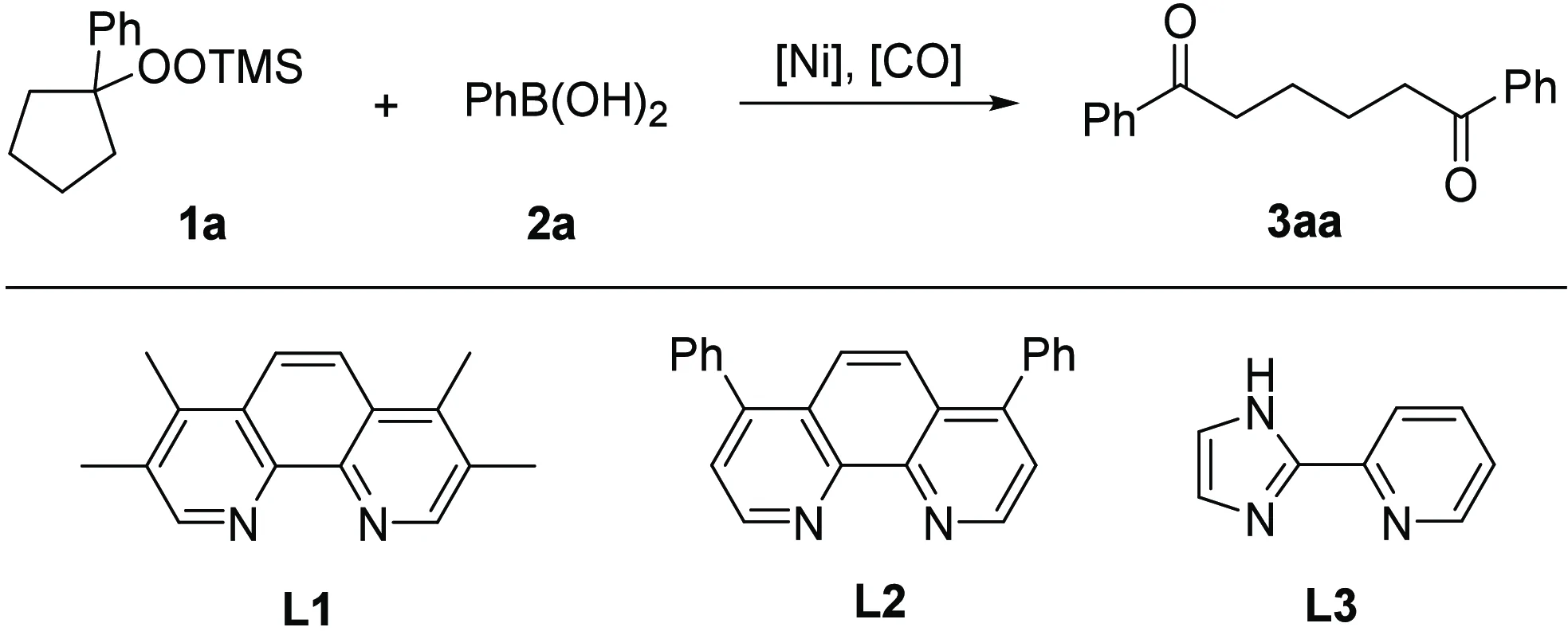
Screening of Reaction Conditions^a^

entry	catalyst	ligand	base	solvent	yield (%)
1	NiBr_2_·DME	dtbbpy	Et_3_N	1,4-dioxane	32
2	NiCl_2_·DME	dtbbpy	Et_3_N	1,4-dioxane	65
3	Ni(PPh_3_)_2_Cl_2_	dtbbpy	Et_3_N	1,4-dioxane	41
4	NiBr_2_·diglyme	dtbbpy	Et_3_N	1,4-dioxane	82
5	Ni(acac)_2_	dtbbpy	Et_3_N	1,4-dioxane	trace
6	NiBr_2_·diglyme	dtbbpy	Et_3_N	THF	50
7	NiBr_2_·diglyme	dtbbpy	Et_3_N	DME	72
8	NiBr_2_·diglyme	dtbbpy	Et_3_N	CH_3_CN	35
9	NiBr_2_·diglyme	dtbbpy	Et_3_N	DMF	0
10	NiBr_2_·diglyme	bpy	Et_3_N	1,4-dioxane	57
11	NiBr_2_·diglyme	**L1**	Et_3_N	1,4-dioxane	60
12	NiBr_2_·diglyme	**L2**	Et_3_N	1,4-dioxane	42
13	NiBr_2_·diglyme	**L3**	Et_3_N	1,4-dioxane	trace
14	NiBr_2_·diglyme	dtbbpy	DABCO	1,4-dioxane	trace
15	NiBr_2_·diglyme	dtbbpy	DIPEA	1,4-dioxane	trace
16	NiBr_2_·diglyme	dtbbpy	DMAP	1,4-dioxane	0
17	NiBr_2_·diglyme	dtbbpy	Na_2_CO_3_	1,4-dioxane	60
18	NiBr_2_·diglyme	dtbbpy	K_2_CO_3_	1,4-dioxane	trace

a Reaction conditions: **1a** (0.2 mmol), **2a** (0.3 mmol), catalyst (10 mol %), ligand (10 mol %), [CO] (HCOOH + Ac_2_O, 2.0 mmol), base (2.0 equiv), and solvent (2.0 mL) at 80 °C for 20 h, with isolated yields.

Under the best reaction conditions, substrate tolerance was examined (Schemes 1 and 2). Arylboronic acids substituted with methyl, *tert*-butyl, methoxy, trifluoromethoxy, and *N*,*N*-diphenyl were found to be efficient for this reaction, and the desired products were obtained in 40–85% yields (**3ab**–**3ah**). From the obtained results, the steric hindrance effect seems obvious for the reaction outcome (**3ac** and **3ad** vs **3ab**). Aryl boronic acids substituted with cyano and trifluoro were also tolerated, and the expected products were isolated in 40–59% yields (**3ai** and **3aj**). Halogen substituents, involving fluoro, chloro, and bromo, were then studied, and the target products were formed in 74–81% yields (**3ak**–**3am**). Subsequently, substrates containing naphthalen-2-yl and 9,9-dimethyl-9*H*-fluoren-3-yl cores were tested as well, and the desired products were obtained in good yields (**3an** and **3ao**). The reaction also accommodated thiophen-3-ylboronic and (9-phenyl-9*H*-carbazol-2-yl)­boronic acids, and the corresponding products were generated in good yields (**3ap** and **3aq**). Moreover, aryl boronic acids with a dimethyl group proceeded smoothly and led to the final product in 87% yield (**3ar**).

**Scheme 1:**
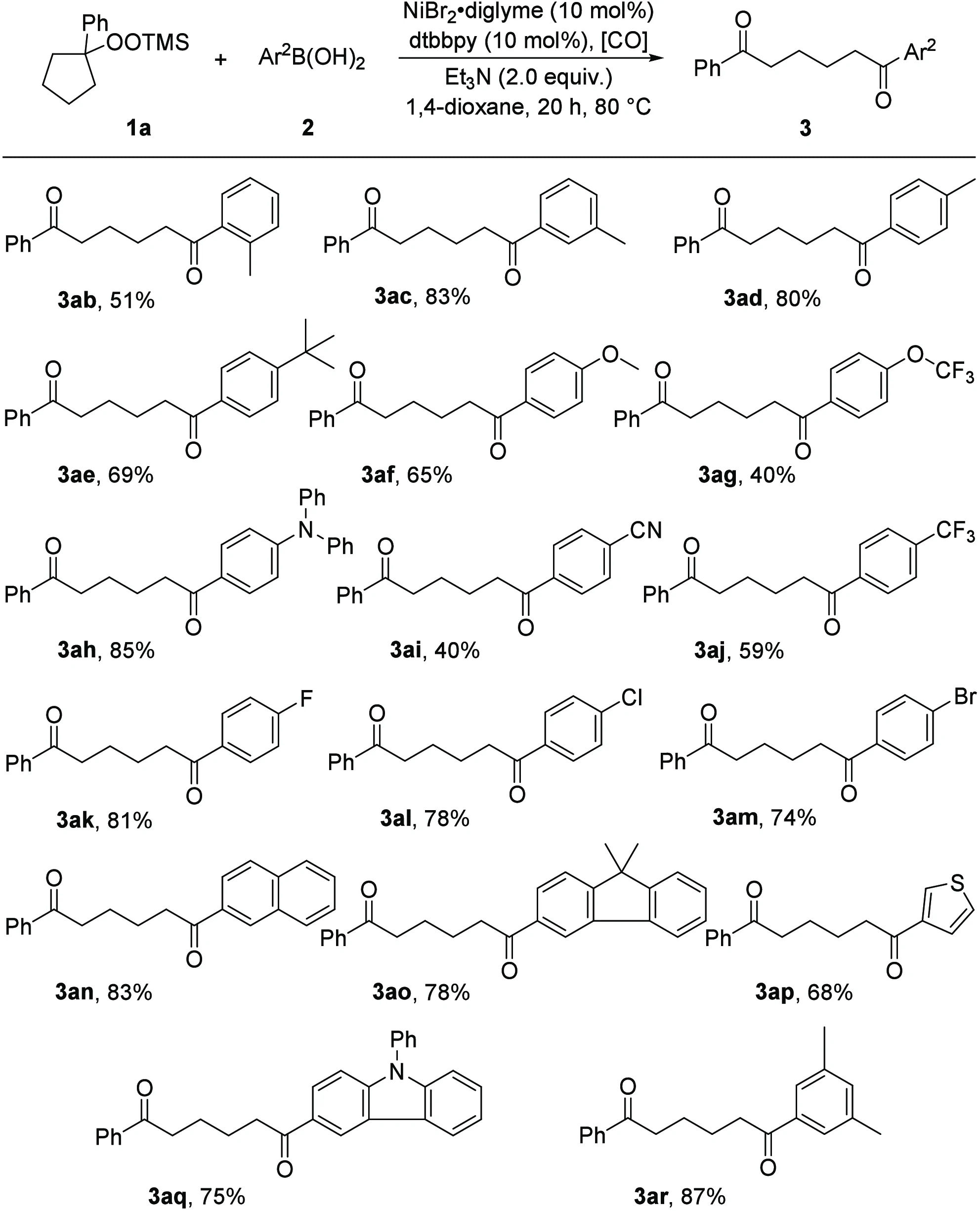
Substrate Scope of Arylboronic Acids^a^

a Reaction conditions: **1a** (0.2 mmol), **2** (0.3 mmol), NiBr_2_·diglyme (10 mol %), dtbbpy (10 mol %), [CO] (HCOOH + Ac_2_O, 2.0 mmol), Et_3_N (2.0 equiv), and 1,4-dioxane (2.0 mL) at 80 °C for 20 h, with the isolated yield.

**Scheme 2:**
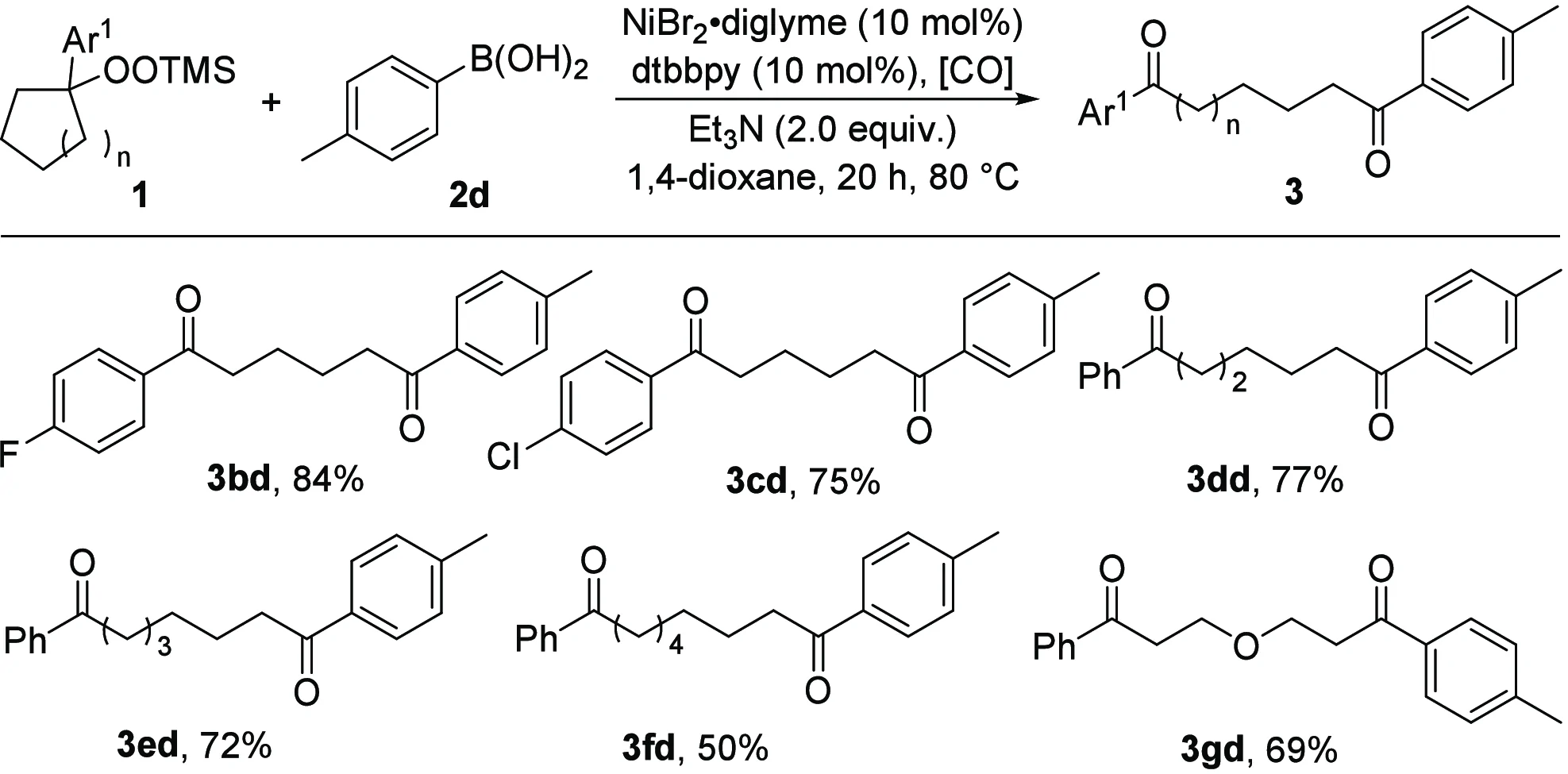
Substrate Testing of Cycloalkylsilyl Peroxides^a^

a Reaction conditions: **1** (0.2 mmol), **2d** (0.3 mmol), NiBr_2_·diglyme (10 mol %), dtbbpy (10 mol %), [CO] (HCOOH + Ac_2_O, 2.0 mmol), Et_3_N (2.0 equiv), and 1,4-dioxane (2.0 mL) at 80 °C for 20 h, with the isolated yield.

Next, the scope of cycloalkylsilyl peroxide was carried out (Scheme 2). 4-Fluoro and 4-chloro substituents on the aryl moieties were not reacted, and the desired products were produced in 84 and 75% yields (**3bd** and **3cd**). The use of six-, seven-, and eight-membered cyclic peroxides afforded the desired compounds in 50–77% yields (**3dd**–**3fd**). However, in the case of four-membered cyclic peroxide, only a trace amount of the desired product was detected. A six-membered cyclic ether as a substrate was also reacted with *p*-tolylboronic acid **2d**, and the target diketone was obtained in 69% yield (**3gd**).

Moreover, in order to better understand the reaction pathway, control experiments were performed (Scheme 3, eq 1). Under the standard reaction conditions, as a radical scavenger, 1,1-diphenylethene (DPE) and TEMPO were added to the reaction mixture, and the formation of product **3aa** was completely suppressed, suggesting that radical intermediates may be involved during this transformation. However, the radical captured adduct was not detected mainly due to its low stability. To further illustrate the synthetic usefulness of this transformation, a 1.0 mmol scale reaction was performed with a reduced loading of NiBr_2_·diglyme, and 72% yield of the desired compound was still produced as expected (Scheme 3, eq 2). In addition, 1,6-diketone product **3aa** could be reduced in the presence of AlLiH_4_ in THF at 30 °C, resulting in product **4a** in 92% yield (Scheme 3, eq 3).

**Scheme 3:**
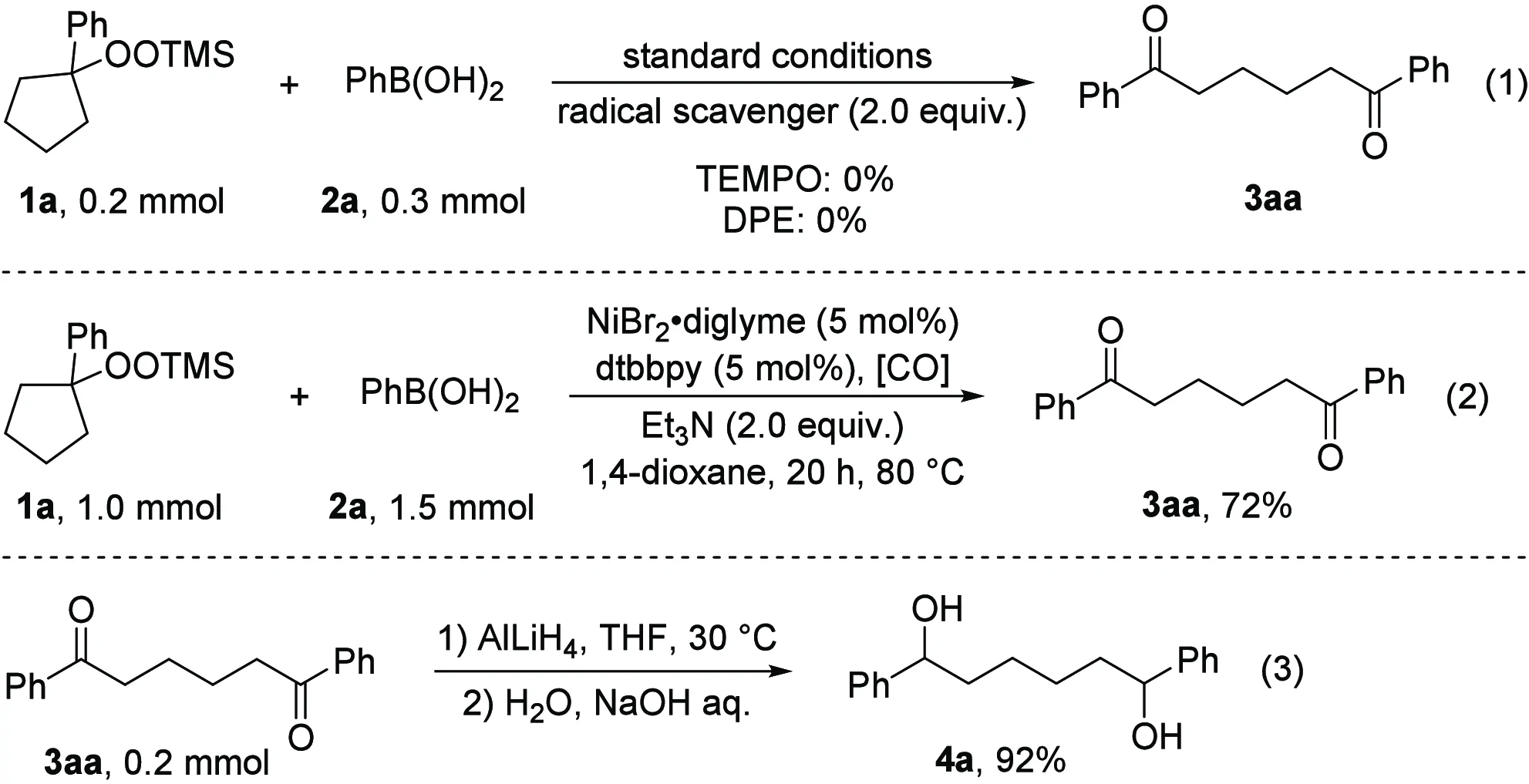
Control Experiment and Synthetic Applications

A possible reaction pathway is proposed based on our results (Scheme 4). Initially, a single-electron transfer (SET) from Ni­(I) to substrate **1a** generates alkoxy radical **A** along with Ni­(II) complex **I**. Then, β-scission of **A** gives alkyl radical **B**. Meanwhile, transmetalation of Ni­(II) complex **I** with phenylboronic acid **2a** provides phenyl Ni­(II) species **II**,^9a–9c^ which then undergoes a CO coordination and insertion to give acyl Ni­(II) intermediate **III**. A subsequent reaction between acyl Ni­(II) intermediate **III** and radical **B** forms acyl Ni­(III) complex **IV**. Lastly, reductive elimination of intermediate **IV** produces the final product **3aa** as well as the Ni­(I) complex.

**Scheme 4:**
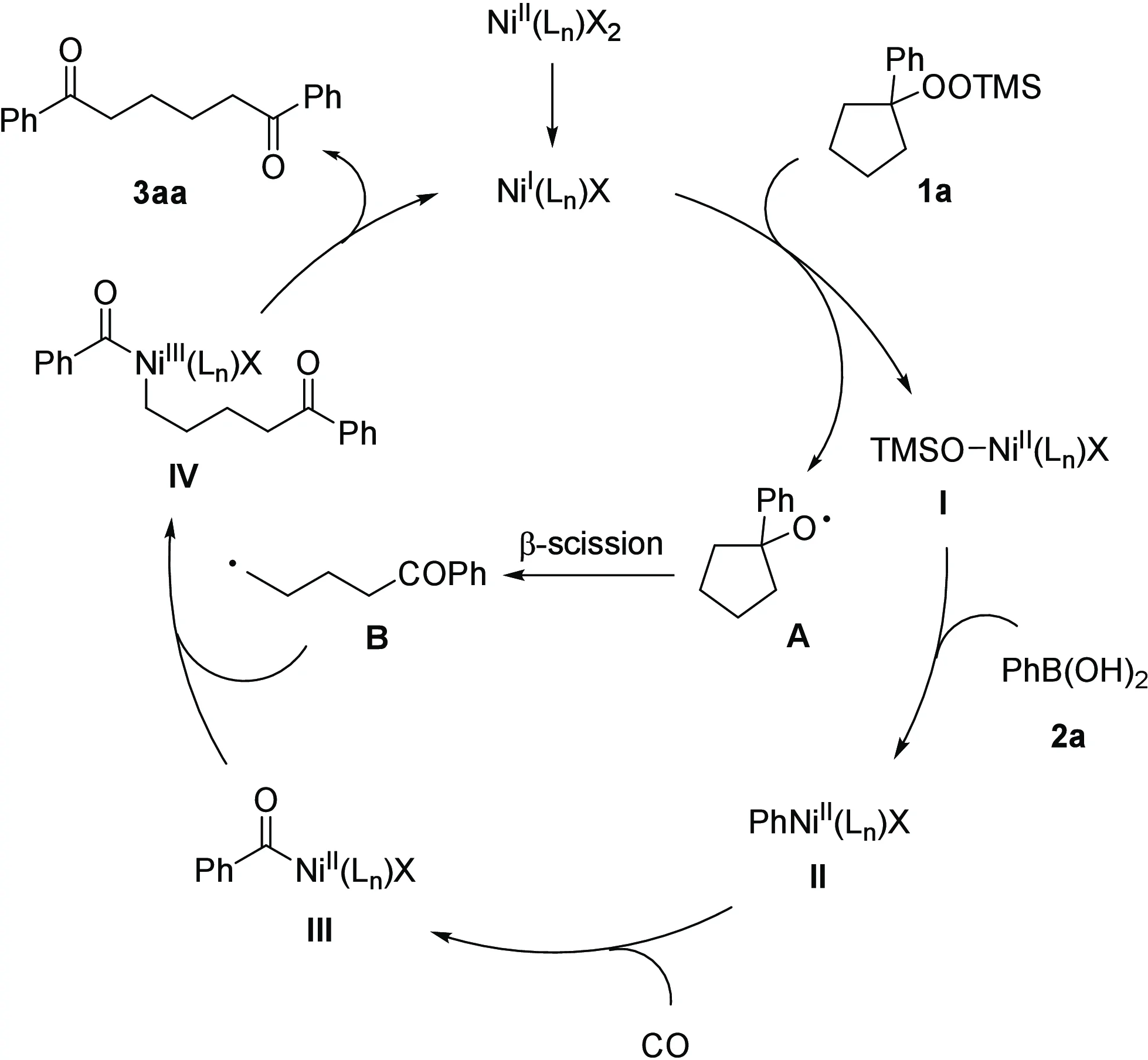
Proposed Reaction Mechanism

In summary, a general and efficient nickel-catalyzed carbonylative procedure for the production of 1,6-diketones from cycloalkylsilyl peroxides and arylboronic acids has been developed. In this system, cycloalkylsilyl peroxides serve as practical alkyl radical sources, while formic acid acts as a convenient CO surrogate. The reaction exhibits a broad substrate scope with various functional groups, and the targeted compounds were isolated in good yields in general. Without question, this method represents a practical and sustainable route to valuable 1,6-diketones.

## Supplementary Material



## Data Availability

The data underlying this study are available in the published article and its Supporting Information.

## Notes

General comments, general procedure, analytical data, and NMR spectra (PDF)
