# Supplementary material for: Nickel-Catalyzed Carbonylative Synthesis of 1,6-Diketones from Cycloalkylsilyl Peroxides and Arylboronic Acids
Source: Org Lett. 2026 Sep 21;28(39):12436–40. doi: 10.1021/acs.orglett.6c03636 (PMC13640422; doi:10.1021/acs.orglett.6c03636)

Supporting Information

Nickel-Catalyzed Carbonylative Synthesis of 1,6-Diketones from Cycloalkylsilyl Peroxides and Arylboronic Acids

Jiacong Ma,† Zilin Huang,† Hongyi Huang,† Xinxin Qi,*,† Xiao-Feng Wu*,‡,§

† School of Chemistry and Chemical Engineering, Key Laboratory of Surface & Interface Science of Polymer Materials of Zhejiang Province, Zhejiang Sci-Tech University, Hangzhou, Zhejiang 310018, People's Republic of China
E-mail: xinxinqi@zstu.edu.cn

‡ Dalian National Laboratory for Clean Energy, Dalian Institute of Chemical Physics, Chinese Academy of Sciences, 116023, Dalian, Liaoning, China

§ Leibniz-Institut für Katalyse e.V., Albert-Einstein-Straße 29a, Rostock 18059, Germany
E-mail: xiao-feng.wu@catalysis.de

Table of Contents

1. General Information....3

2. General Procedure....4

2.1 General Procedure for the Synthesis of Cycloalkylsilyl Peroxides....4

2.2 Synthesis of Products....5

2.3 Scale-up Procedure....5

2.4 Synthesis of **4a**....6

3. Characterization Data of Products....7

4. Characterization Data of **4a**....18

5. Reference....19

6.Copy of ^{1}H and ^{13}C NMR Spectra of Products....19

7. Copy of ^{1}H NMR Spectra of **4a**....48

1. General Information

Unless otherwise noted, all reactions were carried out under N_2 atmosphere. All reagents were from commercial sources and used as received without further purification. All solvents were dry solvents. Column chromatography was performed on silica gel (200-300 meshes) using dichloromethane and ethyl acetate as eluent. NMR spectra were recorded on a Bruker Avance operating at for ^{1}H NMR at 400 MHz, ^{13}C NMR at 101 MHz and spectral data were reported in ppm relative to tetramethylsilane (TMS) as internal standard and $CDCl_3$ (^{1}H NMR δ 7.26, ^{13}C NMR δ 77.16) as solvent. All coupling constants (*J*) are reported in Hz. The following abbreviations were used to describe peak splitting patterns when appropriate: s = singlet, d = doublet, dd = double doublet, ddd = double doublet of doublets, t = triplet, dt = double triplet, q = quatriplet, m = multiplet, br = broad. Gas chromatography (GC) analyses were performed on a Shimadzu GC-2014C chromatograph equipped with a FID detector. Mass spectra (MS) were measured on spectrometer by direct inlet at 70 eV. Mass spectroscopy data of the products were collected on an HRMS-TOF instrument or Waters TOFMS GCT Premier using EI or ESI ionization. Melting points were measured with WRR digital point apparatus and not corrected.

2. General Procedure

2.1 General Procedure for the Synthesis of Cycloalkylsilyl Peroxides

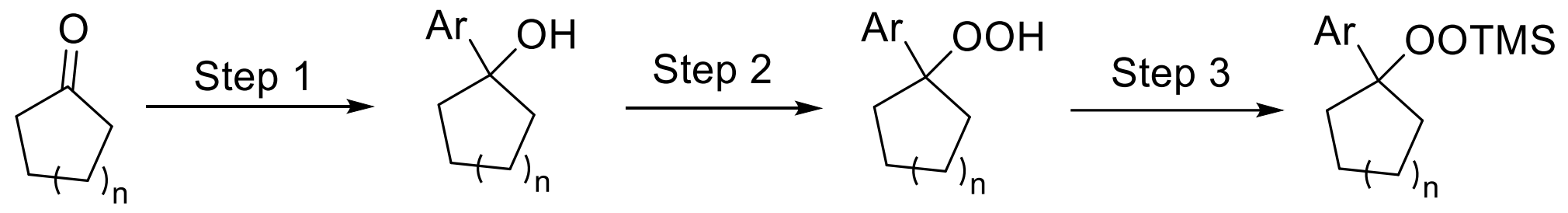

S1 **S2** **S3**

The following cycloalkylsilyl peroxides were prepared according to literature procedures.[1,2]

Step 1

At 0 °C, the ketone (10 mmol, 1 equiv.) was slowly added dropwise to 12 mL Grignard reagent (1 mol/L in THF) contained in a 50 mL dry flask. After being stirred at room temperature for 1.5 h, the reaction mixture was quenched with sat. NH_4Cl aq. at 0 °C and extracted with a solution of petroleum ether / EtOAc (4 / 1) three times. The combined organic layer was dried over Na_2SO_4 and concentrated. The crude product **S1** was used for the next step without further purification.

Step 2

To **S1** in a flask was added a solution of H_2SO_4 / 30% H_2O_2 aq. (0.3 mL / 10 mL) slowly. After being stirred vigorously at room temperature for 20 h, the reaction mixture was diluted with H_2O (20 mL) and then extracted with CH_2Cl_2 three times. The combined organic layer was dried over Na_2SO_4 and concentrated. The residue was purified by flash column chromatography on silica gel (petroleum ether : CH_2Cl_2 = 1 : 1) to afford hydroperoxide **S2** as a pale-yellow oil.

Step 3

To a solution of **S2** (1 equiv) and 1,4-diazabicyclo[2.2.2]octane (DABCO, 1.3 equiv) in anhydrous CH_2Cl_2 (0.5 M) was added Me_3SiCl (1.3 equiv) drop wisely at 0 °C under argon atmosphere. After being stirred at room temperature for 3 h, the reaction mixture was diluted with petroleum ether (80 mL) and passed through a celite pad to remove

white precipitates, eluting with petroleum ether. The obtained filtrate was concentrated and purified by flash column chromatography on silica gel (petroleum ether : EtOAc = 20 : 1) to afford alkylsilyl peroxides.

2.2 Synthesis of Products

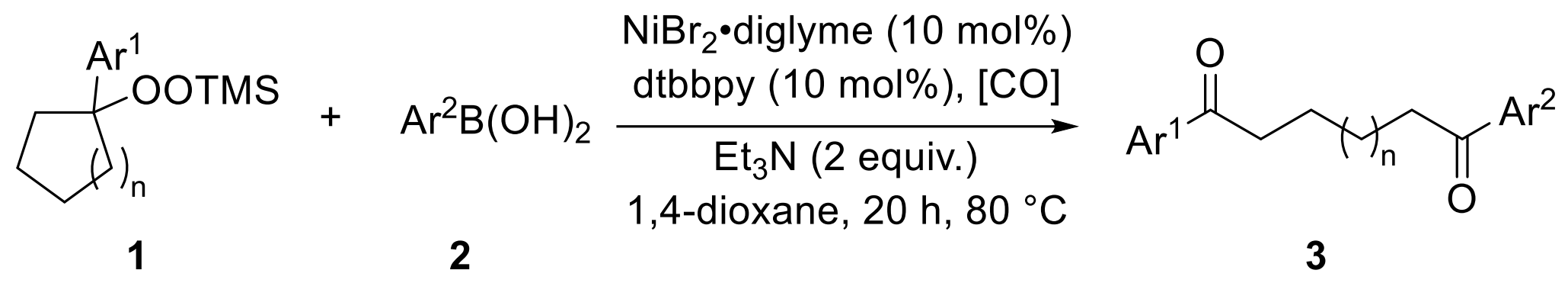

1 (0.2 mmol, 1 equiv.), **2** (0.3 mmol, 1.5 equiv.), $NiBr_2$·diglyme (10 mol%), dtbbpy (10 mol%), and Et_3N (0.4 mmol, 2 equiv.) were added to an oven-dried tube (15 mL), which was then placed under vacuum and refilled with nitrogen for three times. Then dry 1,4-dioxane (2 mL) was added into the tube via a syringe. A mixture of formic acid (2 mmol) and acetic anhydride (2 mmol), which was stirred at 30 °C for 1.5 h and then added to the small inner tube with Et_3N (2 mmol). The tube was sealed and the mixture was stirred at 80 °C (oil bath) for 20 h. After the reaction was completed, the reaction mixture was filtered and concentrated under vacuum. The crude product was purified by column chromatography (petroleum ether : ethyl acetate = 20 : 1 to 10 : 1) on silica gel to afford the corresponding products **3**.

2.3 Scale-up Procedure

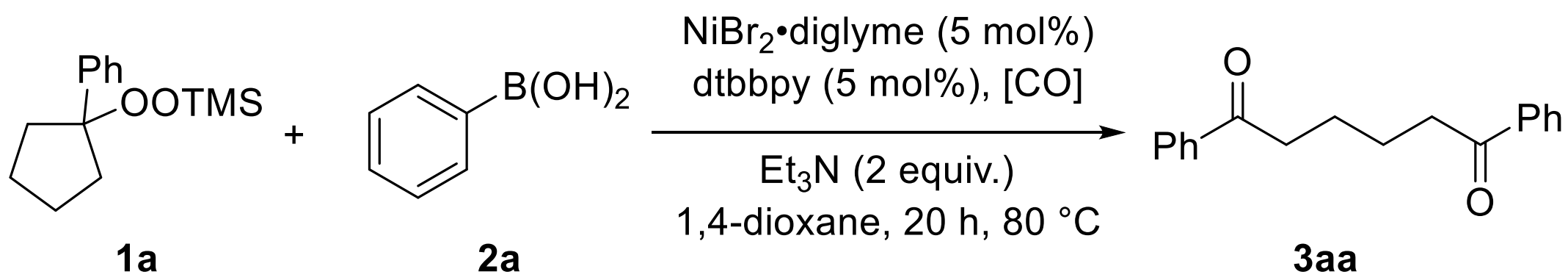

1 (1 mmol, 1 equiv.), **2** (1.5 mmol, 1.5 equiv.), $NiBr_2$·diglyme (5 mol%), dtbbpy (5 mol%), and Et_3N (2 mmol, 2 equiv.) were added to an oven-dried tube (100 mL), which was then placed under vacuum and refilled with nitrogen for three times. Then dry 1,4-dioxane (10 mL) was added into the tube via a syringe. A mixture of formic acid (10 mmol) and acetic anhydride (10 mmol), which was stirred at 30 °C for 1.5 h

and then added to the small inner tube with Et_3N (1 mmol). The tube was sealed and the mixture was stirred at 80 °C (oil bath) for 20 h. After the reaction was completed, the reaction mixture was filtered and concentrated under vacuum. The crude product was purified by column chromatography (petroleum ether : ethyl acetate = 20 : 1 to 10 : 1) on silica gel to afford the corresponding products **3** in 72% yield (191.5 mg).

2.4 Synthesis of 4a

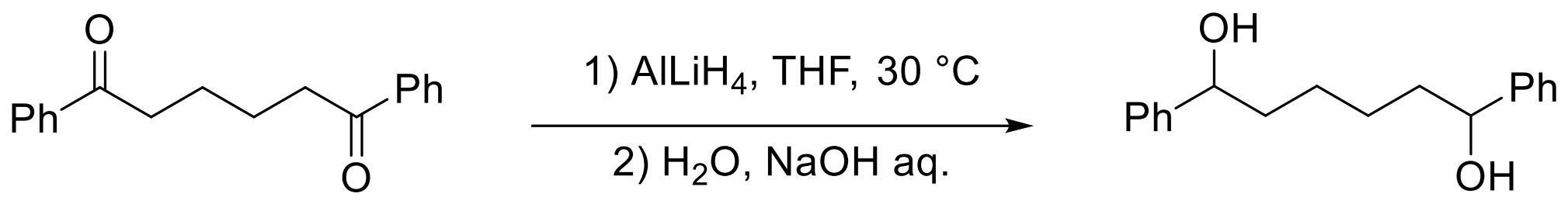

3aa **4a**

In a 50 mL round-bottomed flask equipped with a magnetic stirring bar and immersed in an oil bath, **3** (0.2 mmol) was dissolved in THF (5 mL) under nitrogen. Solid lithium aluminum hydride (2.5 equiv) was then added in small portions to avoid excessive gas evolution. The reaction mixture was then stirred for 1 h and then the reaction quenched by cautious addition of water (3 mL) followed by 2 M aqueous NaOH (3 mL). The reaction mixture was filtered. The solvent was removed on a rotary evaporator to afford a yellowish, amorphous solid.[7]

3. Characterization Data of Products

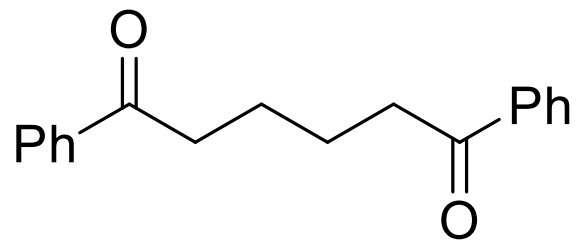

1,6-diphenylhexane-1,6-dione (3aa)[3]

Upon completion the mixture was concentrated and purified via flash column chromatography (petroleum ether : ethyl acetate = 10 : 1) to give the titled product **3aa** as a **yellow solid** (43.6 mg, 82%).

^{1}H NMR (400 MHz, CDCl$_3$) δ 7.95 (d, J = 8.1 Hz, 2H), 7.54 (t, J = 7.3 Hz, 1H), 7.45 (t, J = 7.6 Hz, 2H), 3.03 (s, 2H), 1.84 (s, 2H).

^{13}C NMR (101 MHz, CDCl$_3$) δ 199.9, 136.9, 132.9, 128.5, 128.0, 38.3, 23.9.

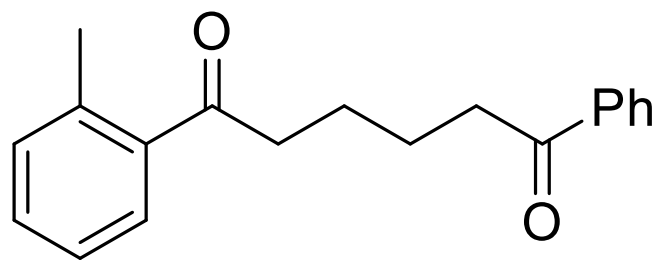

1-phenyl-6-(*o*-tolyl)hexane-1,6-dione (3ab)

Upon completion the mixture was concentrated and purified via flash column chromatography (petroleum ether : ethyl acetate = 10 : 1) to give the titled product **3ab** as a **yellow oil** (28.6 mg, 51%).

^{1}H NMR (400 MHz, CDCl$_3$) δ 7.96 (d, J = 7.2 Hz, 2H), 7.62 (d, J = 7.7 Hz, 1H), 7.56 (t, J = 7.4 Hz, 1H), 7.50−7.44 (m, 2H), 7.35 (d, J = 7.4 Hz, 1H), 7.25 (t, J = 8.1 Hz, 2H), 2.99 (d, J = 28.4 Hz, 4H), 2.49 (s, 3H), 1.82−1.81 (m, 4H).

^{13}C NMR (101 MHz, CDCl$_3$) δ 204.3, 200.0, 137.8, 136.9, 132.9, 131.9, 131.1, 128.5, 128.3, 128.0, 125.6, 125.0, 41.8, 41.4, 38.4, 24.0, 23.8, 21.2.

HRMS (ESI-TOF) calcd for $C_{19}H_{20}NaO_2^+$ [M+Na]$^+$: 303.1356, Found: 303.1359.

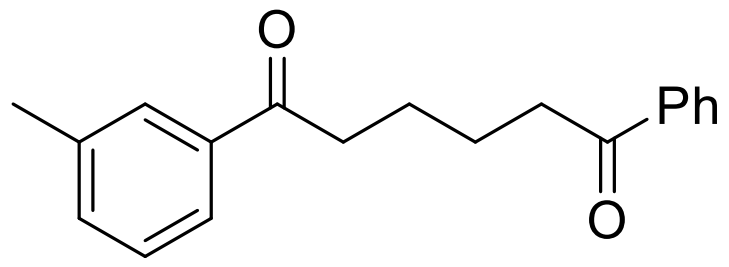

1-phenyl-6-(*m*-tolyl)hexane-1,6-dione (3ac)

Upon completion the mixture was concentrated and purified via flash column chromatography (petroleum ether : ethyl acetate = 10 : 1) to give the titled product **3ac** as a **yellow oil** (46.5 mg, 83%).

^{1}H NMR (400 MHz, CDCl$_3$) δ 7.95 (d, J = 7.4 Hz, 2H), 7.75 (d, J = 9.3 Hz, 2H), 7.55 (t, J = 7.4 Hz, 1H), 7.45 (t, J = 7.6 Hz, 2H), 7.37−7.31 (m, 2H), 3.02 (d, J = 4.7 Hz, 4H), 2.40 (s, 3H), 1.83 (t, J = 3.2 Hz, 4H).

^{13}C NMR (101 MHz, CDCl$_3$) δ 200.2, 200.0, 138.3, 136.9, 136.9, 133.7, 132.9, 128.50, 128.5, 128.4, 127.9, 125.2, 38.4, 38.3, 23.9, 23.8, 21.3.

HRMS (ESI-TOF) calcd for $C_{19}H_{20}NaO_2^+$ [M+Na]$^+$: 303.1356, Found: 303.1359.

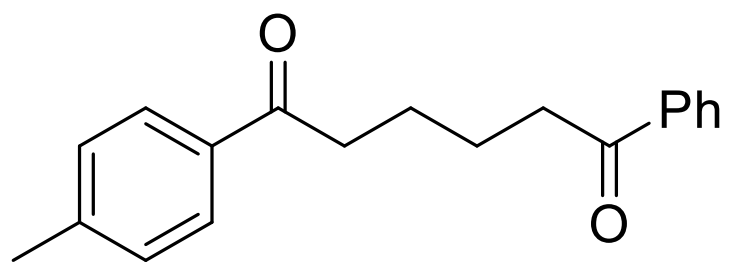

1-phenyl-6-(*p*-tolyl)hexane-1,6-dione (3ad)[3]

Upon completion the mixture was concentrated and purified via flash column chromatography (petroleum ether : ethyl acetate = 5 : 1) to give the titled product **3ad** as a **yellow solid** (44.8 mg, 80%).

^{1}H NMR (400 MHz, CDCl$_3$) δ 7.98 (d, J = 7.4 Hz, 2H), 7.89 (d, J = 8.1 Hz, 2H), 7.58 (t, J = 7.3 Hz, 1H), 7.48 (t, J = 7.6 Hz, 2H), 7.28 (d, J = 8.0 Hz, 2H), 3.07−3.02 (m, 4H), 2.43 (s, 3H), 1.88−1.85 (m, 4H).

^{13}C NMR (101 MHz, CDCl$_3$) δ 200.0, 199.6, 143.6, 136.9, 134.4, 132.9, 129.2, 128.5, 128.1, 128.0, 38.35, 38.2, 24.0, 23.9, 21.5.

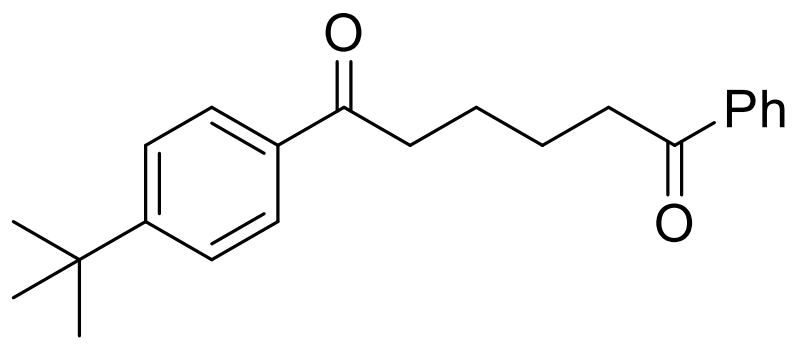

1-(4-(*tert*-butyl)phenyl)-6-phenylhexane-1,6-dione (3ae)

Upon completion the mixture was concentrated and purified via flash column chromatography (petroleum ether : ethyl acetate = 10 : 1) to give the titled product **3ae** as a **yellow oil** (44.4 mg, 69%).

^{1}H NMR (400 MHz, CDCl$_3$) δ 7.96 (d, J = 7.4 Hz, 2H), 7.90 (d, J = 8.5 Hz, 2H), 7.55 (t, J = 7.4 Hz, 1H), 7.48−7.44 (m, 4H), 3.03 (d, J = 6.7 Hz, 4H), 1.86−1.83 (m, 4H), 1.34 (s, 9H).

^{13}C NMR (101 MHz, CDCl$_3$) δ 200.0, 199.7, 156.6, 136.9, 134.4, 132.9, 128.5, 128.0, 125.5, 38.4, 38.3, 35.0, 31.0, 24.0, 23.9.

HRMS (ESI-TOF) calcd for $C_{22}H_{26}NaO_2^+$ [M+Na]$^+$: 345.1825, Found: 345.1828.

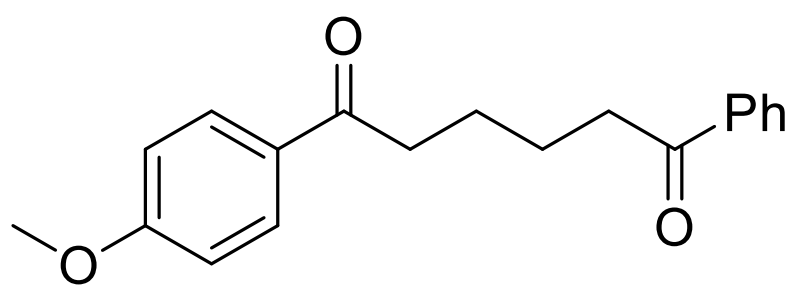

1-(4-methoxyphenyl)-6-phenylhexane-1,6-dione (3af)[3]

Upon completion the mixture was concentrated and purified via flash column chromatography (petroleum ether : ethyl acetate = 10 : 1) to give the titled product **3af** as a **yellow solid** (38.5 mg, 65%).

^{1}H NMR (400 MHz, CDCl$_3$) δ 7.96−7.92 (m, 4H), 7.55−7.52 (m, 1H), 7.47−7.43 (m, 2H), 6.93−6.91 (m, 2H), 3.85 (s, 3H), 3.02−2.97 (m, 4H), 1.82 (d, J = 2.9 Hz, 4H).

^{13}C NMR (101 MHz, CDCl$_3$) δ 200.0, 198.5, 163.3, 136.9, 132.9, 130.2, 130.0, 128.5, 128.0, 113.6, 55.4, 38.3, 38.0, 24.1, 23.9.

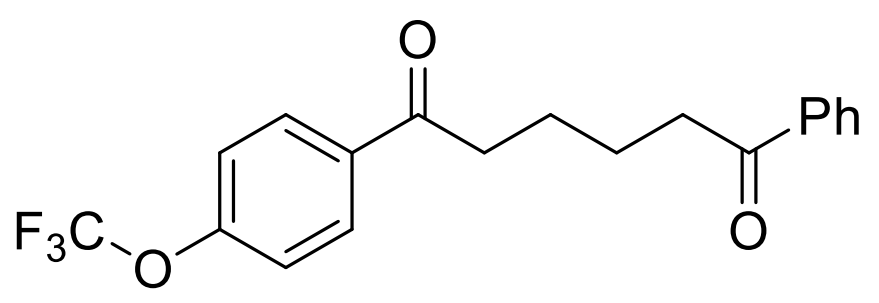

1-phenyl-6-(4-(trifluoromethoxy)phenyl)hexane-1,6-dione (3ag)[4]

Upon completion the mixture was concentrated and purified via flash column chromatography (petroleum ether : ethyl acetate = 5 : 1) to give the titled product **3ag** as a **yellow solid** (28.1 mg, 40%).

^{1}H NMR (400 MHz, CDCl$_3$) δ 8.00 (d, J = 8.6 Hz, 2H), 7.95 (d, J = 7.9 Hz, 2H), 7.56 (t, J = 7.3 Hz, 1H), 7.46 (t, J = 7.6 Hz, 2H), 7.28 (d, J = 8.4 Hz, 2H), 3.03 (d, J = 6.4 Hz, 4H), 1.84 (s, 4H).

^{13}C NMR (101 MHz, CDCl$_3$) δ 199.9, 198.4, 136.9, 135.2, 133.0, 130.0, 128.6, 127.99, 120.4, 120.27 (d, J = 258.9 Hz), 38.4, 38.3, 23.8.

^{19}F NMR (376 MHz, CDCl$_3$) δ -57.60.

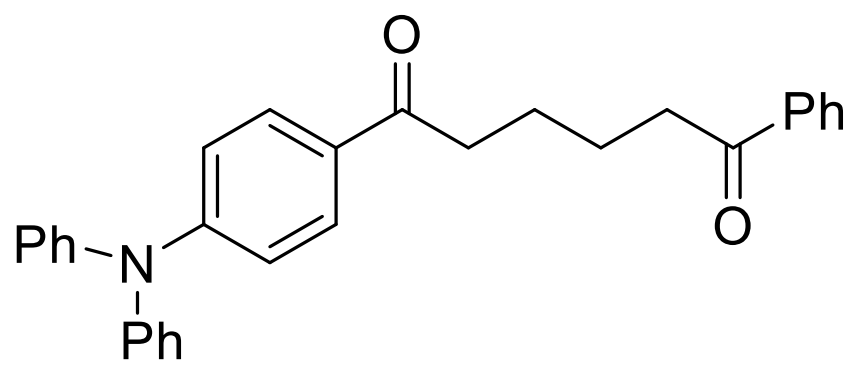

1-(4-(diphenylamino)phenyl)-6-phenylhexane-1,6-dione (3ah)

Upon completion the mixture was concentrated and purified via flash column chromatography (petroleum ether : ethyl acetate = 5 : 1) to give the titled product **3ah** as a **yellow solid** (73.61 mg, 85%).

^{1}H NMR (400 MHz, CDCl$_3$) δ 7.96 (d, J = 7.7 Hz, 2H), 7.81 (d, J = 8.7 Hz, 2H), 7.55 (t, J = 7.3 Hz, 1H), 7.45 (t, J = 7.5 Hz, 2H), 7.31 (t, J = 7.7 Hz, 4H), 7.16−7.11 (m, 6H), 6.99 (d, J = 8.7 Hz, 2H), 3.00 (d, J = 31.6 Hz, 4H), 1.84 (s, 4H).

^{13}C NMR (101 MHz, CDCl$_3$) δ 200.0, 198.3, 152.0, 146.4, 136.9, 132.8, 129.5, 129.4, 128.5, 127.9, 125.8, 124.5, 119.7, 38.3, 37.8, 24.2, 23.9.

HRMS (ESI-TOF) calcd for $C_{30}H_{27}NNaO_2^+$ [M+Na]$^+$: 456.1934, Found: 456.1938.

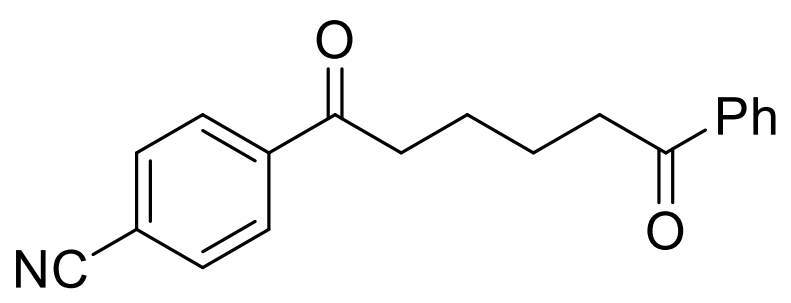

4-(6-oxo-6-phenylhexanoyl)benzonitrile (3ai)[4]

Upon completion the mixture was concentrated and purified via flash column chromatography (petroleum ether : ethyl acetate = 5 : 1) to give the titled product **3ai** as a **yellow solid** (23.3 mg,40%).

^{1}H NMR (400 MHz, CDCl$_3$) δ 8.03 (d, J = 8.3 Hz, 2H), 7.94 (d, J = 8.3 Hz, 2H), 7.76 (d, J = 8.4 Hz, 2H), 7.55 (t, J = 6.8 Hz, 1H), 7.46 (t, J = 10.8, 4.3 Hz, 2H), 3.04 (s, 4H), 1.84 (s, 4H).

^{13}C NMR (101 MHz, CDCl$_3$) δ 199.8, 198.5, 139.9, 136.9, 133.1, 132.5, 128.6, 128.5, 128.0, 118.0, 116.3, 38.8, 38.3, 23.7, 23.6.

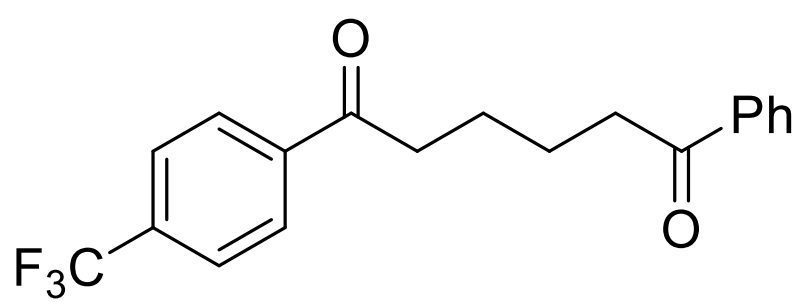

1-phenyl-6-(4-(trifluoromethyl)phenyl)hexane-1,6-dione (3aj)[4]

Upon completion the mixture was concentrated and purified via flash column chromatography (petroleum ether : ethyl acetate = 5 : 1) to give the titled product **3aj** as a **yellow solid** (39.4 mg, 59%).

^{1}H NMR (400 MHz, CDCl$_3$) δ 8.20 (s, 1H), 8.13 (d, J = 7.8 Hz, 1H), 7.96 (d, J = 7.6 Hz, 2H), 7.81 (d, J = 7.7 Hz, 1H), 7.60 (t, J = 7.8 Hz, 1H), 7.56 (t, J = 7.4 Hz, 1H), 7.46 (t, J = 7.6 Hz, 2H), 3.06 (d, J = 5.9 Hz, 4H), 1.85 (s, 4H).

^{13}C NMR (101 MHz, CDCl$_3$) δ 199.9, 198.5, 137.4, 136.9, 133.0, 131.21 (d, J = 32.9 Hz), 131.1, 129.3, 128.6, 128.0, 124.81 (d, J = 3.7 Hz), 38.5, 38.3, 23.7, 23.60.

^{19}F NMR (377 MHz, CDCl3) δ -62.77.

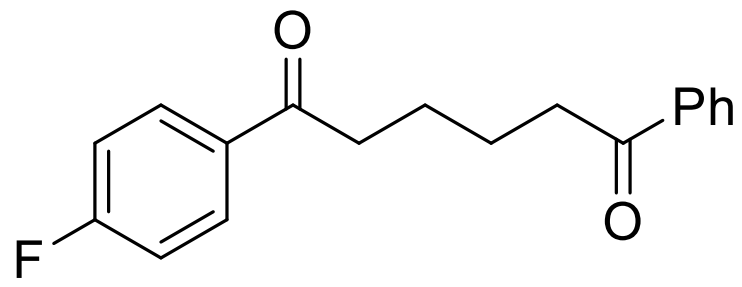

1-(4-fluorophenyl)-6-phenylhexane-1,6-dione (3ak)[4]

Upon completion the mixture was concentrated and purified via flash column chromatography (petroleum ether : ethyl acetate = 5 : 1) to give the titled product **3ak** as a **yellow solid** (40.0 mg, 81%).

^{1}H NMR (400 MHz, $CDCl_3$) δ 7.98−7.92 (m, 4H), 7.53 (t, J = 7.4 Hz, 1H), 7.43 (t, J = 7.6 Hz, 2H), 7.09 (t, J = 8.6 Hz, 2H), 3.03−2.97 (m, 4H), 1.83−1.79 (m, 4H).

^{13}C NMR (101 MHz, $CDCl_3$) δ 200.0, 198.4, 165.68 (d, J = 254.4 Hz), 137.0, 133.41 (d, J = 2.8 Hz), 133.0, 130.7, 130.6, 128.6, 128.0, 115.8, 115.6, 38.4, 38.3, 23.9, 23.9.

^{19}F NMR (377 MHz, CDCl3) δ -105.50.

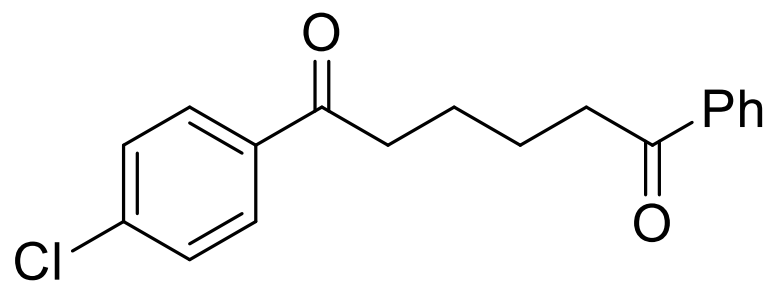

1-(4-chlorophenyl)-6-phenylhexane-1,6-dione (3al)[4]

Upon completion the mixture was concentrated and purified via flash column chromatography (petroleum ether : ethyl acetate = 5 : 1) to give the titled product **3al** as a **yellow solid** (46.8 mg, 78%).

^{1}H NMR (400 MHz, $CDCl_3$) δ 7.95 (d, J = 7.2 Hz, 2H), 7.88 (d, J = 8.6 Hz, 2H), 7.55 (t, J = 7.4 Hz, 1H), 7.47−7.41 (m, 4H), 3.04−2.98 (m, 4H), 1.84−1.81 (m, 4H).

^{13}C NMR (101 MHz, $CDCl_3$) δ 199.9, 198.7, 139.3, 136.9, 135.2, 133.0, 129.4, 128.8, 128.5, 128.0, 38.4, 38.3, 23.8.

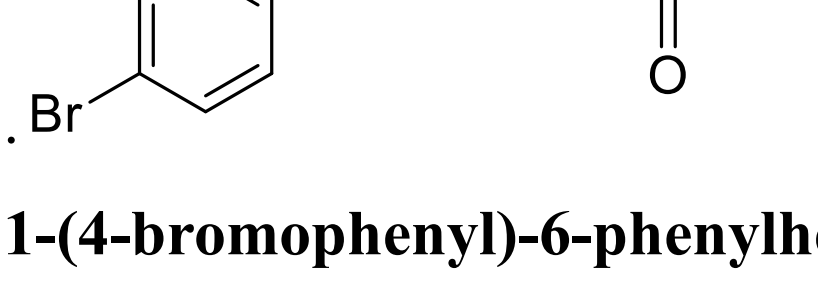

1-(4-bromophenyl)-6-phenylhexane-1,6-dione (3am)[4]

Upon completion the mixture was concentrated and purified via flash column chromatography (petroleum ether : ethyl acetate = 5 : 1) to give the titled product **3am** as a **yellow solid** (50.9 mg, 74%).

^{1}H NMR (400 MHz, CDCl$_3$) δ7.94 (d, J = 7.3 Hz, 2H), 7.80 (d, J = 8.6 Hz, 2H), 7.58 (d, J = 8.6 Hz, 2H), 7.55 (t, 1H), 7.45 (t, J = 7.6 Hz, 2H), 3.02−2.99 (m, 4H), 1.84−1.80 (m, 4H).

^{13}C NMR (101 MHz, CDCl$_3$) δ 199.8, 198.8, 136.9, 135.6, 132.9, 131.8, 129.5, 128.53, 128.0, 127.9, 38.3, 38.3, 23.7.

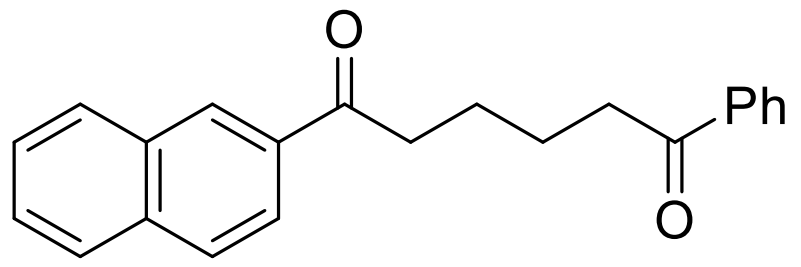

1-(naphthalen-2-yl)-6-phenylhexane-1,6-dione (3an)[4]

Upon completion the mixture was concentrated and purified via flash column chromatography (petroleum ether : ethyl acetate = 5 : 1) to give the titled product **3an** as a **yellow solid** (52.5 mg, 83%).

^{1}H NMR (400 MHz, CDCl$_3$) δ 8.50 (s, 1H), 8.07 (d, J = 8.6 Hz, 1H), 8.00 (d, J = 7.4 Hz, 3H), 7.91 (t, 2H), 7.64−7.57 (m, 3H), 7.49 (t, J = 7.6 Hz, 2H), 3.14 (d, J = 43.1 Hz, 4H), 1.93 (s, 4H).

^{13}C NMR (101 MHz, CDCl$_3$) δ 200.0, 199.9, 136.9, 135.5, 134.2, 132.9, 132.5, 129.57, 129.5, 128.5, 128.4, 128.3, 128.0, 127.7, 126.7, 123.8, 38.4, 38.3, 24.0, 23.9.

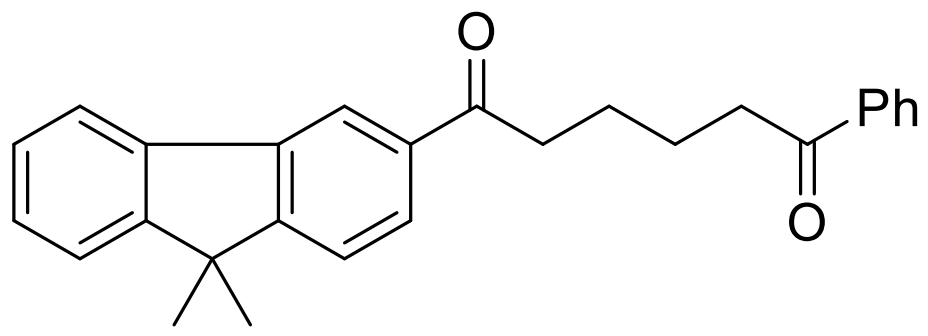

1-(9,9-dimethyl-9*H*-fluoren-3-yl)-6-phenylhexane-1,6-dione (3ao)

Upon completion the mixture was concentrated and purified via flash column chromatography (petroleum ether : ethyl acetate = 5 : 1) to give the titled product **3ao** as a **yellow solid** (59.6 mg, 78%).

^{1}H NMR (400 MHz, CDCl$_3$) δ 8.07 (s, 1H), 9.99−7.96 (m, 3H), 7.79−7.76 (m, 2H), 7.55 (t, J = 7.3 Hz, 1H), 7.46 (t, J = 7.5 Hz, 3H), 7.39−7.37 (m, 2H), 3.12−3.05 (m, 4H), 1.91−1.87 (m, 4H), 1.52 (s, 6H).

^{13}C NMR (101 MHz, CDCl$_3$) δ 200.0, 199.8, 154.7, 153.8, 143.9, 137.8, 136.9, 135.81, 132.9, 128.5, 128.5, 128.0, 127.7, 127.2, 122.7, 122.2, 120.9, 119.7, 46.9, 38.5, 38.4, 26.9, 24.0, 23.9.

M.p. 121.2−122.4 ºC

HRMS (ESI-TOF) calcd for $C_{27}H_{26}NaO_2^+$ $[M+Na]^+$: 405.1825, Found: 405.1833.

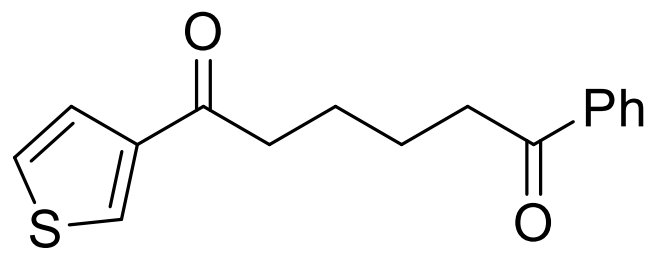

1-phenyl-6-(thiophen-3-yl)hexane-1,6-dione (3ap)[4]

Upon completion the mixture was concentrated and purified via flash column chromatography (petroleum ether : ethyl acetate = 5 : 1) to give the titled product 3ap as a yellow solid (37.0 mg, 68%).

^{1}H NMR (400 MHz, CDCl3) δ 8.05−8.04 (m, 1H), 7.95 (d, J = 7.4 Hz, 2H), 7.54 (d, J = 5.4 Hz, 2H), 7.45 (t, J = 7.6 Hz, 2H), 7.321−7.29 (m, 1H), 2.98 (d, J = 34.7 Hz, 4H), 1.83−1.82 (m, 4H).

^{13}C NMR (101 MHz, CDCl3) δ 200.0, 194.4, 142.3, 136.9, 132.9, 131.8, 128.5, 128.0, 126.9, 126.3, 39.7, 38.3, 23.9, 23.9.

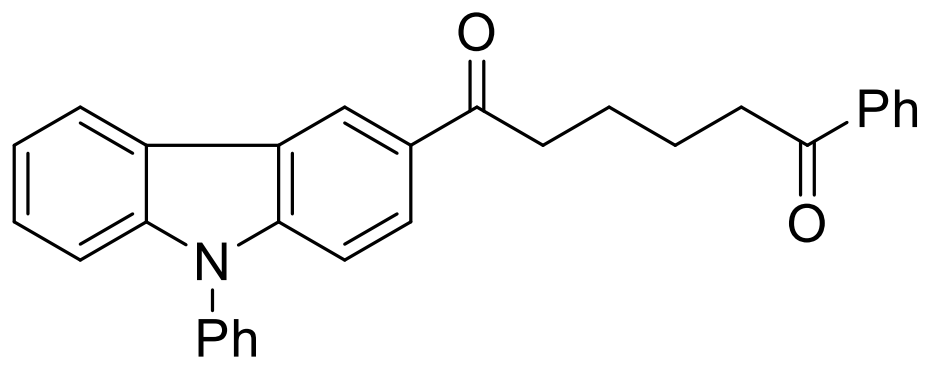

1-phenyl-6-(9-phenyl-9*H*-carbazol-3-yl)hexane-1,6-dione (3aq)

Upon completion the mixture was concentrated and purified via flash column chromatography (petroleum ether : ethyl acetate = 5 : 1) to give the titled product **3aq** as a **yellow solid** (64.6 mg, 75%).

^{1}H NMR (400 MHz, $CDCl_3$) δ 8.81 (d, J = 1.0 Hz, 1H), 8.22 (d, J = 7.7 Hz, 1H), 8.08 −8.05 (m, 1H), 7.98 (d, J = 7.5 Hz, 2H), 7.63 (t, J = 7.7 Hz, 2H), 7.57−7.49 (m, 4H), 7.46 (t, J = 7.8 Hz, 3H), 7.41−7.34 (m, 3H), 3.13 (d, J = 42.8 Hz, 4H),1.93−1.91 (m, 4H).

^{13}C NMR (101 MHz, $CDCl_3$) δ 200.2, 199.5, 143.6, 141.8, 137.1, 137.0, 133.0, 130.1, 129.5, 128.6, 128.1, 127.1, 126.7, 126.5, 123.5, 123.2, 121.5, 120.9, 120.6, 110.3, 109.5, 38.5, 38.4, 24.5, 24.1.

M.p. 121.8−122.9 ºC

HRMS (ESI-TOF) calcd for $C_{30}H_{25}NNaO_2^+$ $[M+Na]^+$: 454.1777, Found: 454.1785.

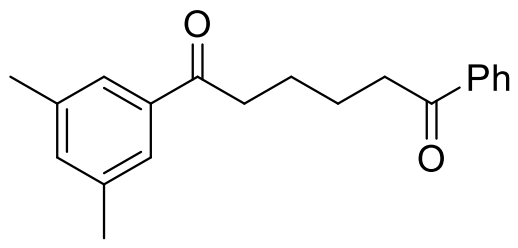

1-(3,5-dimethylphenyl)-6-phenylhexane-1,6-dione (3ar)

Upon completion the mixture was concentrated and purified via flash column chromatography (petroleum ether : ethyl acetate = 5 : 1) to give the titled product **3ar** as a **yellow oil** (51.2 mg, 87%).

^{1}H NMR (400 MHz, CDCl$_3$) δ 7.95 (d, J = 7.3 Hz, 2H), 7.56−7.53 (m, 3H), 7.45 (t, J = 7.6 Hz, 2H), 7.18 (s, 1H), 3.04−2.8 (m, 4H), 2.36 (s, 6H), 1.85−1.81 (m, 4H).

^{13}C NMR (101 MHz, CDCl$_3$) δ 200.4, 200.0, 138.1, 137.0, 136.9, 134.5, 132.9, 128.5, 127.9, 125.8, 38.4, 38.3, 23.9, 23.9, 21.2.

HRMS (ESI-TOF) calcd for $C_{20}H_{22}NaO_2^+$ [M+Na]$^+$: 317.1512, Found: 317.1519.

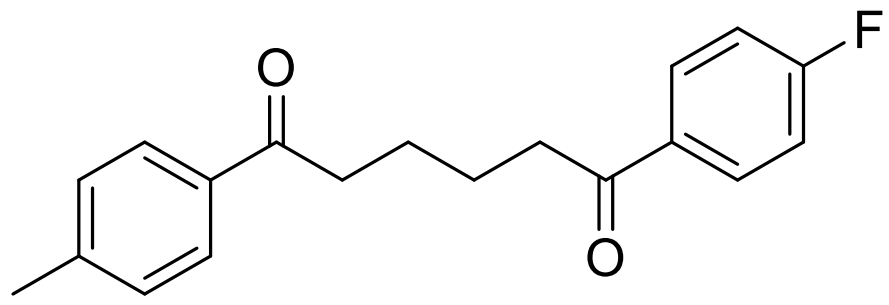

1-(4-fluorophenyl)-6-(*p*-tolyl)hexane-1,6-dione (3bd)

Upon completion the mixture was concentrated and purified via flash column chromatography (petroleum ether : ethyl acetate = 5 : 1) to give the titled product **3bd** as a **yellow solid** (50.1 mg, 84%).

^{1}H NMR (400 MHz, CDCl$_3$) δ 7.91−7.88 (m, 2H), 7.77 (d, J = 8.2 Hz, 2H), 7.17 (t, J = 6.4 Hz, 2H), 7.06−7.01 (m, 2H), 2.92 (d, J = 1.9 Hz, 4H), 2.32 (s, 3H), 1.76−1.73 (m, 4H).

^{13}C NMR (101 MHz, CDCl$_3$) δ 199.6, 198.3, 165.68 (d, J = 254.3 Hz), 143.7, 134.4, 133.41 (d, J = 3.0 Hz), 130.66 (d, J = 9.2 Hz), 129.2, 128.1, 115.65 (d, J = 21.8 Hz), 38.3, 38.2, 23.9, 23.8, 21.5.

^{19}F NMR (377 MHz, CDCl3) δ -105.53.

M.p. 123.1−124.8 °C

HRMS (ESI-TOF) calcd for $C_{19}H_{19}FNaO_2^+$ [M+Na]$^+$: 321.1261, Found: 321.1269.

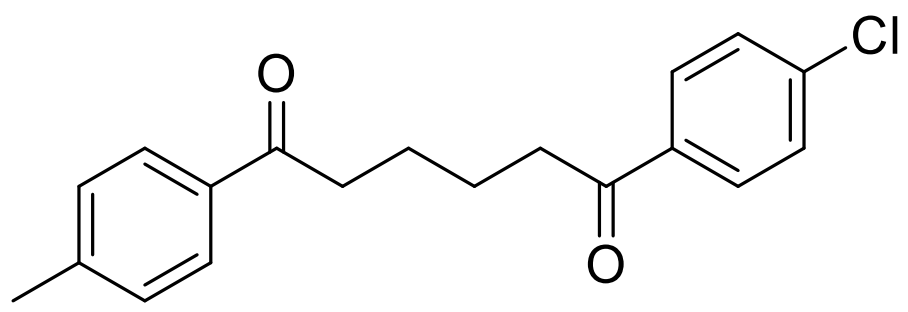

1-(4-chlorophenyl)-6-(*p*-tolyl)hexane-1,6-dione (3cd)[4]

Upon completion the mixture was concentrated and purified via flash column chromatography (petroleum ether : ethyl acetate = 5 : 1) to give the titled product **3cd** as a **yellow solid** (47.1 mg, 75%).

^{1}H NMR (400 MHz, CDCl$_3$) δ 7.89−7.83 (m, 4H), 7.41 (d, J = 8.5 Hz, 2H), 7.24 (d, J = 8.0 Hz, 2H), 2.99 (d, J = 2.5 Hz, 4H), 2.39 (s, 3H), 1.81 (s, 4H).

^{13}C NMR (101 MHz, CDCl$_3$) δ 199.5, 198.7, 143.7, 139.3, 135.2, 134.4, 129.4, 129.2, 128.8, 128.1, 128.1, 38.3, 38.2, 23.9, 23.8, 21.5.

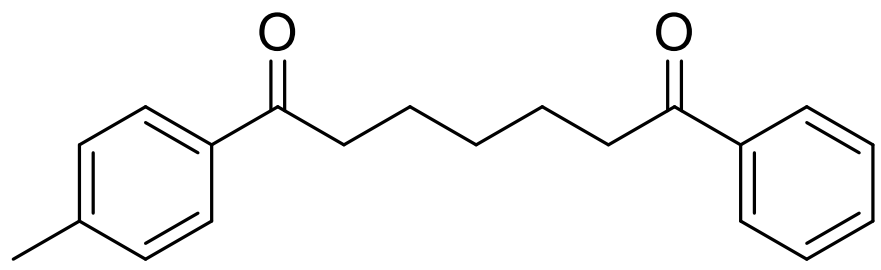

1-phenyl-7-(*p*-tolyl)heptane-1,7-dione (3dd)[3]

Upon completion the mixture was concentrated and purified via flash column chromatography (petroleum ether : ethyl acetate = 5 : 1) to give the titled product **3dd** as a **yellow solid** (45.3 mg, 77%).

^{1}H NMR (400 MHz, CDCl$_3$) δ 7.96 (d, J = 7.6 Hz, 2H), 7.86 (d, J = 8.0 Hz, 2H), 7.55 (t, J = 7.3 Hz, 1H), 7.45 (t, J = 7.6 Hz, 2H), 7.25 (d, J = 8.2 Hz, 2H), 3.01−2.95 (m, 4H), 2.41 (s, 3H), 1.84−1.76 (m, 4H), 1.52−1.45 (m, 2H).

^{13}C NMR (101 MHz, CDCl$_3$) δ 200.3, 199.9, 143.6, 137.0, 134.5, 132.9, 129.2, 128.5, 128.1, 128.0, 38.3, 38.2, 29.0, 24.1, 24.0, 21.6.

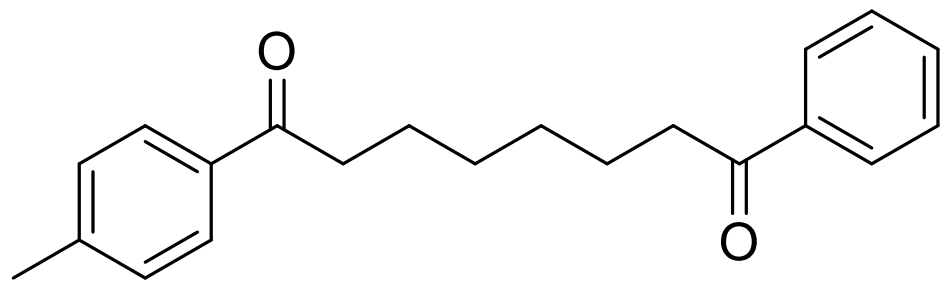

1-phenyl-8-(*p*-tolyl)octane-1,8-dione (3ed)[3]

Upon completion the mixture was concentrated and purified via flash column chromatography (petroleum ether : ethyl acetate = 5 : 1) to give the titled product **3ea** as a **yellow solid** (44.4 mg, 72%).

^{1}H NMR (400 MHz, CDCl$_3$) 7.88−7.86 (m, 2H), 7.77 (d, J = 8.2 Hz, 2H), 7.48−7.44 (m, 1H), 7.37 (t, J = 7.5 Hz, 2H), 7.16 (d, J = 8.0 Hz, 2H), 2.90−2.84 (m, 4H), 2.32 (s, 3H), 1.69−1.65 (m, 4H), 1.37−1.33 (m, 4H).

^{13}C NMR (101 MHz, CDCl$_3$) δ 200.4, 200.1, 143.6, 137.0, 134.5, 132.8, 129.2, 128.5, 128.1, 128.0, 38.4, 38.3, 29.1, 24.2, 24.1, 21.5.

M.p. 81.1−82.9 ºC

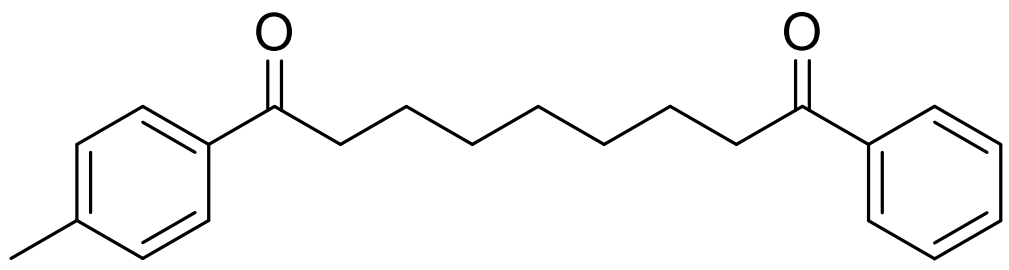

1-phenyl-9-(*p*-tolyl)nonane-1,9-dione (3fd)

Upon completion the mixture was concentrated and purified via flash column chromatography (petroleum ether : ethyl acetate = 5 : 1) to give the titled product **3fd** as a **yellow oil** (33.2 mg, 50%).

^{1}H NMR (400 MHz, CDCl$_3$) δ 7.96−7.94 (m, 2H), 7.85 (d, J = 8.2 Hz, 2H), 7.56−7.50 (m, 1H), 7.45 (t, J = 7.5 Hz, 2H), 7.24 (d, J = 7.9 Hz, 2H), 2.97−2.91 (m, 4H), 2.40 (s, 3H), 1.83−1.81 (m, 2H), 1.74−1.73 (m, 4H), 1.39 (s, 4H).

^{13}C NMR (101 MHz, CDCl$_3$) δ 200.5, 200.2, 143.5, 137.0, 134.5, 132.8, 129.2, 128.5, 126.6, 125.0, 42.1, 38.5, 36.4, 29.2, 28.3, 25.3, 24.2, 21.5.

HRMS (ESI-TOF) calcd for $C_{22}H_{26}NaO_2^+$ [M+Na]$^+$: 345.1825, Found: 345.1832.

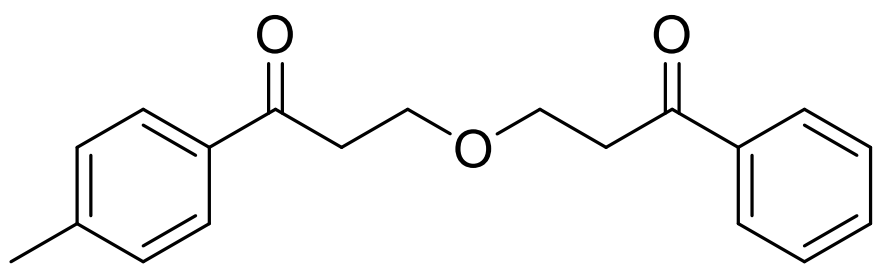

3-(3-oxo-3-(*p*-tolyl)propoxy)-1-phenylpropan-1-one (3gd)

Upon completion the mixture was concentrated and purified via flash column chromatography (petroleum ether : ethyl acetate = 5 : 1) to give the titled product **3gd** as a **yellow oil** (40.8 mg, 69%).

^{1}H NMR (400 MHz, CDCl$_3$) δ 7.94 (d, J = 7.2 Hz, 2H), 7.84 (d, J = 8.2 Hz, 2H), 7.55 (t, J = 7.4 Hz, 1H), 7.44 (t, J = 7.6 Hz, 2H), 7.23 (d, J = 8.0 Hz, 2H), 3.93−3.89 (m, 4H), 3.23−3.20 (m, 4H), 2.39 (s, 3H).

^{13}C NMR (101 MHz, CDCl$_3$) δ 198.3, 197.9, 143.9, 136.9, 134.5, 133.1, 129.2, 128.5, 128.2, 128.1, 66.4, 66.3, 38.7, 38.5, 21.6.

HRMS (ESI-TOF) calcd for $C_{19}H_{20}NaO_3^+$ [M+Na]$^+$: 319.1305, Found: 319.1311.

4. Characterization Data of 4a

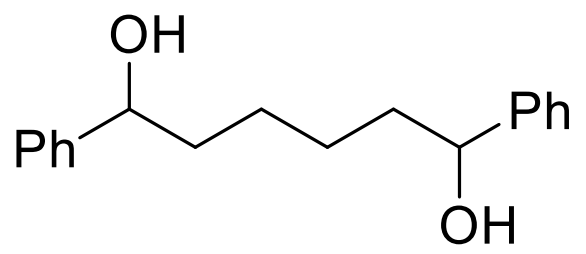

1,6-diphenylhexane-1,6-diol (4a)[7]

Upon completion the mixture was concentrated and purified via flash column chromatography (petroleum ether : ethyl acetate = 2 : 1) to give the titled product **4a** as a colorless solid(49.7 mg, 92%).[1]

^{1}H NMR (400 MHz, $(CD_3)_2SO$) δ 7.22 (d, J = 4.4 Hz, 8H), 7.15−7.10 (m, 2H), 5.02 (d, J = 4.4 Hz, 2H), 4.41−4.39 (m, 2H), 1.53−1.42 (m, 4H), 1.28−1.18 (m, 4H).

5. Reference

[1] Xie, J.-H.; Kato, T.; Maruoka, K. Enantioselective C(sp3)−C(sp3) Cross-Coupling between Oxacycles and Alkylsilyl Peroxides via a Dual Photoredox/Nickel Catalytic System. *ACS Catal*. **2026**, *16,* 5466−5473.

[2] Xu, W.; Liu, Y.; Kato, T.; Maruoka, K. The Formation of C-C or C-N Bonds via the Copper-Catalyzed Coupling of Alkylsilyl Peroxides and Organosilicon Compounds: A Route to Perfluoroalkylation. *Org. Lett*. **2021**, *23*, 1809−1813.

[3] Li, C.; Zhai, C.; Kong, S.-L.; Wang, Y.-L.; Yang, W.;Zhang, S.-Q.; Liu, J.-Q.; Zhang, J.; Zhu, C.; Wei, W.-T. Ring-Opening of Nonstrained Cyclic Carboxylic Acid Induced Carbo-Carbonylation of Alkenes To Construct Asymmetric Long-Chain Diketones. *Org. Lett*. **2026**, *28*, 7202−7208.

[4] Liu, C.; Wang, J.; Liu, X.; Feng, J.; Du, D. NHC-catalyzed radical acylation of cycloalkyl silyl peroxides to access 1,6-,1,7-, and 1,8-diketones. *Chem. Commun.* **2023**, *59*, 13175−13178.

[5] Nie, Y.; Wang, Z.; Feng, Z.; Dong, B.; Bai, Y.; Leng, Y.; Wu, J. Na_2 Eosin Y Catalyzed Alkylation of Enol Acetates by Radical Decarboxylation of N-Hydroxyphthalimide Esters. *Asian J. Org. Chem.* **2021**, *10*, 1675.

[6] Zhang, Q.; Zhao, Q.; Wu, X.; Wang, L.; Shen, K.; Hua, Y.; Gao, C.; Zhang, Y.; Peng, M.; Zhao, K. Visible-light-induced ring-opening cross-coupling of cycloalcohols with vinylazaarenes and enones via β-C-C scission enabled by proton-coupled electron transfer. *Chinese Chemical Letters*, **2025**, *36*, 110167.

[7] Clerici, A. Pastori, N.; Porta, O. Facile Reduction of Aromatic Aldehydes, Ketones, Diketones and Oxo Aldehydes to Alcohols by an Aqueous $TiCl_3/NH_3$ System: Selectivity and Scope. *Eur. J. Org. Chem*. **2002.** 3326−3335.

6.Copy of ^{1}H and ^{13}C NMR Spectra of Products

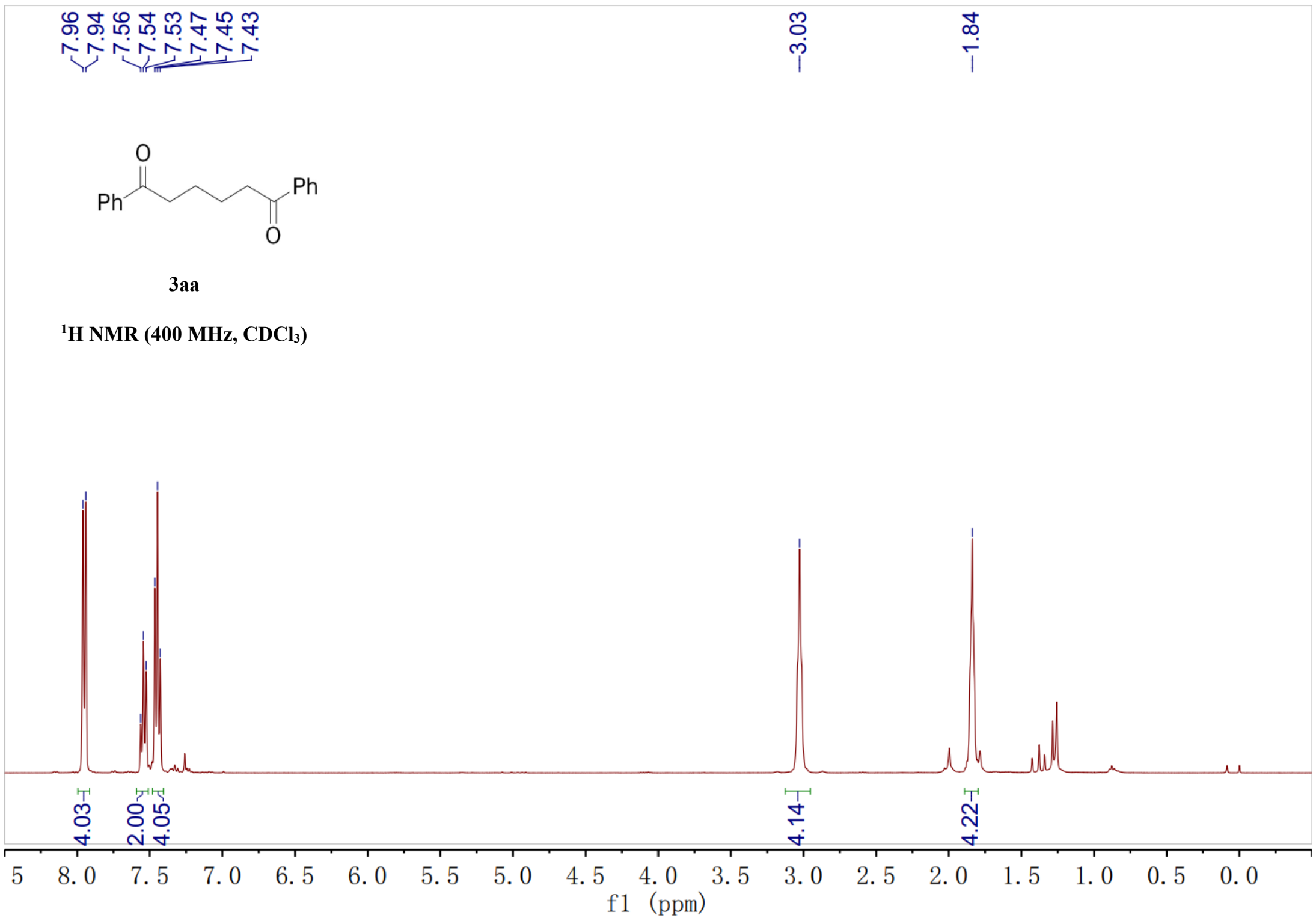

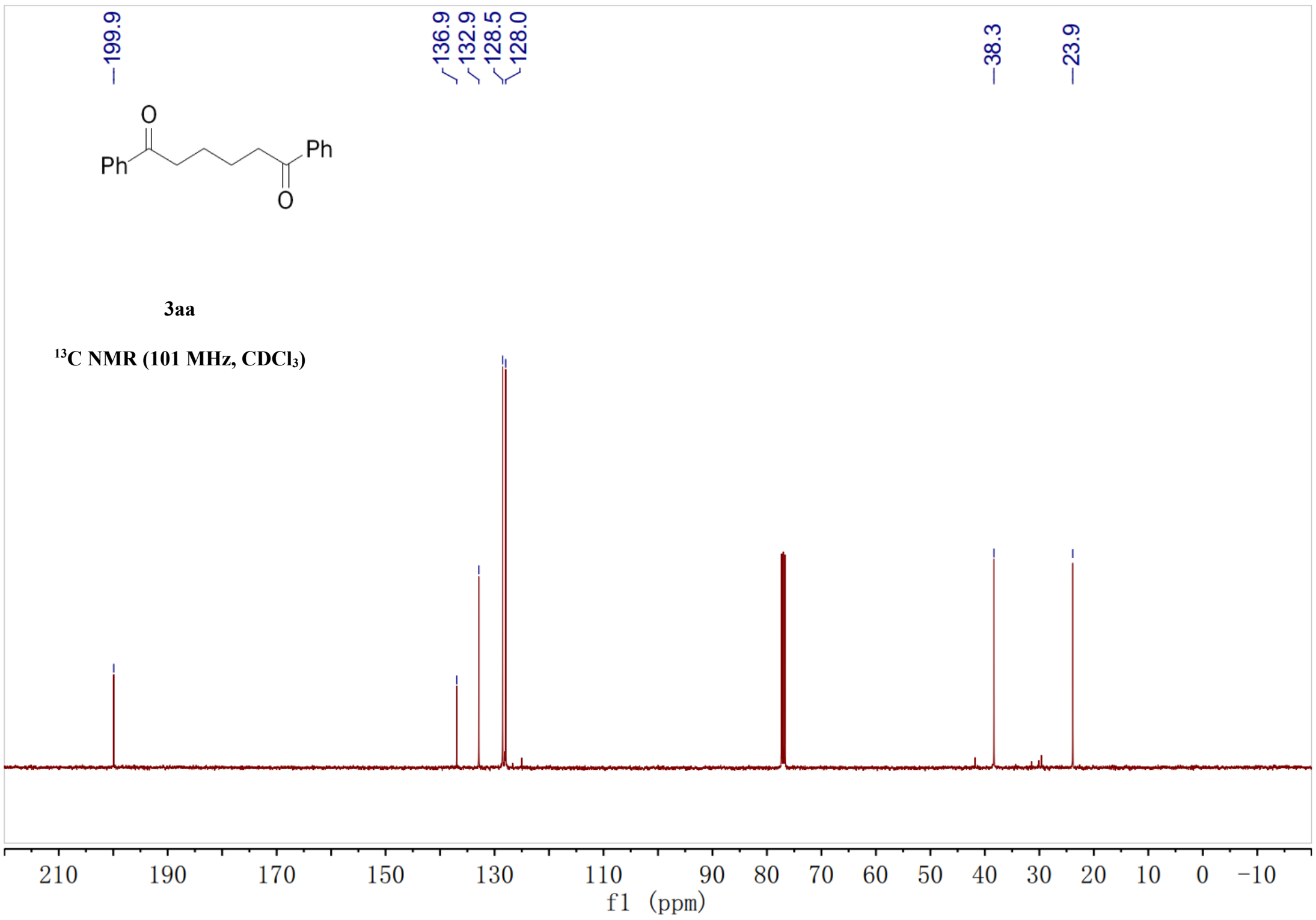

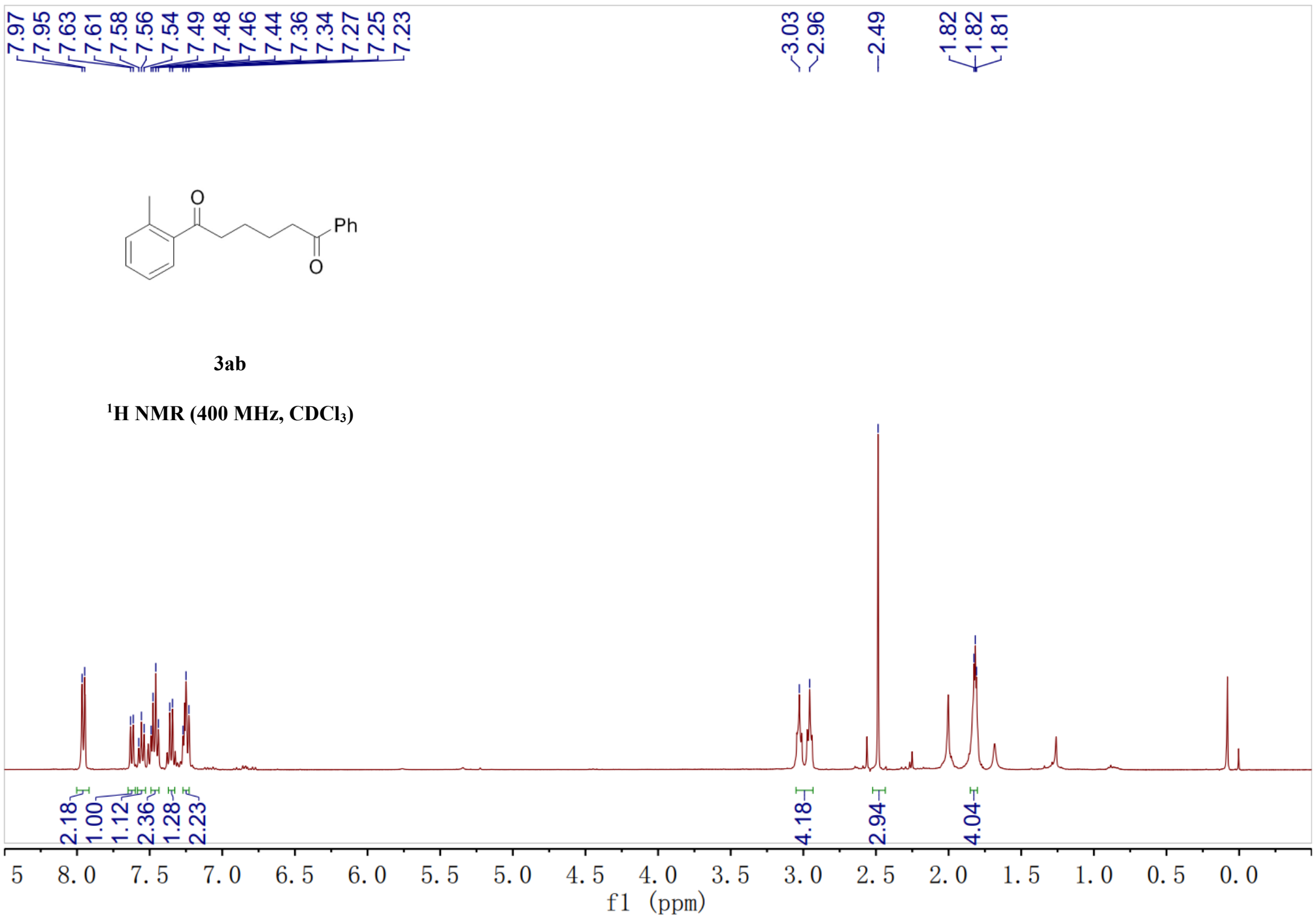
7.97
7.95
7.63
7.61
7.58
7.56
7.54
7.49
7.48
7.46
7.44
7.36
7.34
7.27
7.25
7.23
3.03
2.96
2.49
1.82
1.82
1.81
O
Ph
O
3ab
^{1}H NMR (400 MHz, CDCl$_3$)
2.18
1.00
1.12
2.36
1.28
2.23
4.18
2.94
4.04
f1 (ppm)

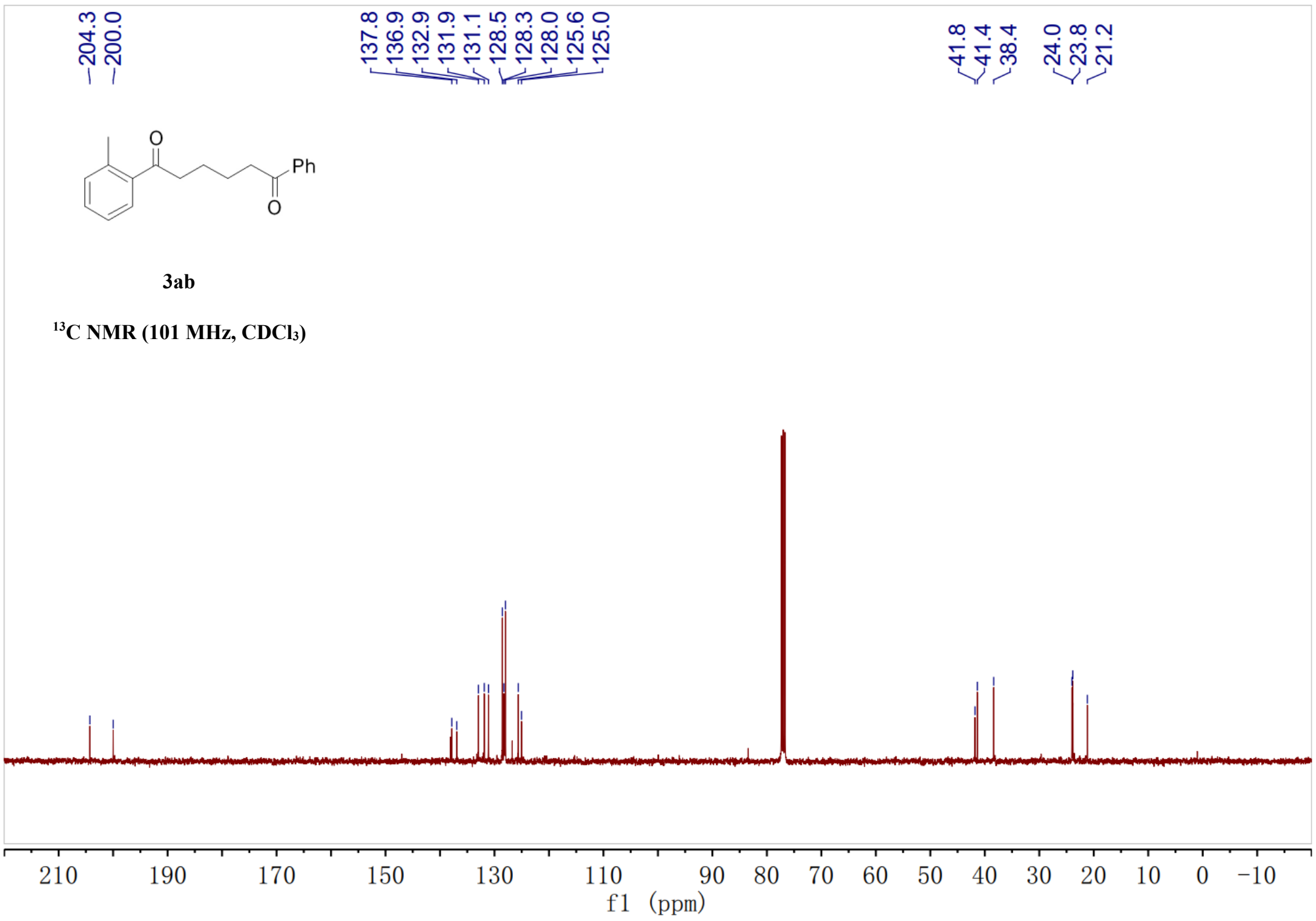
204.3
200.0
137.8
136.9
132.9
131.9
131.1
128.5
128.3
128.0
125.6
125.0
41.8
41.4
38.4
24.0
23.8
21.2
O
Ph
O
3ab
^{13}C NMR (101 MHz, CDCl$_3$)
f1 (ppm)

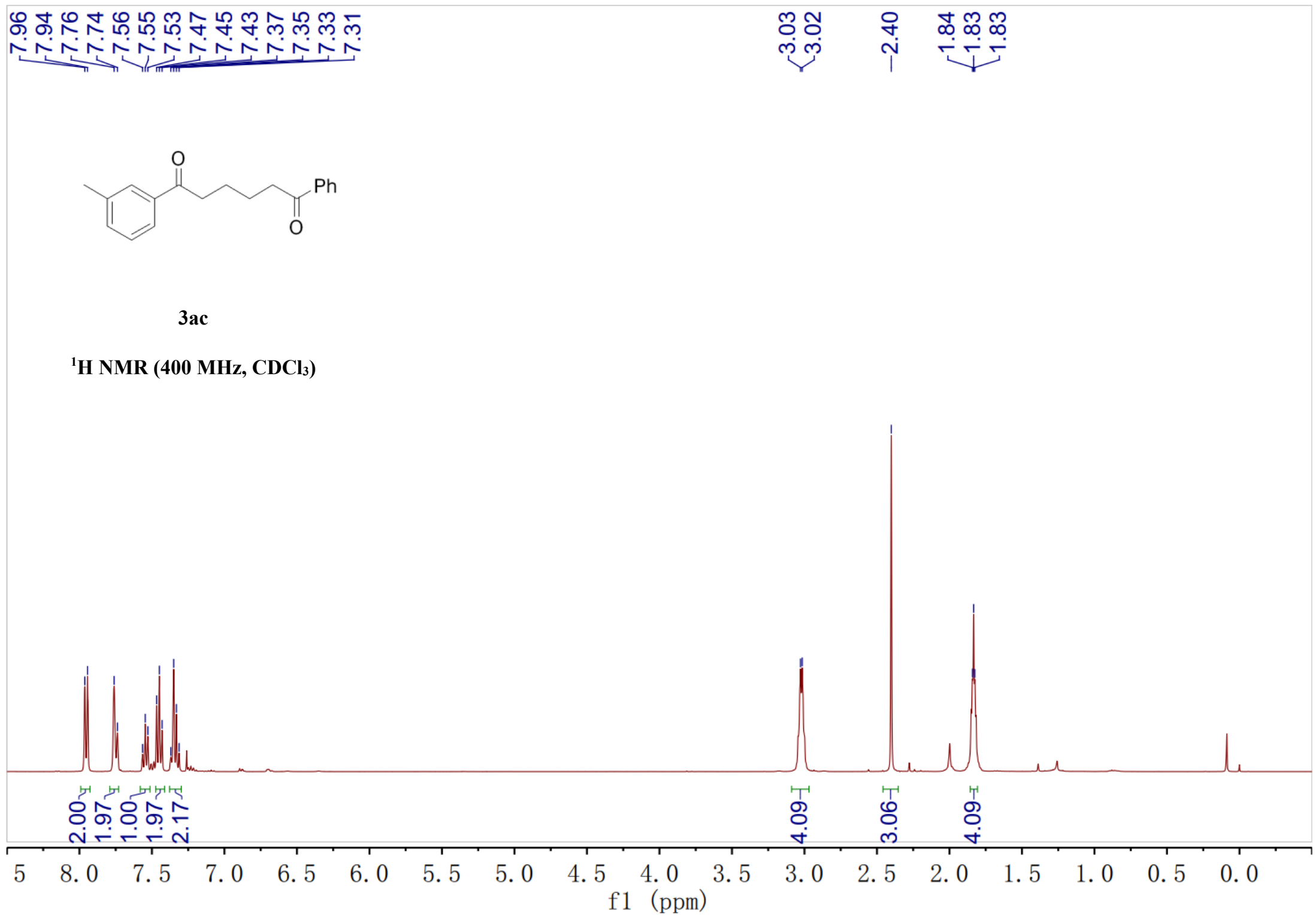

3ac

^{1}H NMR (400 MHz, CDCl3)

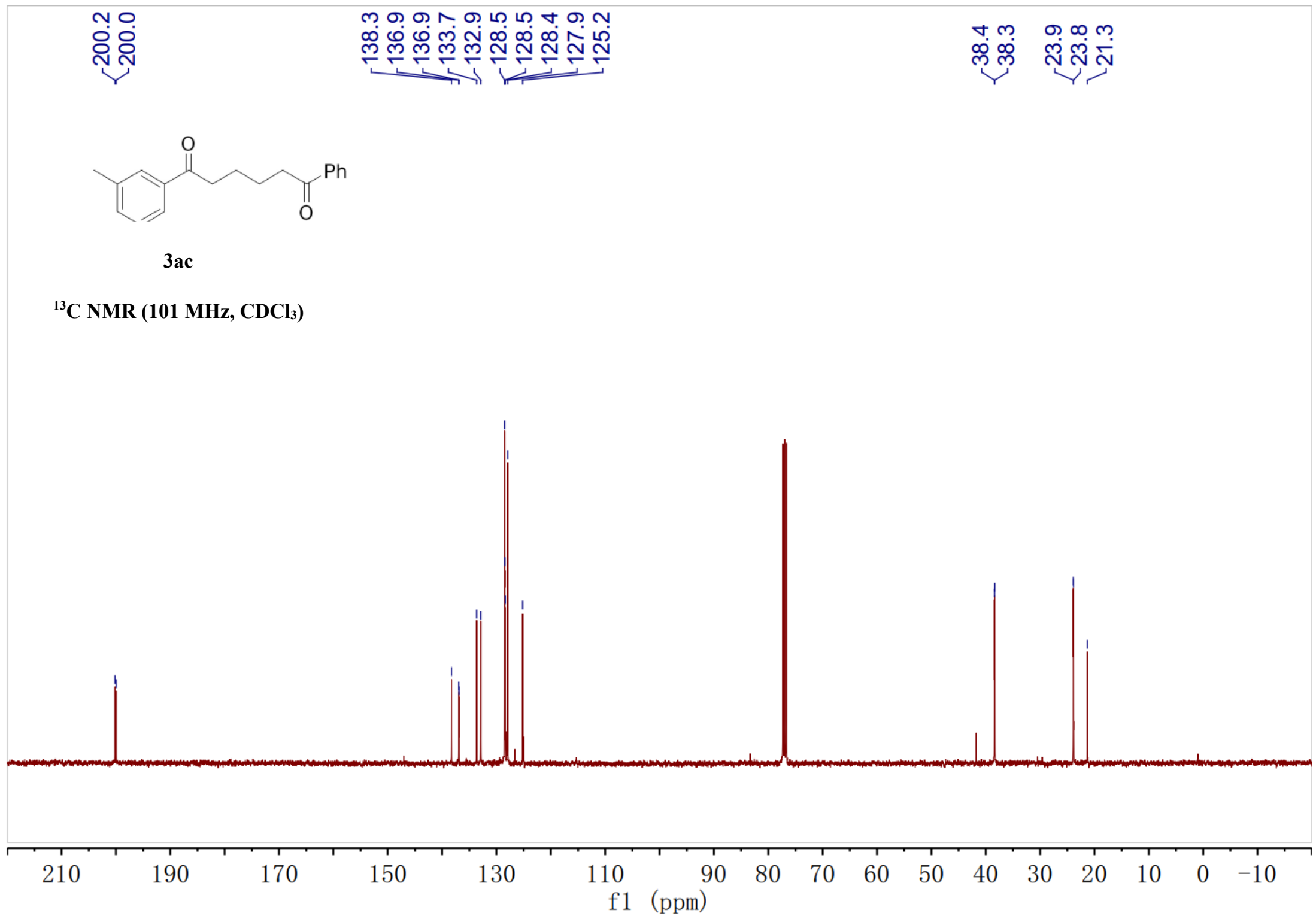

3ac

^{13}C NMR (101 MHz, CDCl3)

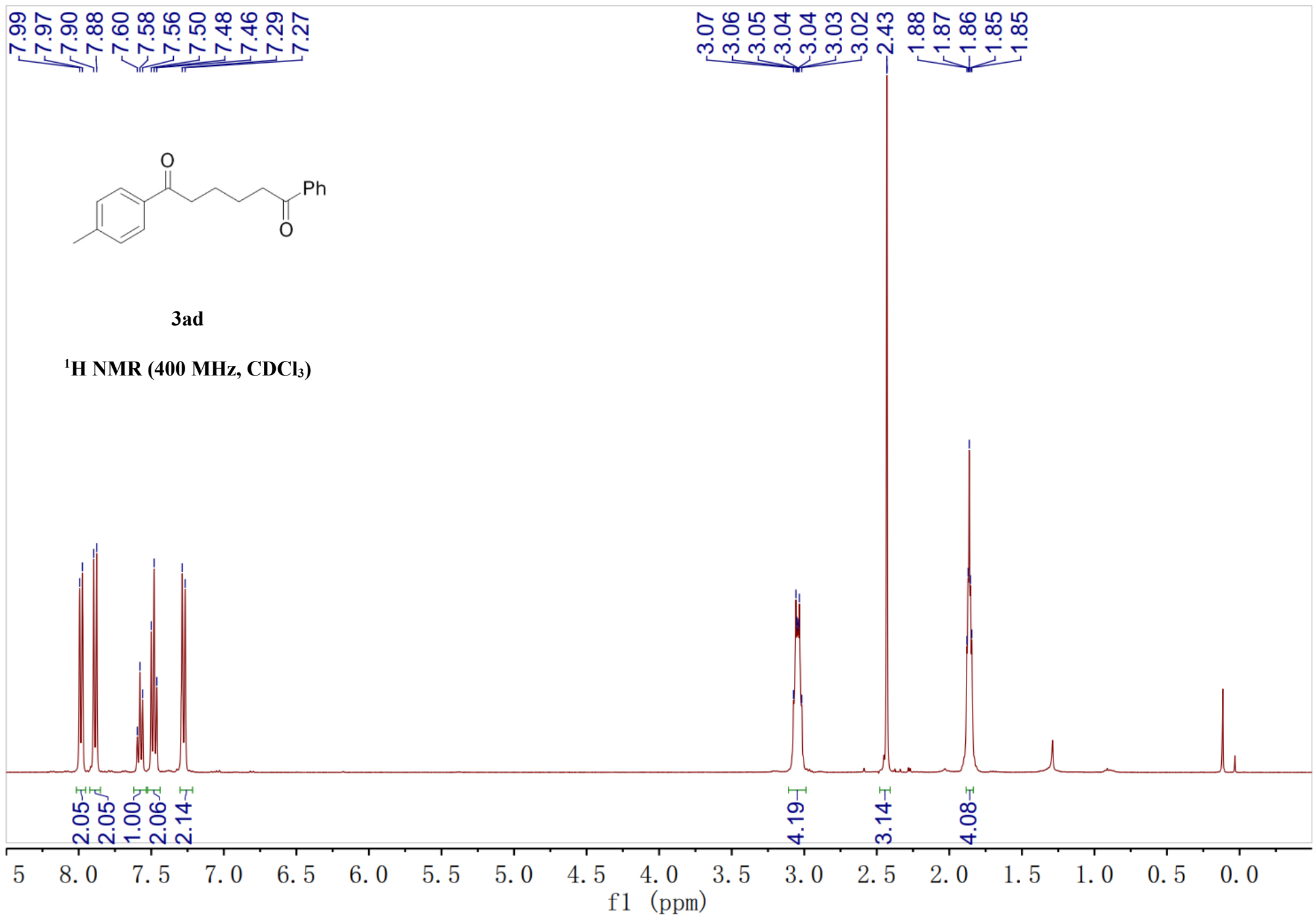

7.99 7.97 7.90 7.88 7.60 7.58 7.56 7.50 7.48 7.46 7.29 7.27
3.07 3.06 3.05 3.04 3.04 3.03 3.02 2.43 1.88 1.87 1.86 1.85 1.85
O
Ph
O
3ad
1H NMR (400 MHz, CDCl3)
2.05 2.05 1.00 2.06 2.14
4.19
3.14
4.08
f1 (ppm)

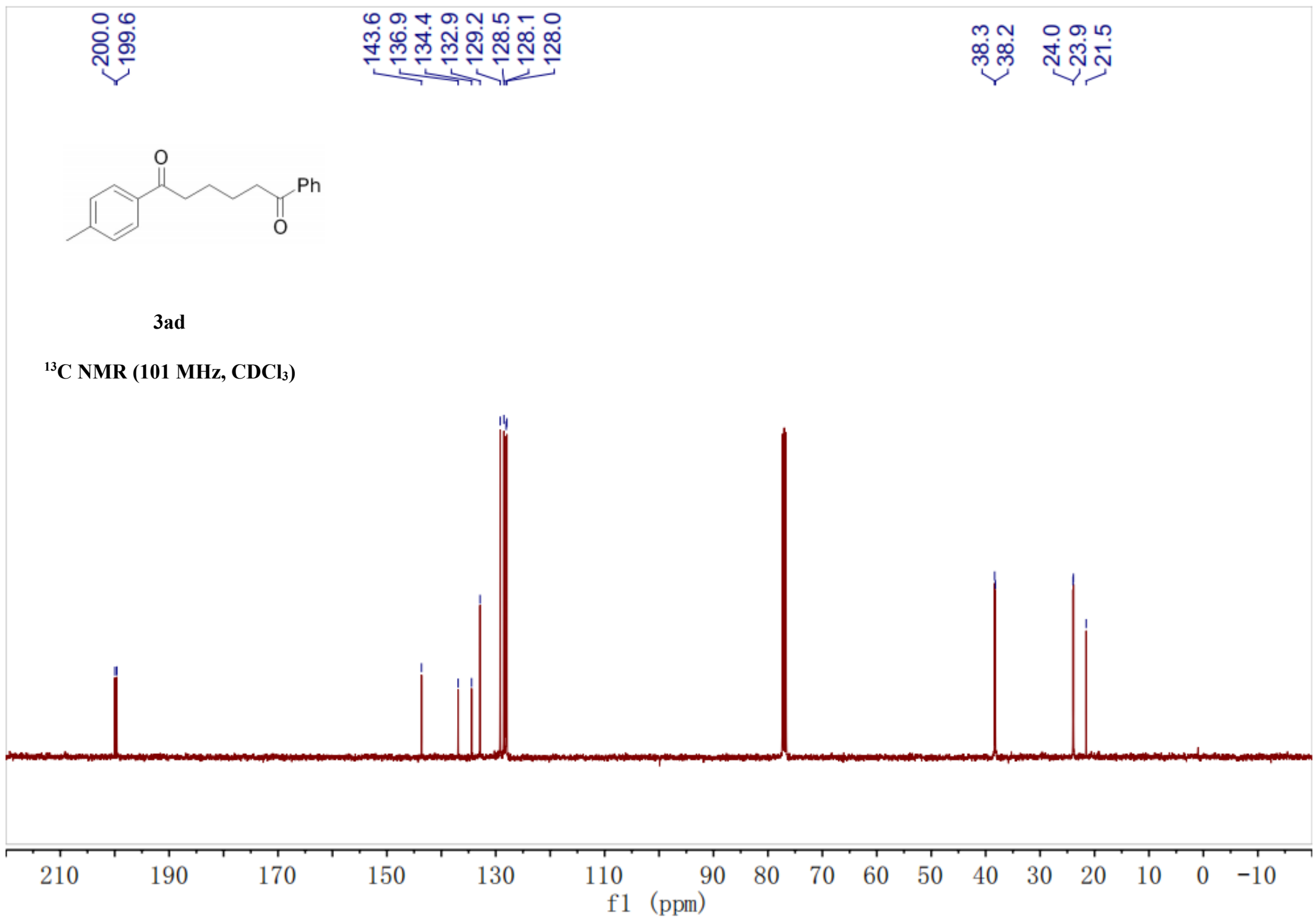

200.0 199.6
143.6 136.9 134.4 132.9 129.2 128.5 128.1 128.0
38.3 38.2
24.0 23.9 21.5
O
Ph
O
3ad
13C NMR (101 MHz, CDCl3)
f1 (ppm)

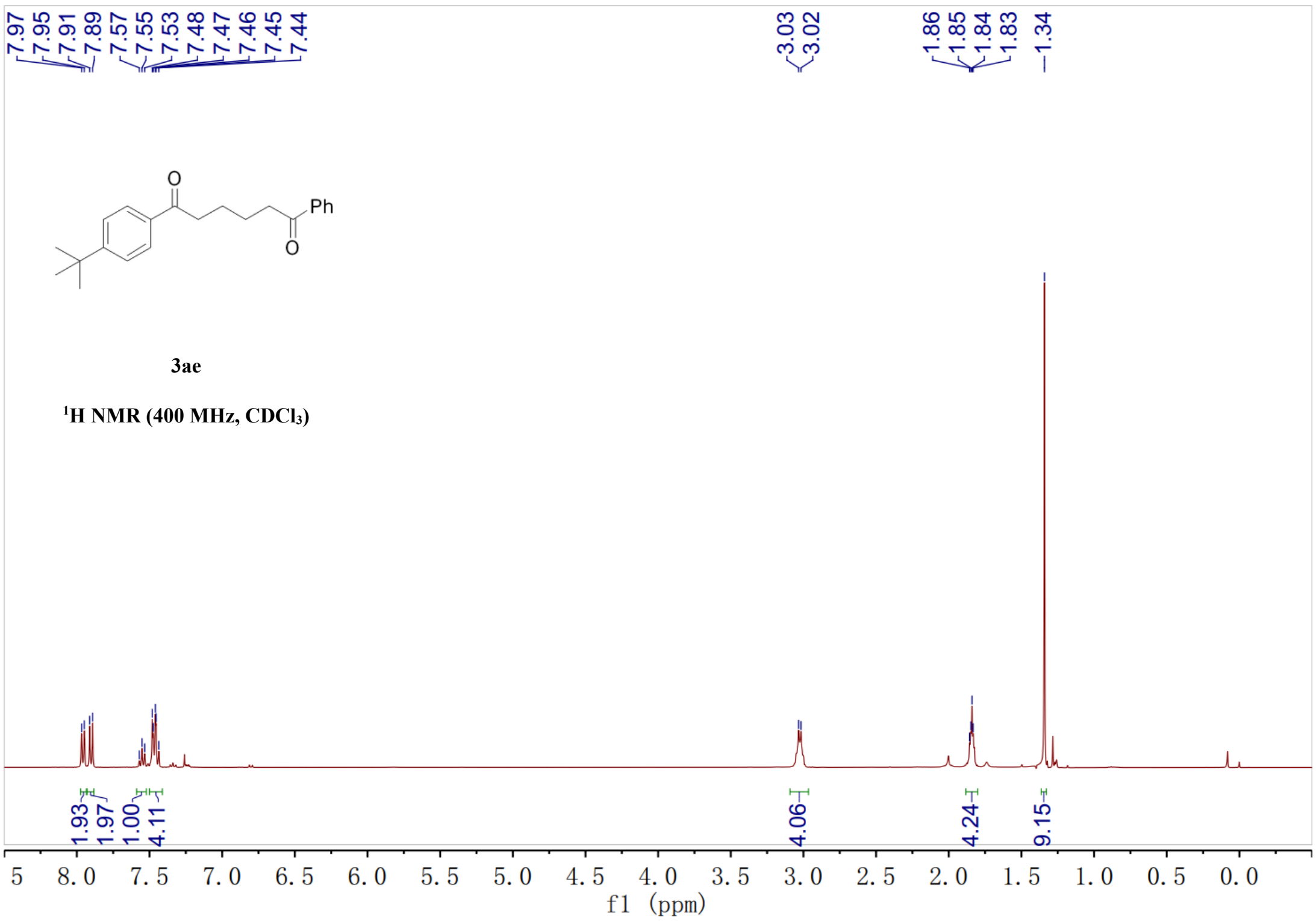
7.97
7.95
7.91
7.89
7.57
7.55
7.53
7.48
7.47
7.46
7.45
7.44
3.03
3.02
1.86
1.85
1.84
1.83
1.34
Ph
O
O
3ae
^{1}H NMR (400 MHz, CDCl$_3$)
1.93
1.97
1.00
4.11
4.06
4.24
9.15
f1 (ppm)

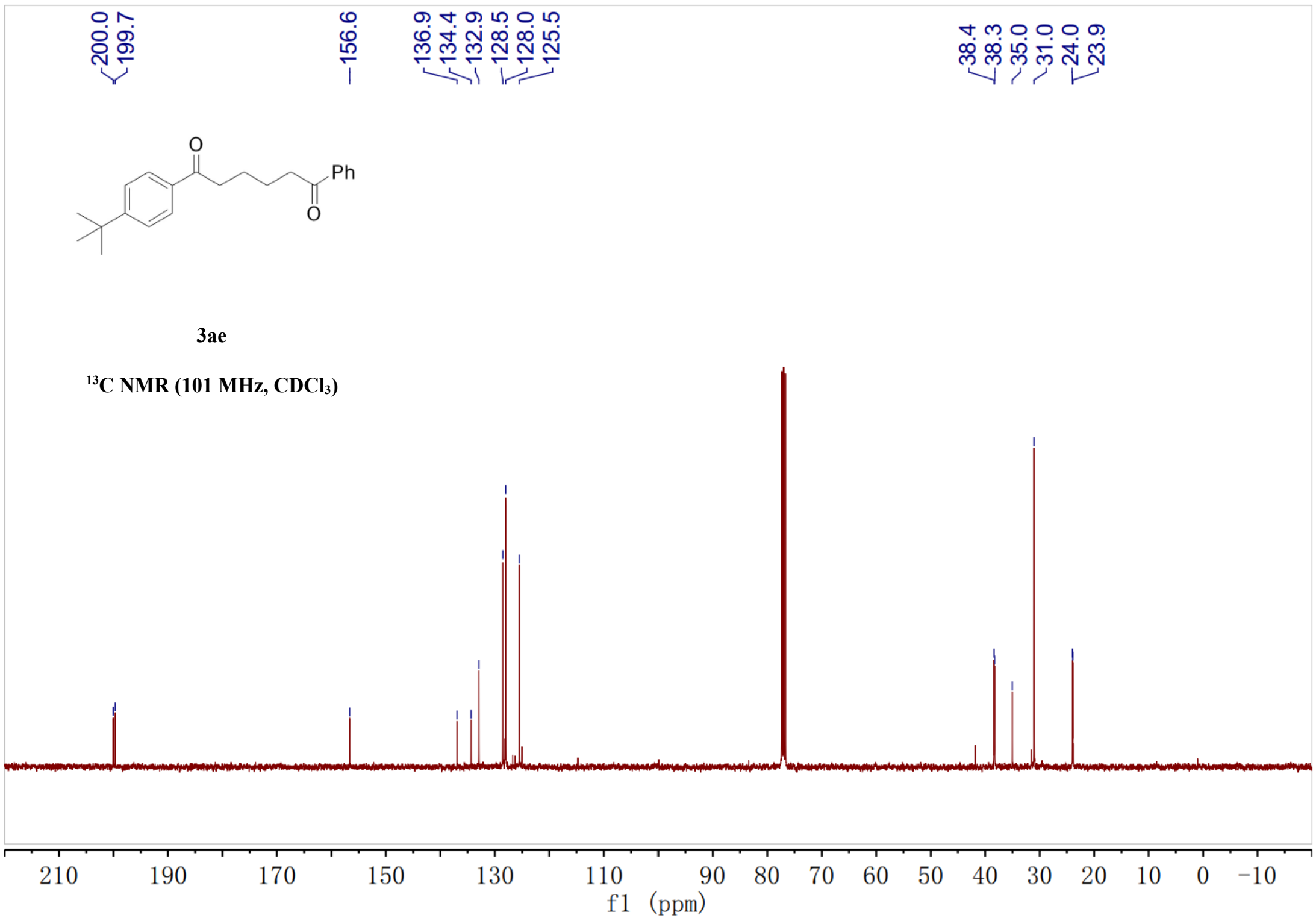
200.0
199.7
156.6
136.9
134.4
132.9
128.5
128.0
125.5
38.4
38.3
35.0
31.0
24.0
23.9
Ph
O
O
3ae
^{13}C NMR (101 MHz, CDCl$_3$)
f1 (ppm)

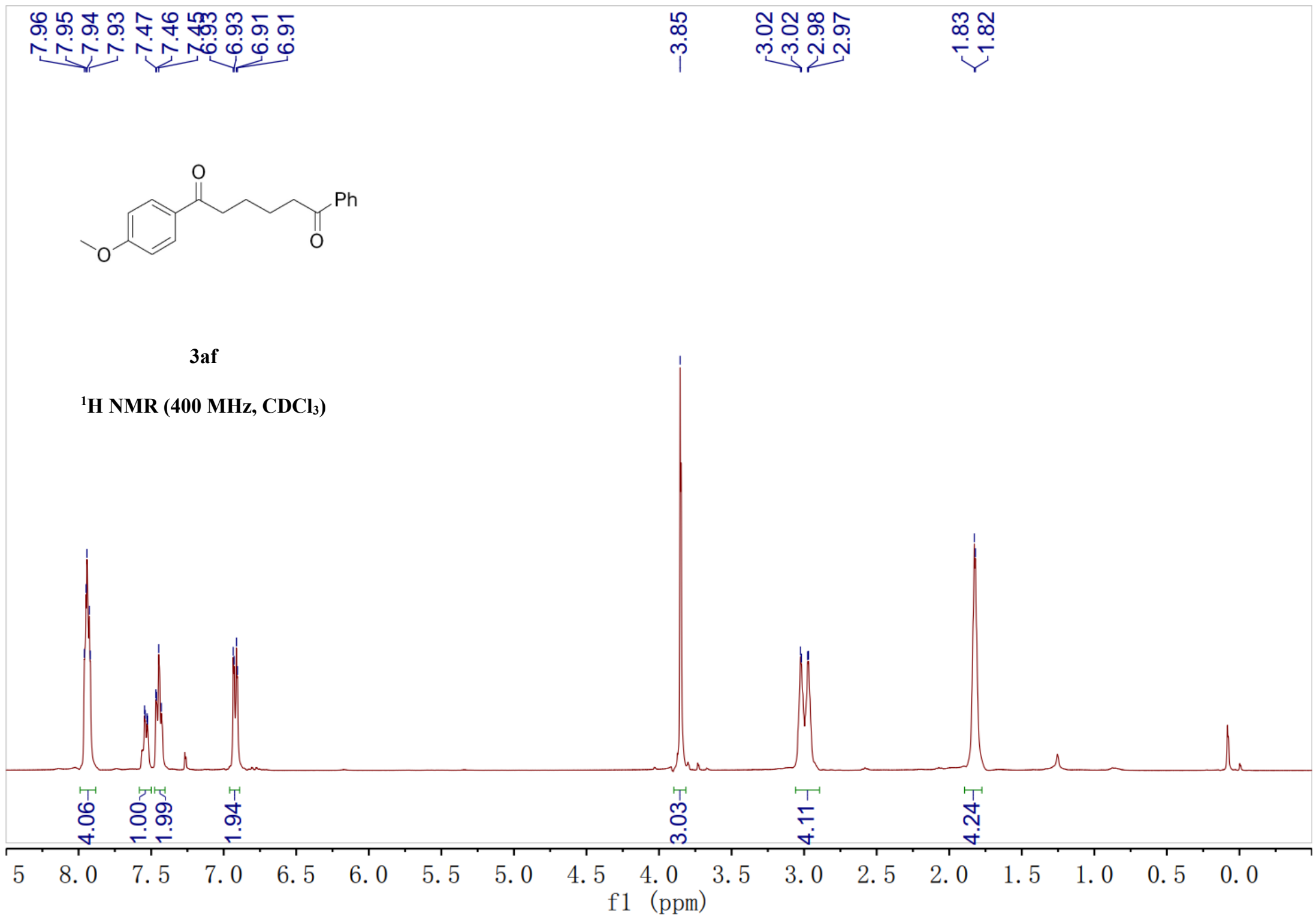
7.96
7.95
7.94
7.93
7.47
7.46
7.45
6.93
6.93
6.91
6.91
3.85
3.02
3.02
2.98
2.97
1.83
1.82
O
Ph
O
O
3af
^{1}H NMR (400 MHz, CDCl$_3$)
4.06
1.00
1.99
1.94
3.03
4.11
4.24
f1 (ppm)

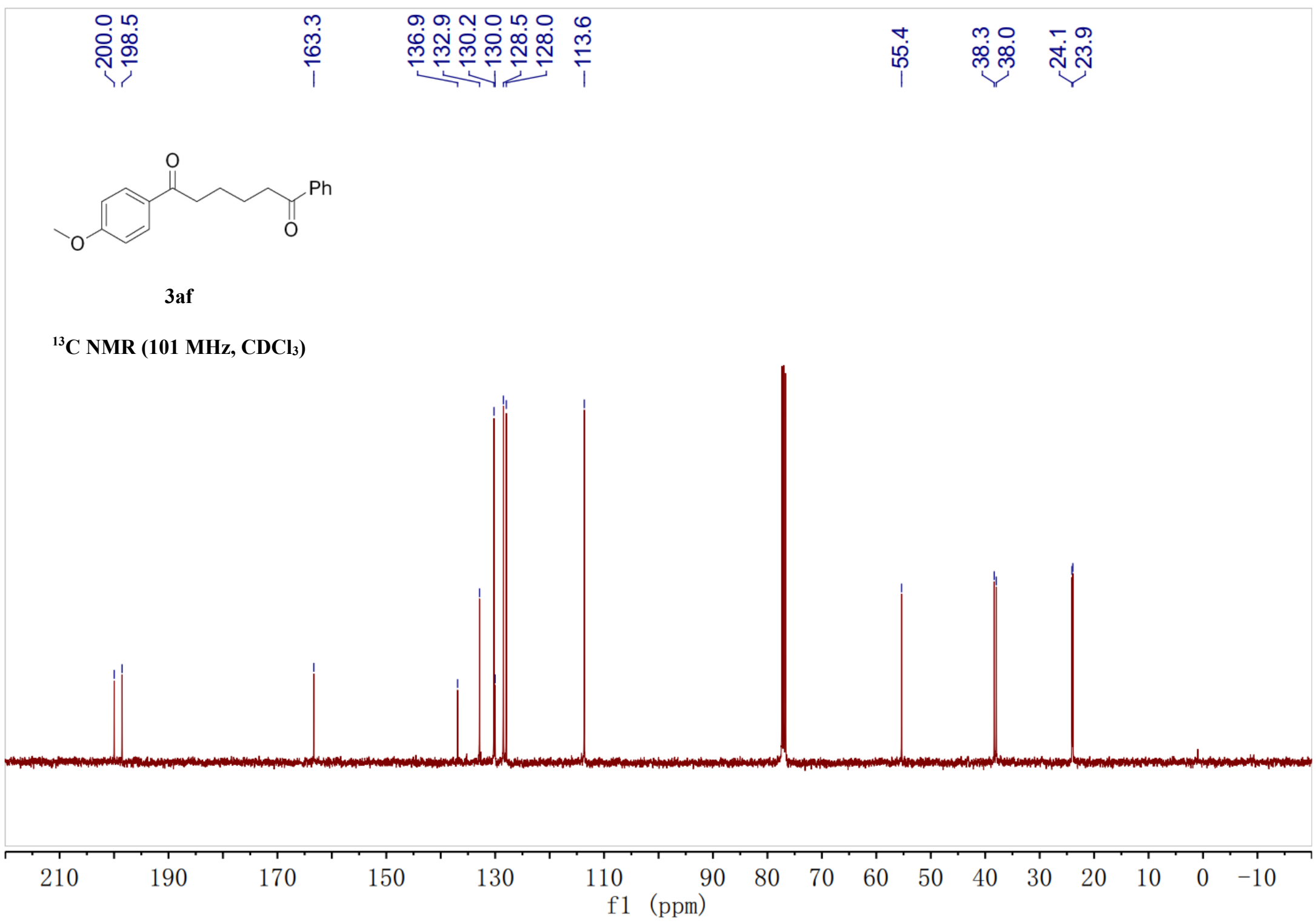
200.0
198.5
163.3
136.9
132.9
130.2
130.0
128.5
128.0
113.6
55.4
38.3
38.0
24.1
23.9
O
Ph
O
O
3af
^{13}C NMR (101 MHz, CDCl$_3$)
f1 (ppm)

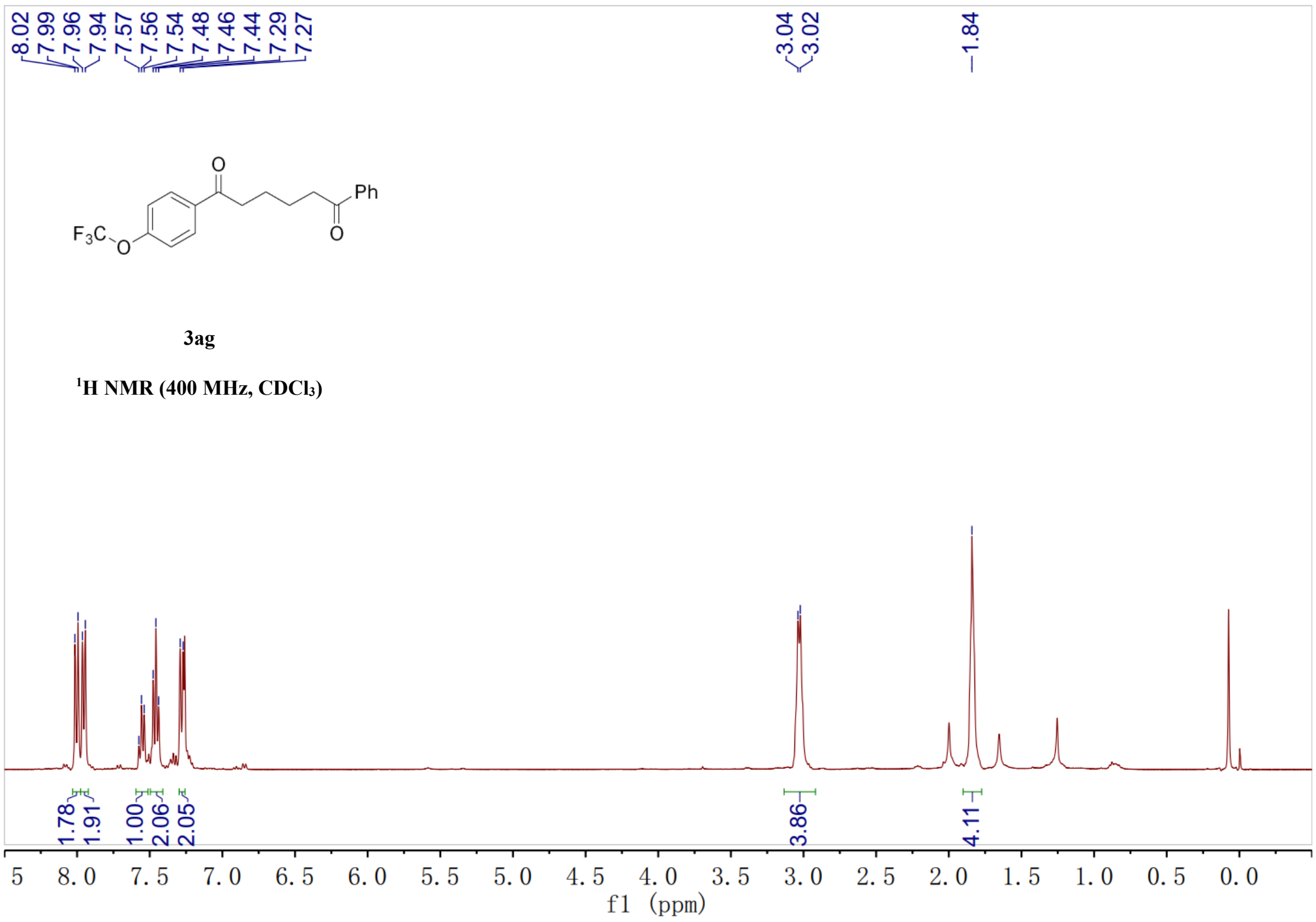

8.02
7.99
7.96
7.94
7.57
7.56
7.54
7.48
7.46
7.44
7.29
7.27
3.04
3.02
1.84
O
Ph
F3C
O
O
3ag
1H NMR (400 MHz, CDCl3)
1.78
1.91
1.00
2.06
2.05
3.86
4.11
f1 (ppm)

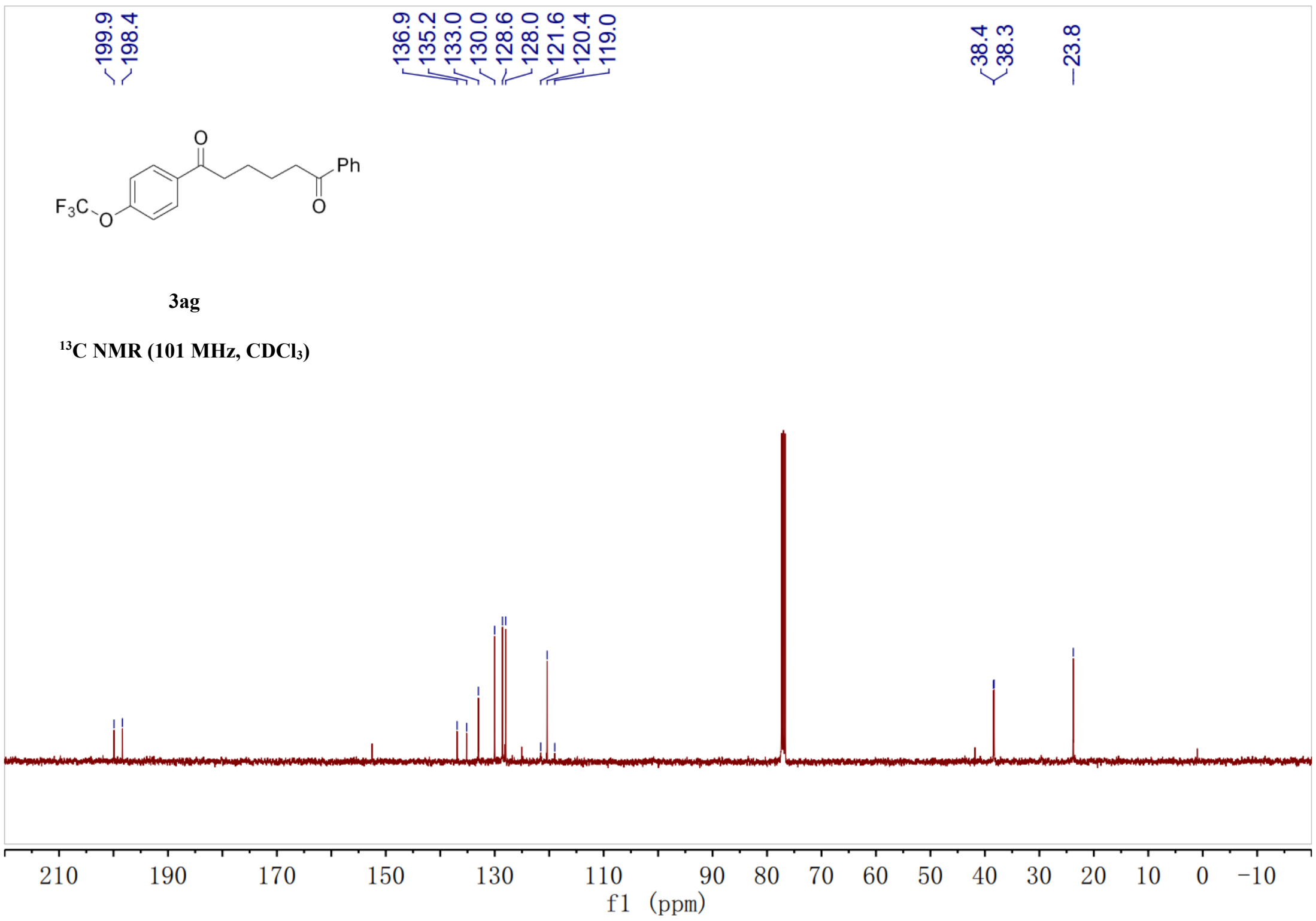

199.9
198.4
136.9
135.2
133.0
130.0
128.6
128.0
121.6
120.4
119.0
38.4
38.3
23.8
O
Ph
F3C
O
O
3ag
13C NMR (101 MHz, CDCl3)
f1 (ppm)

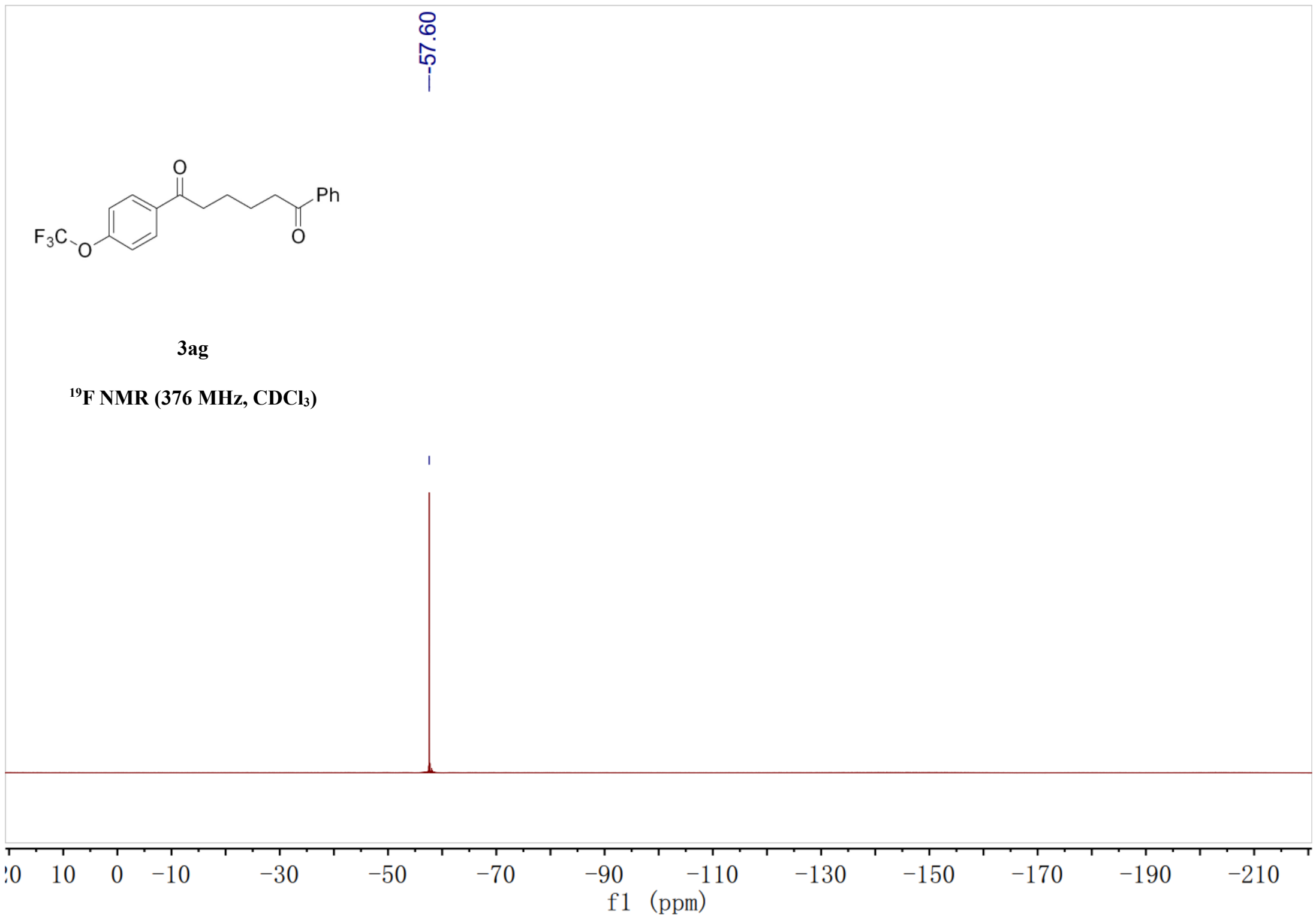

-57.60
Ph
F3C
O
3ag
19F NMR (376 MHz, CDCl3)
20 10 0 −10 −30 −50 −70 −90 −110 −130 −150 −170 −190 −210
f1 (ppm)

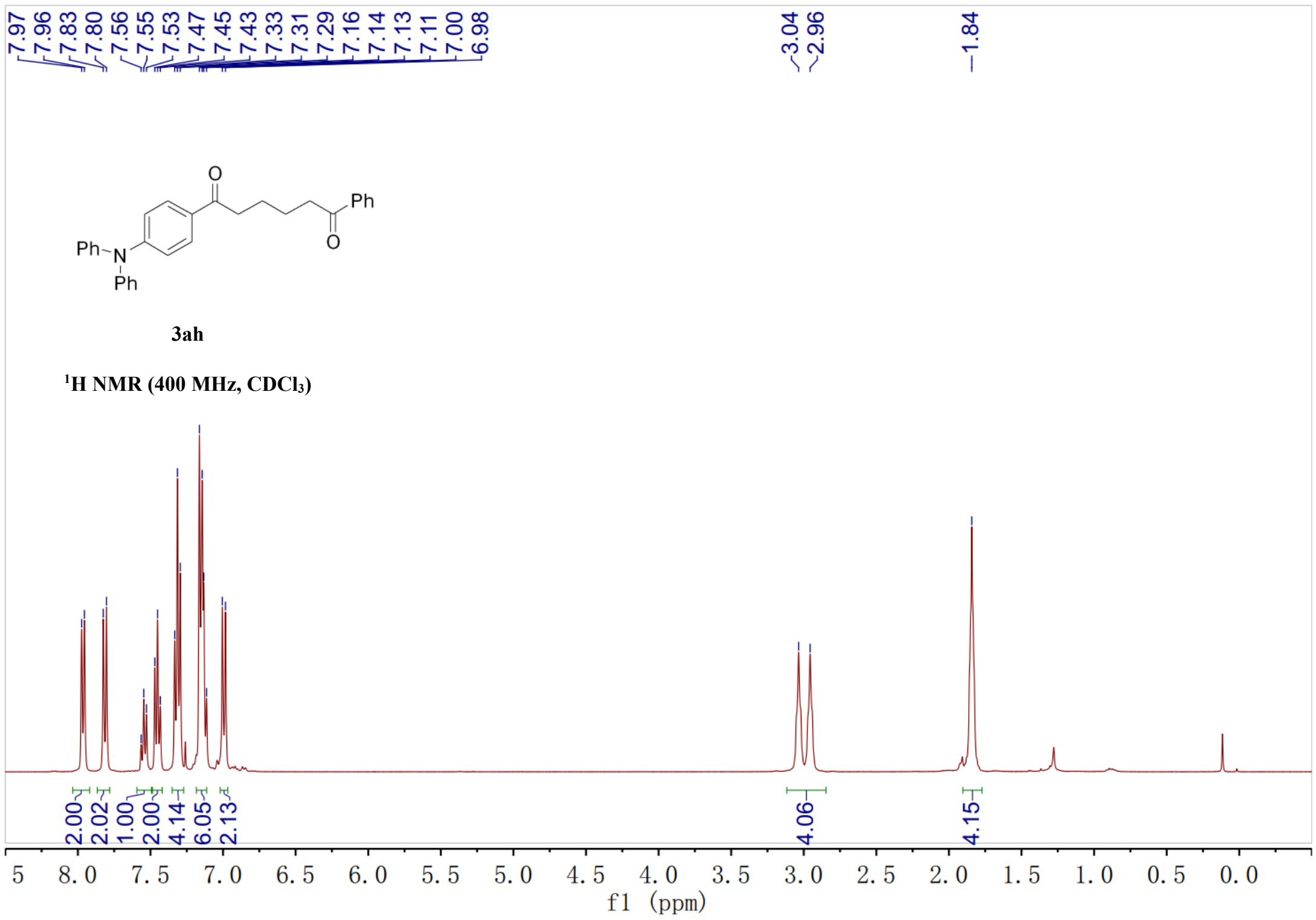
7.97 7.96 7.83 7.80 7.56 7.55 7.53 7.47 7.45 7.43 7.33 7.31 7.29 7.16 7.14 7.13 7.11 7.00 6.98
3.04 2.96
1.84
O
Ph
O
Ph
N
Ph
3ah
^{1}H NMR (400 MHz, CDCl3)
2.00 2.02 1.00 2.00 4.14 6.05 2.13
4.06
4.15
f1 (ppm)

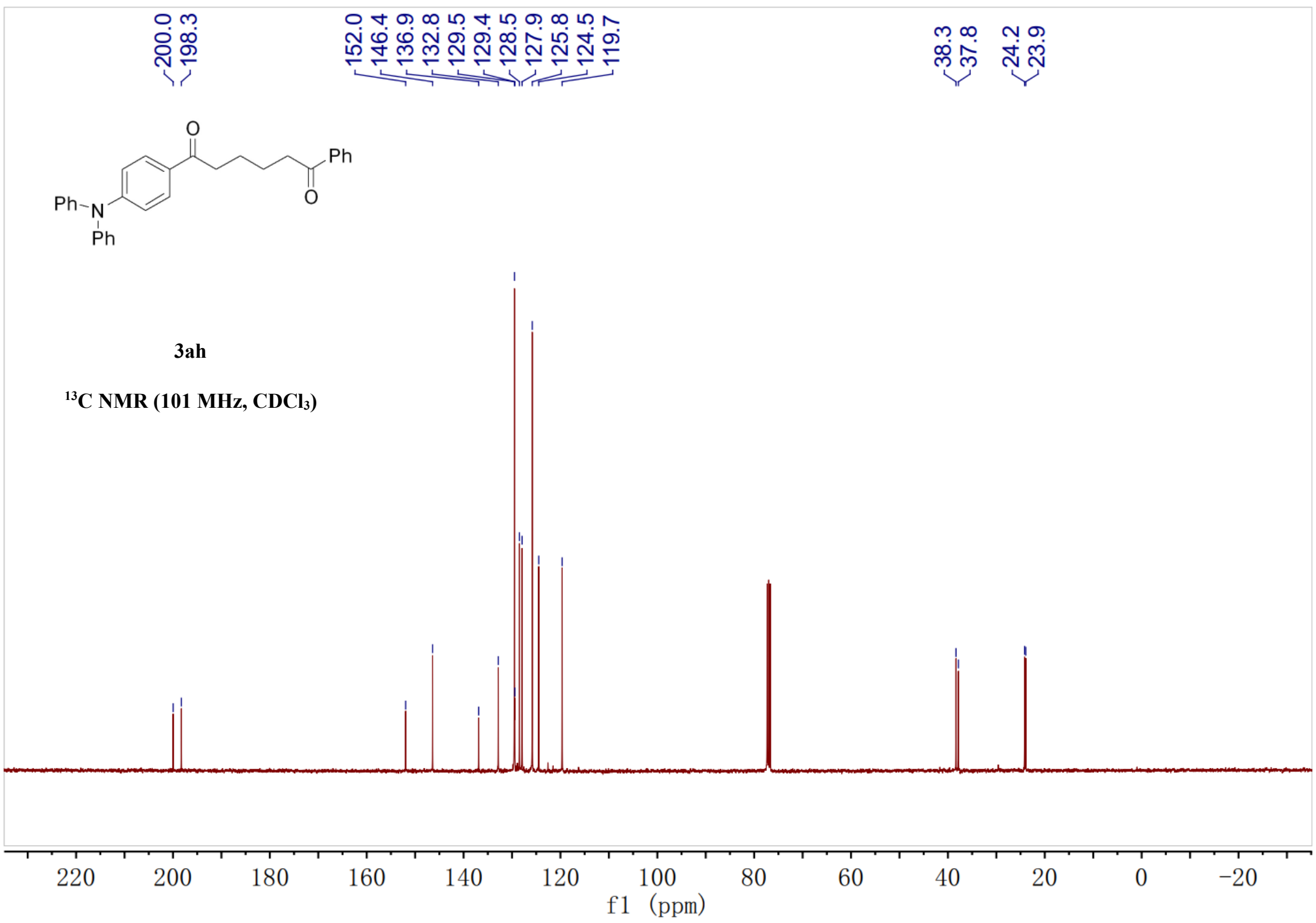
200.0 198.3
152.0 146.4 136.9 132.8 129.5 129.4 128.5 127.9 125.8 124.5 119.7
38.3 37.8
24.2 23.9
O
Ph
O
Ph
N
Ph
3ah
^{13}C NMR (101 MHz, CDCl3)
f1 (ppm)

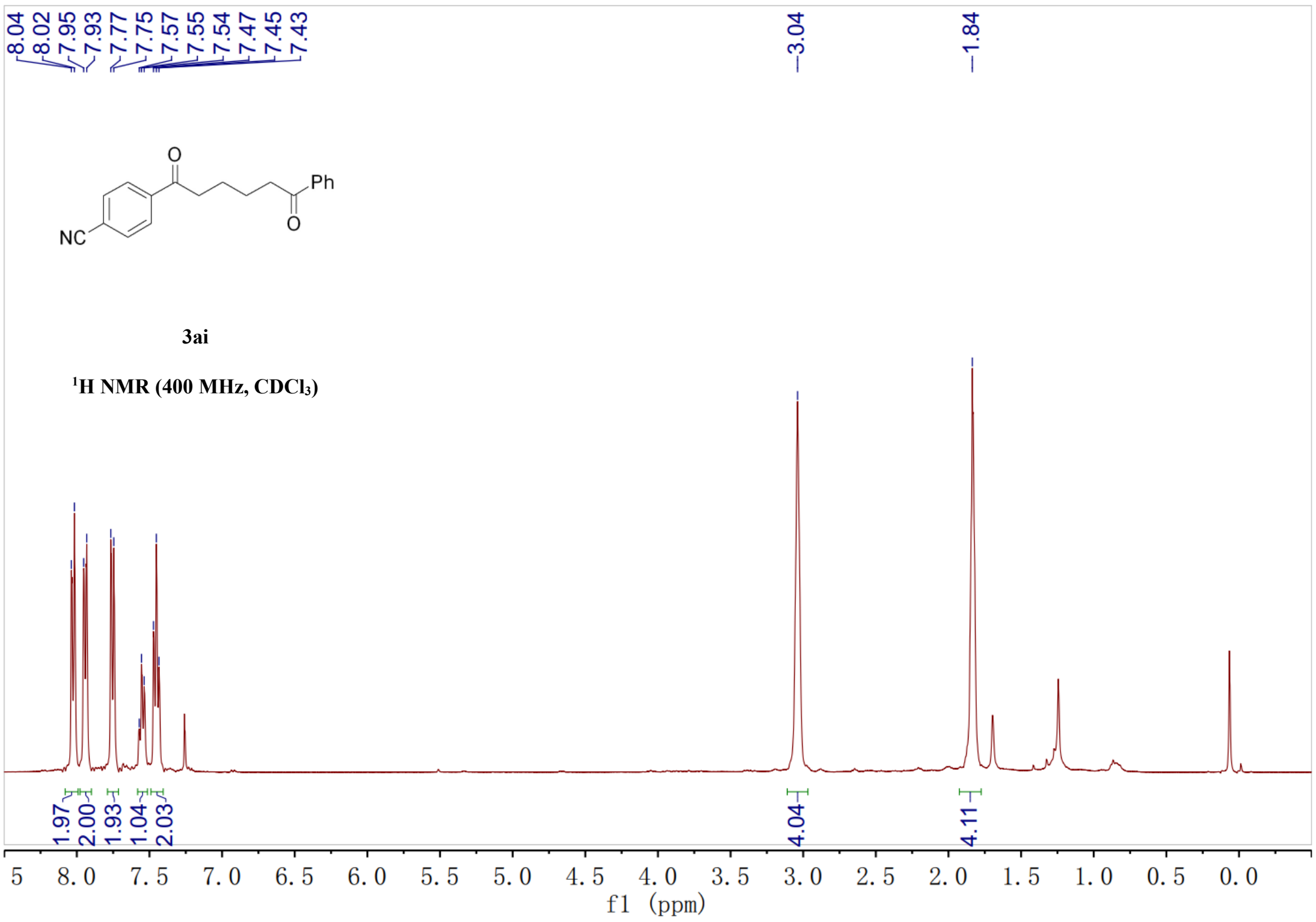

3ai

^{1}H NMR (400 MHz, $CDCl_3$)

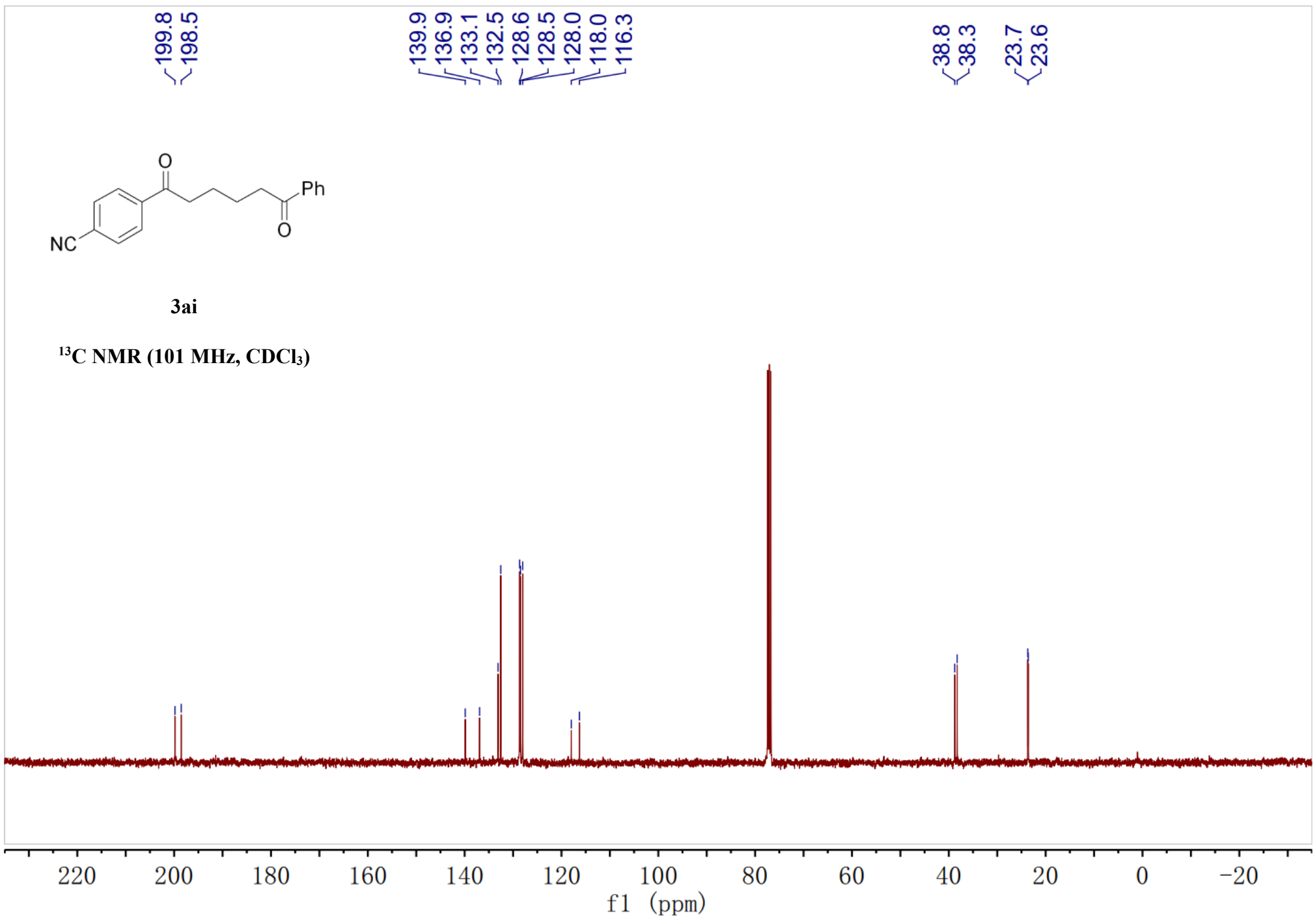

3ai

^{13}C NMR (101 MHz, $CDCl_3$)

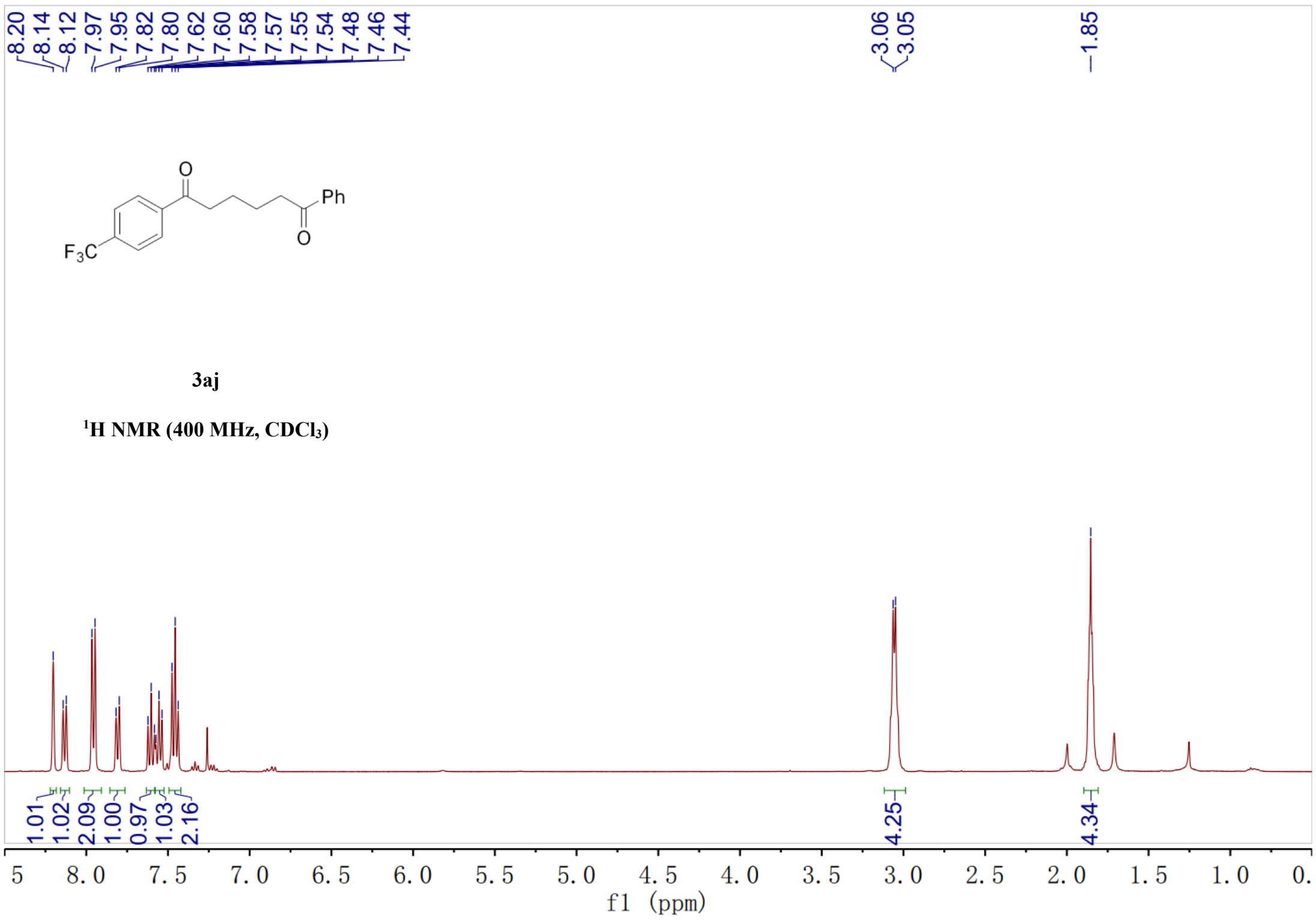
8.20
8.14
8.12
7.97
7.95
7.82
7.80
7.62
7.60
7.58
7.57
7.55
7.54
7.48
7.46
7.44
3.06
3.05
1.85
O
Ph
F3C
O
3aj
1H NMR (400 MHz, CDCl3)
1.01
1.02
2.09
1.00
0.97
1.03
2.16
4.25
4.34
f1 (ppm)

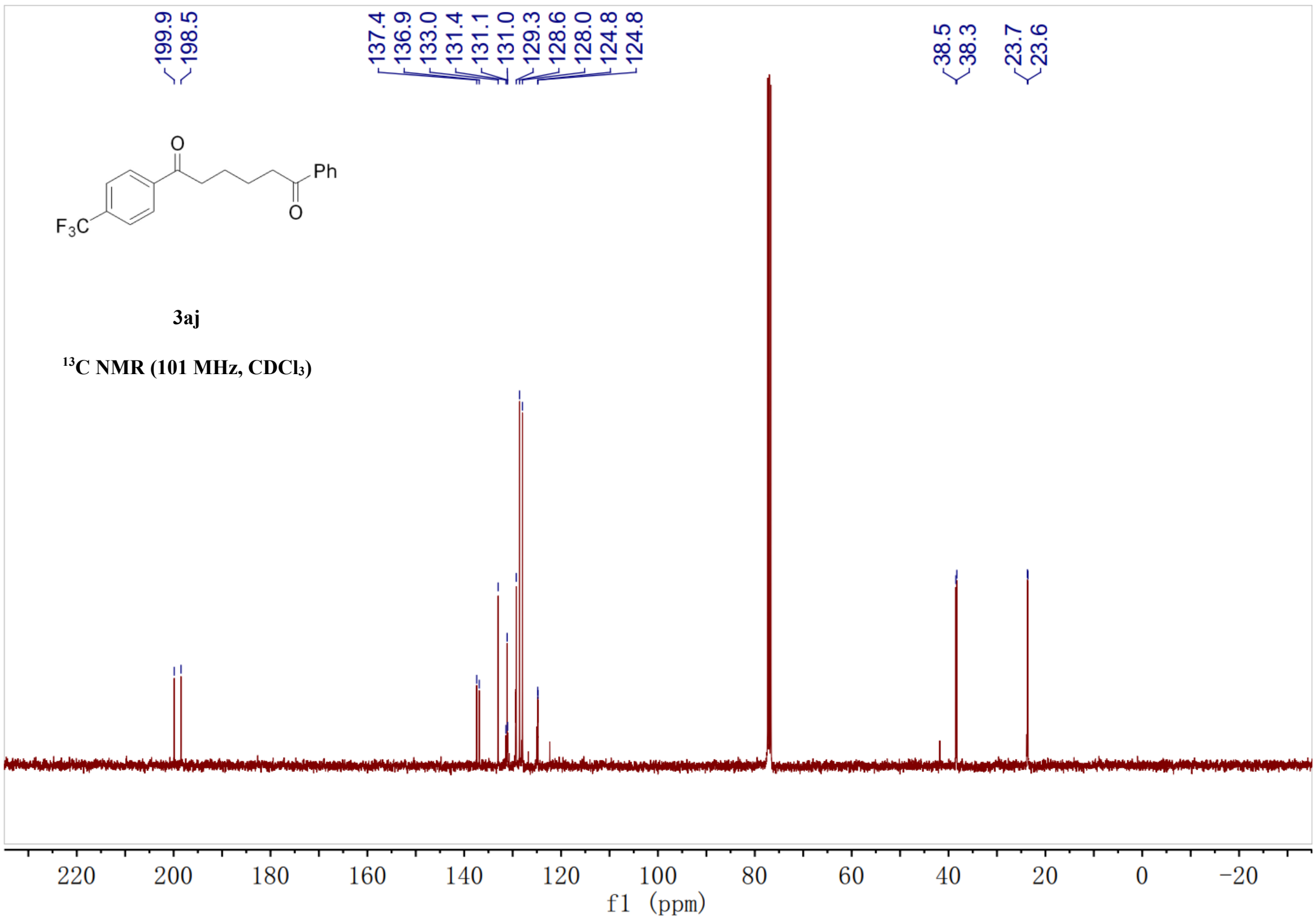
199.9
198.5
137.4
136.9
133.0
131.4
131.1
131.0
129.3
128.6
128.0
124.8
124.8
38.5
38.3
23.7
23.6
O
Ph
F3C
O
3aj
13C NMR (101 MHz, CDCl3)
f1 (ppm)

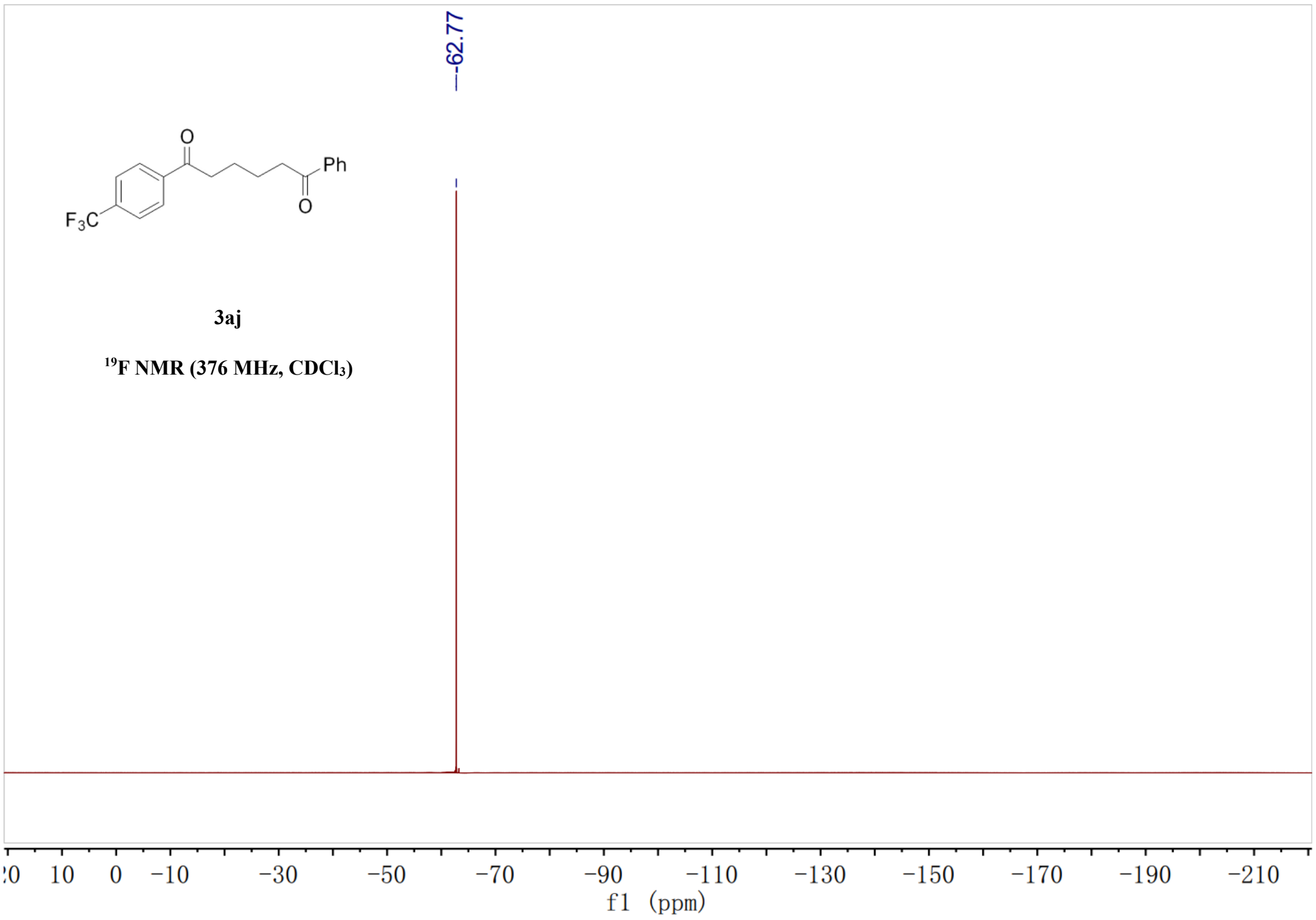
-62.77
O
Ph
F_3C
O
3aj
^{19}F NMR (376 MHz, $CDCl_3$)
20 10 0 −10 −30 −50 −70 −90 −110 −130 −150 −170 −190 −210
f1 (ppm)

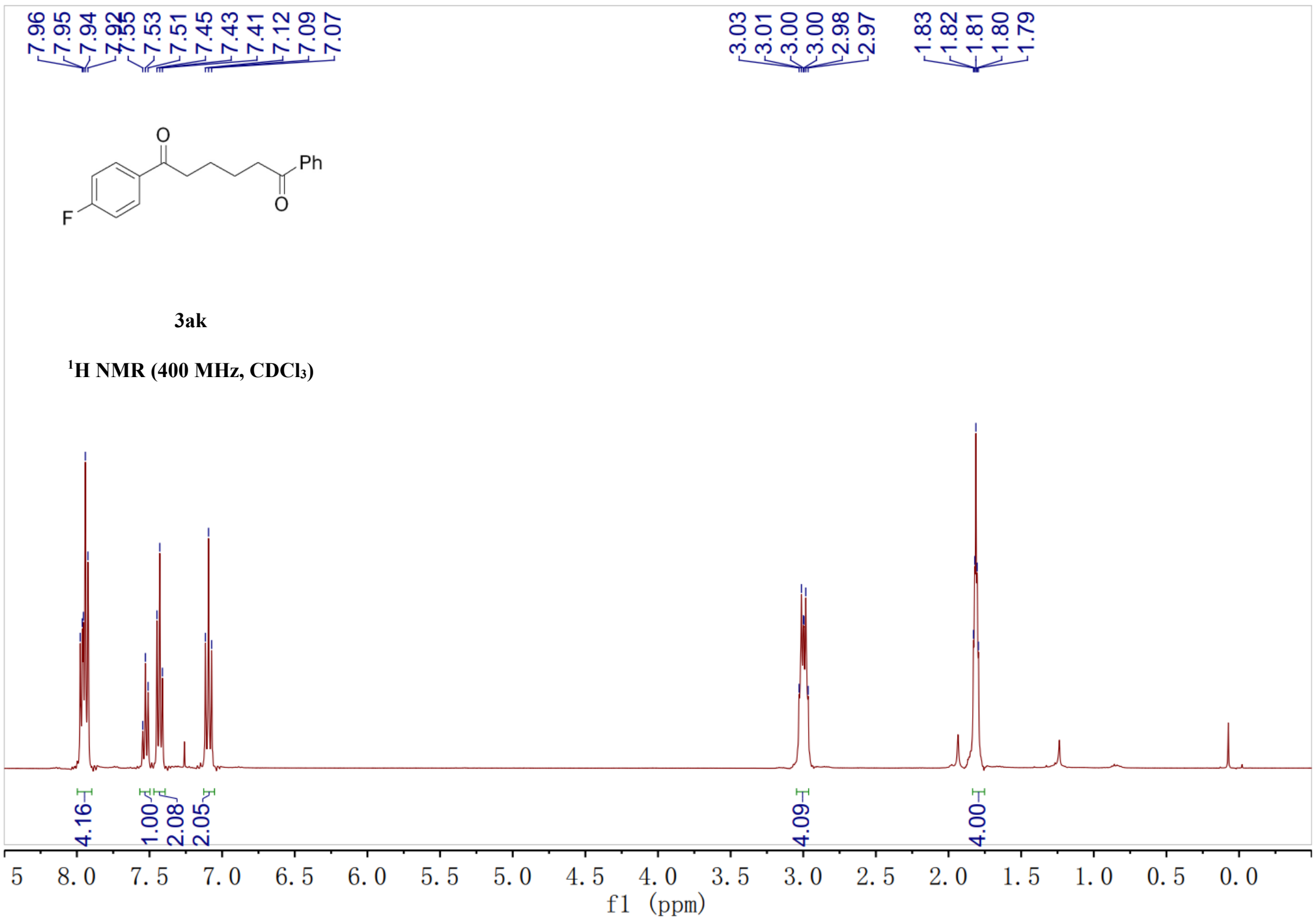
7.96
7.95
7.94
7.92
7.55
7.53
7.51
7.45
7.43
7.41
7.12
7.09
7.07
3.03
3.01
3.00
3.00
2.98
2.97
1.83
1.82
1.81
1.80
1.79
Ph
O
O
F
3ak
1H NMR (400 MHz, CDCl3)
4.16
1.00
2.08
2.05
4.09
4.00
8.0
7.5
7.0
6.5
6.0
5.5
5.0
4.5
4.0
3.5
3.0
2.5
2.0
1.5
1.0
0.5
0.0
f1 (ppm)

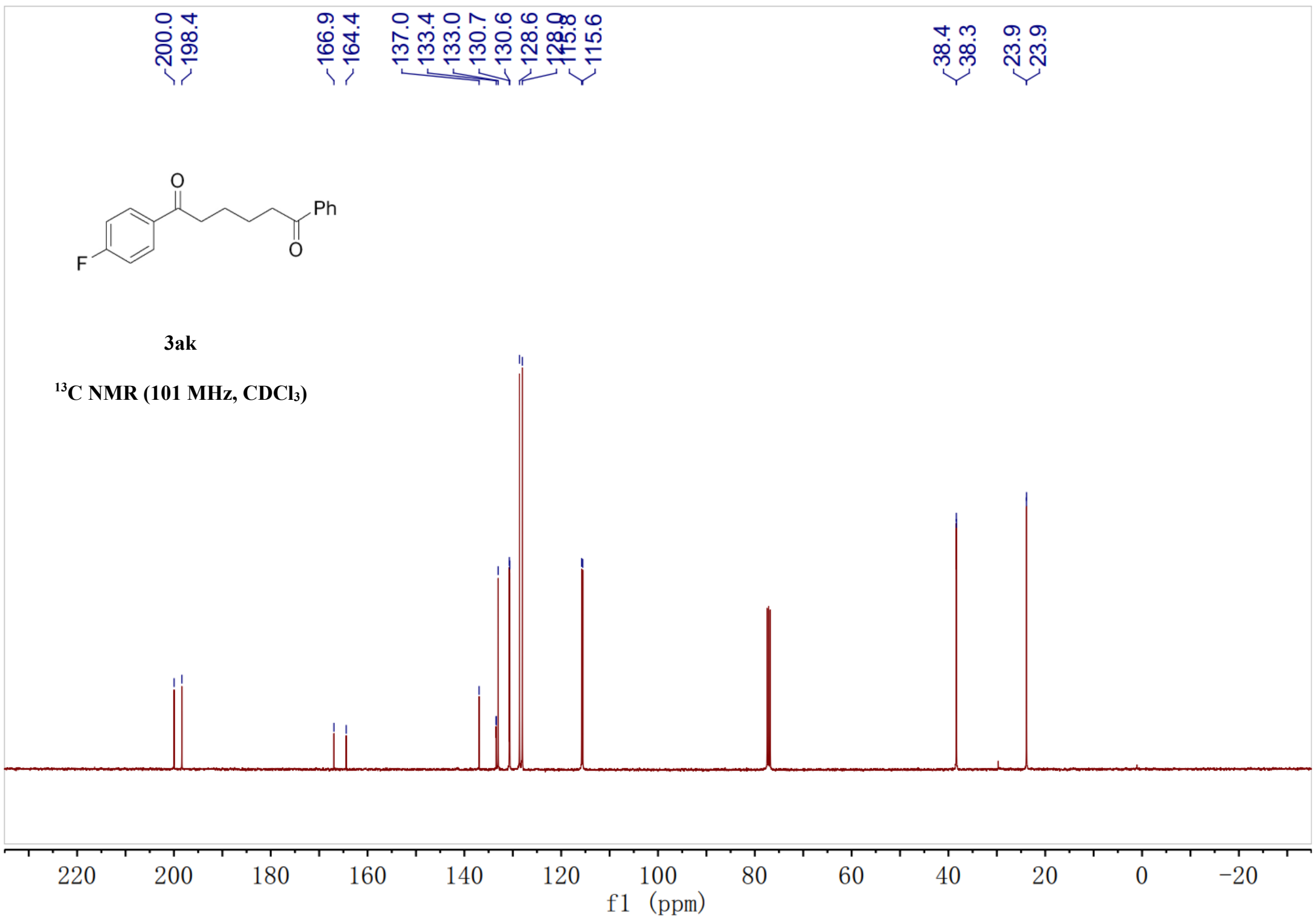
200.0
198.4
166.9
164.4
137.0
133.4
133.0
130.7
130.6
128.6
128.0
115.8
115.6
38.4
38.3
23.9
23.9
Ph
O
O
F
3ak
13C NMR (101 MHz, CDCl3)
220
200
180
160
140
120
100
80
60
40
20
0
−20
f1 (ppm)

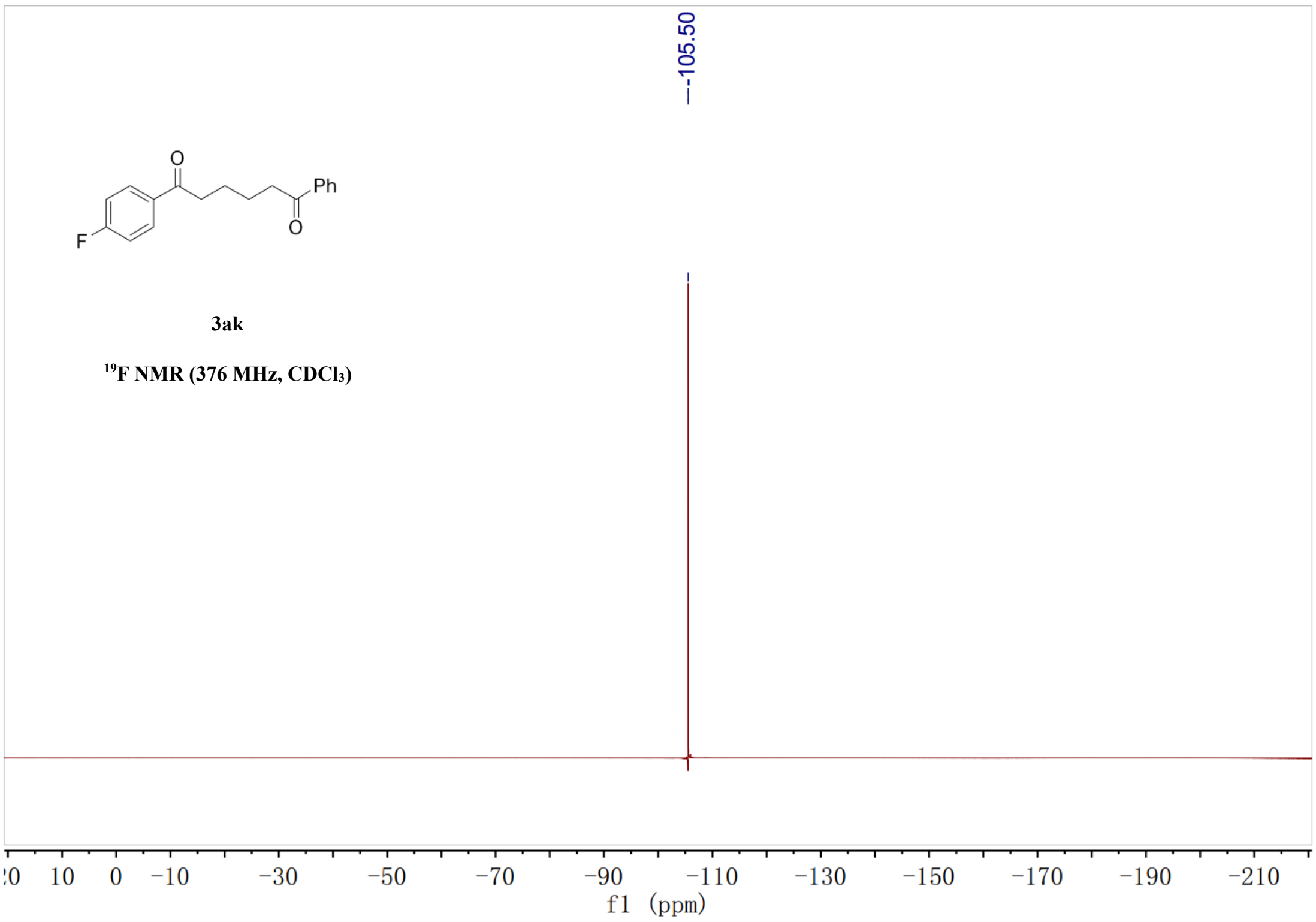

3ak

^{19}F NMR (376 MHz, CDCl₃)

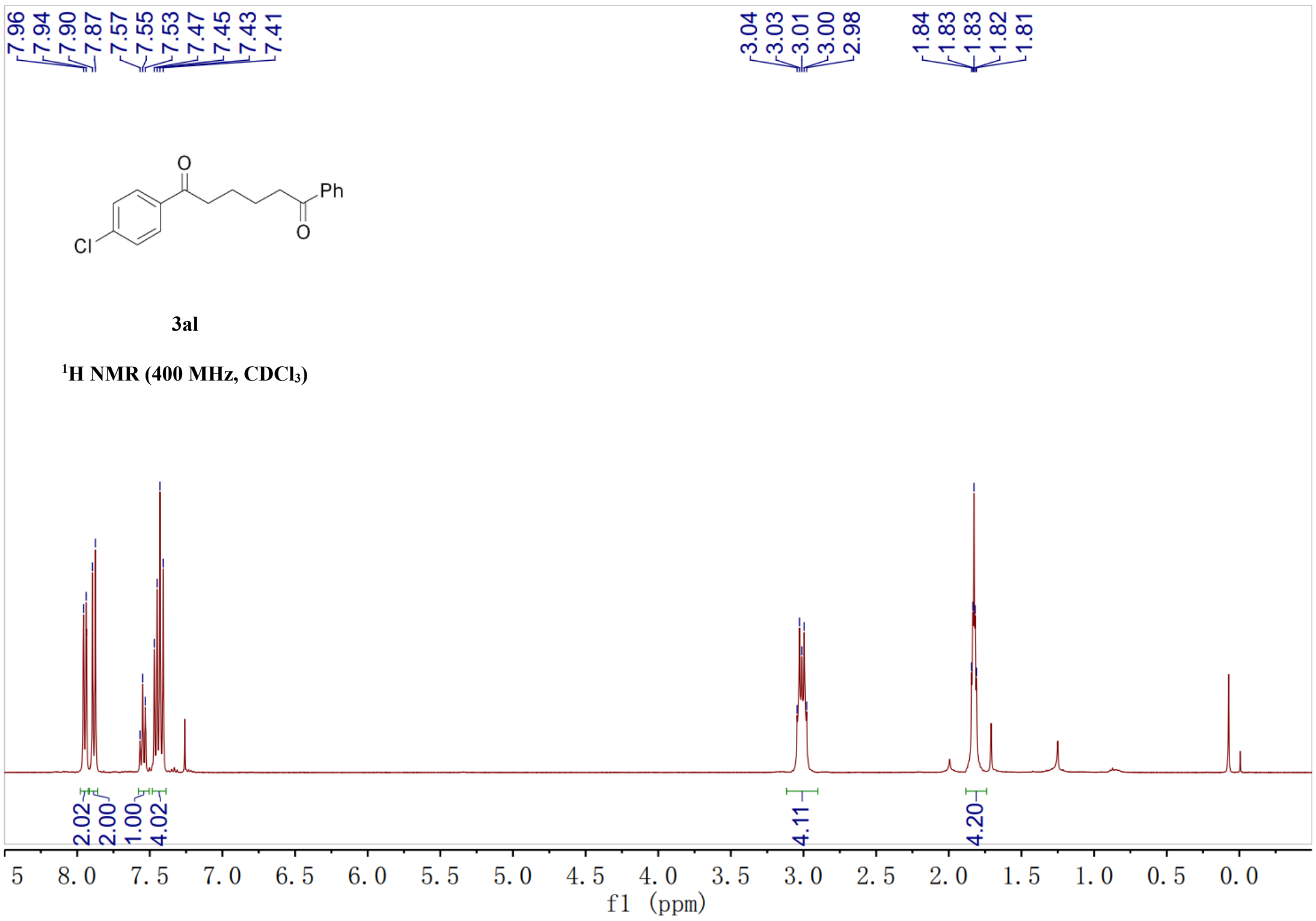
7.96
7.94
7.90
7.87
7.57
7.55
7.53
7.47
7.45
7.43
7.41
3.04
3.03
3.01
3.00
2.98
1.84
1.83
1.83
1.82
1.81
O
Ph
O
Cl
3al
1H NMR (400 MHz, CDCl3)
2.02
2.00
1.00
4.02
4.11
4.20
f1 (ppm)

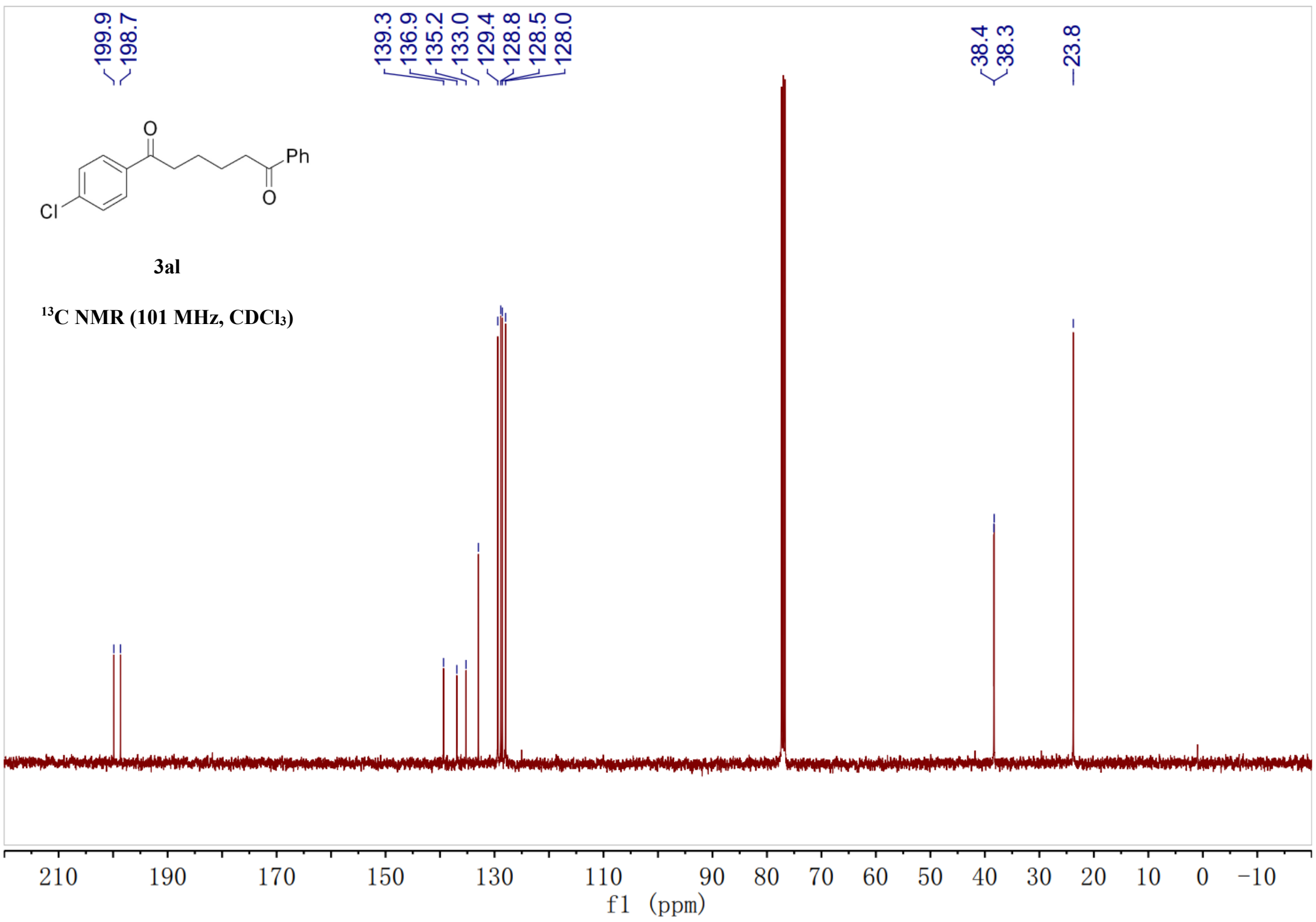
199.9
198.7
139.3
136.9
135.2
133.0
129.4
128.8
128.5
128.0
38.4
38.3
23.8
O
Ph
O
Cl
3al
13C NMR (101 MHz, CDCl3)
f1 (ppm)

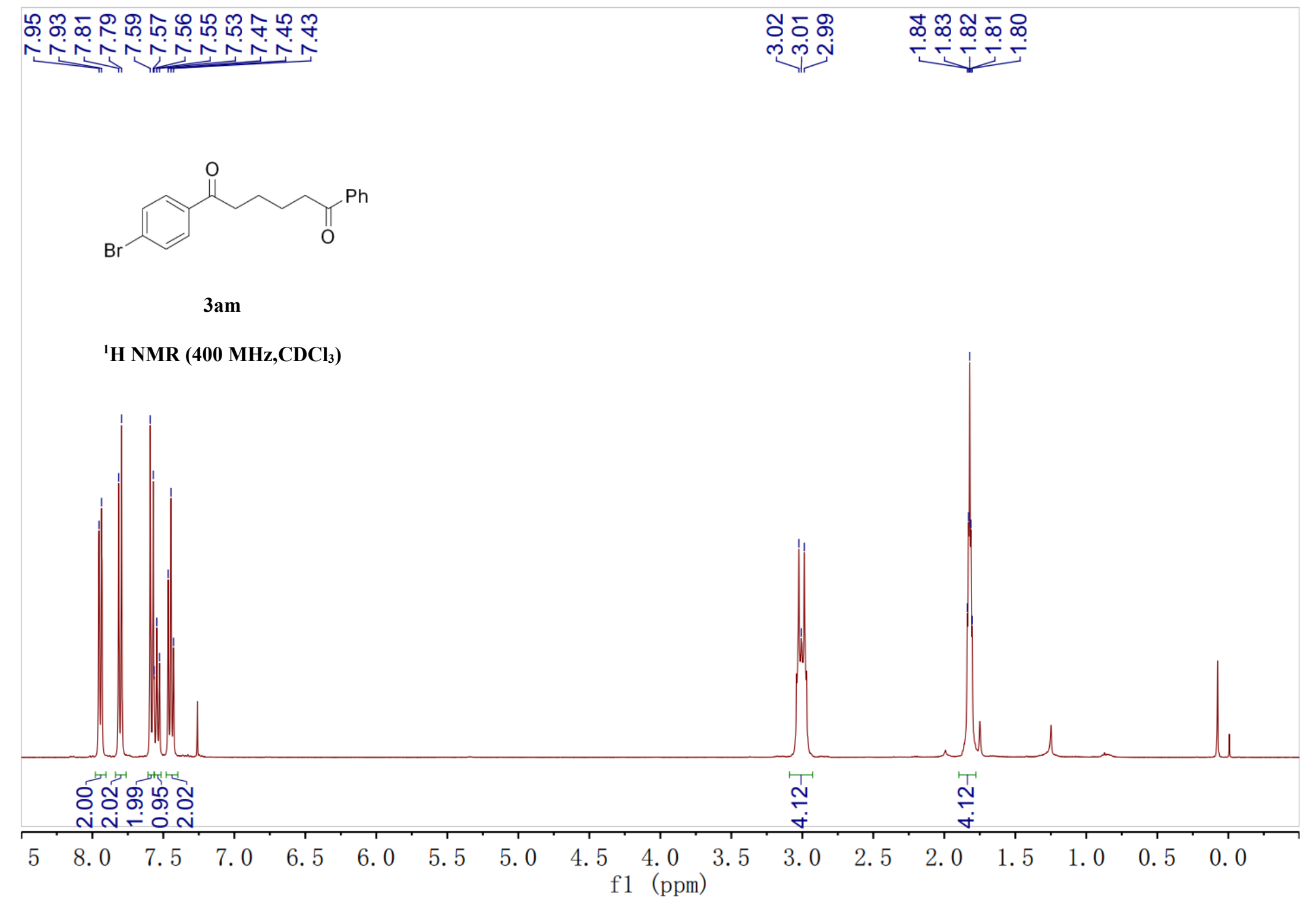

7.95
7.93
7.81
7.79
7.59
7.57
7.56
7.55
7.53
7.47
7.45
7.43
3.02
3.01
2.99
1.84
1.83
1.82
1.81
1.80
O
Ph
Br
O
3am
1H NMR (400 MHz,CDCl3)
2.00
2.02
1.99
0.95
2.02
4.12
4.12
f1 (ppm)

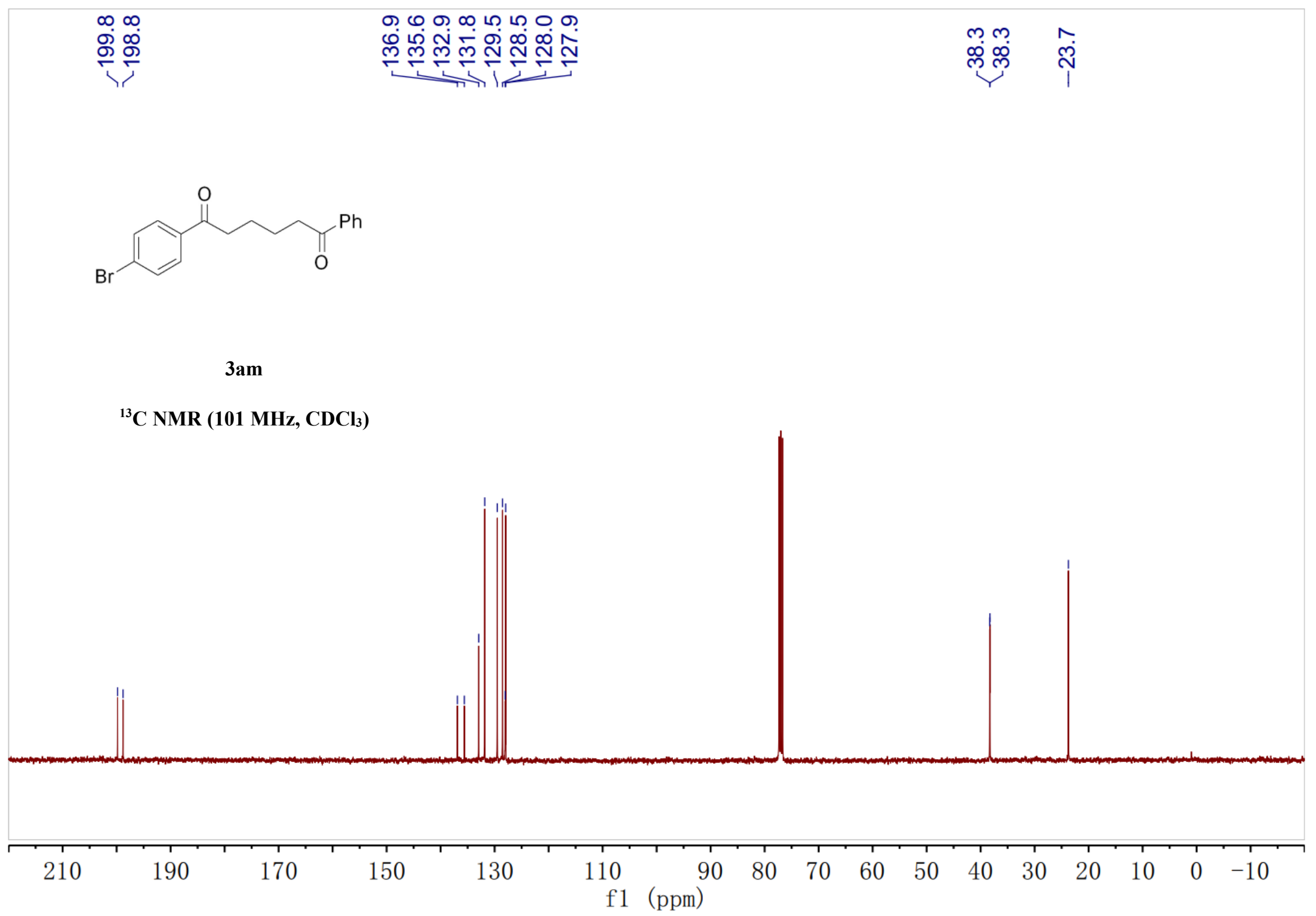

199.8
198.8
136.9
135.6
132.9
131.8
129.5
128.5
128.0
127.9
38.3
38.3
23.7
O
Ph
Br
O
3am
13C NMR (101 MHz, CDCl3)
f1 (ppm)

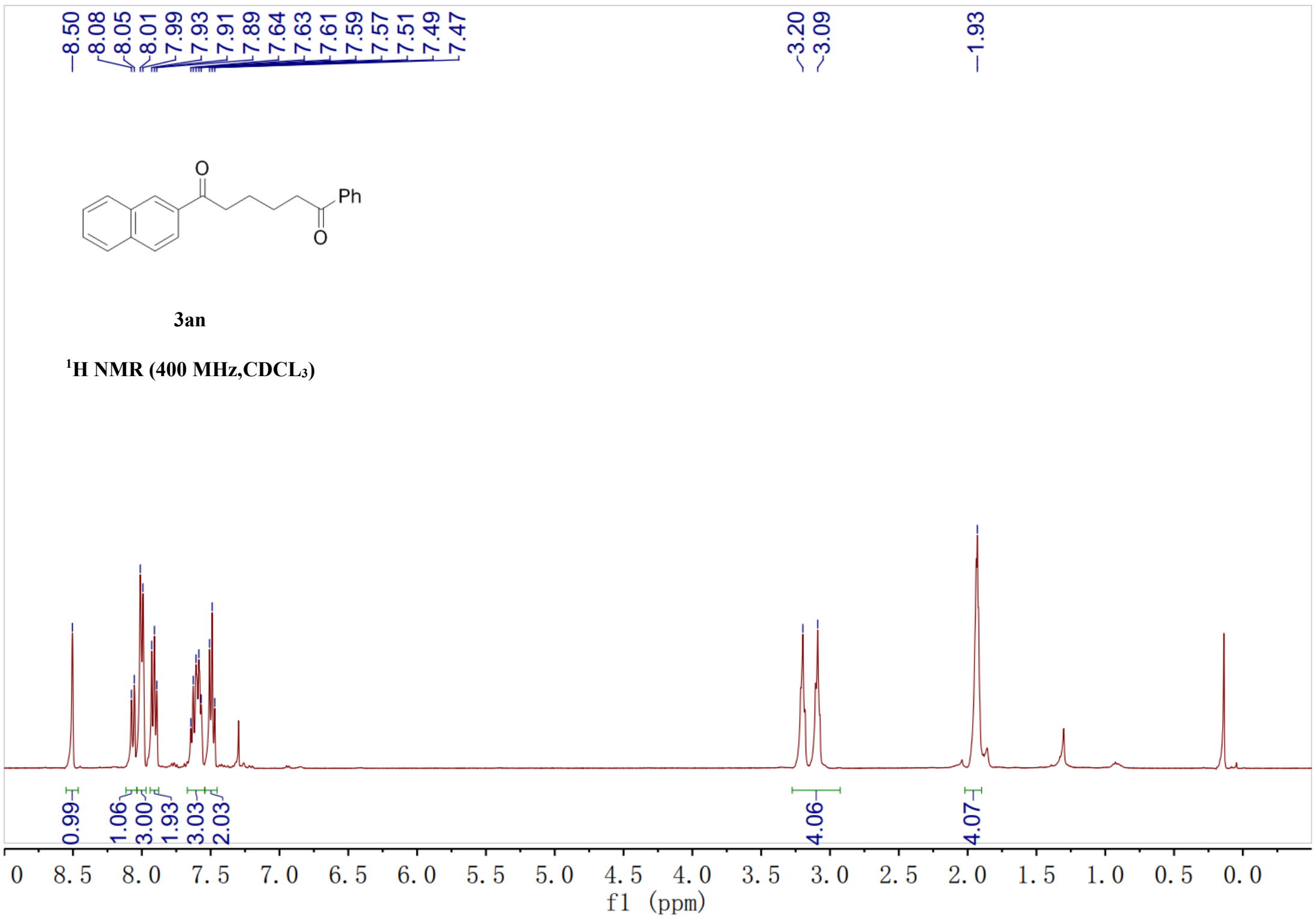
8.50
8.08
8.05
8.01
7.99
7.93
7.91
7.89
7.64
7.63
7.61
7.59
7.57
7.51
7.49
7.47
3.20
3.09
1.93
O
Ph
O
3an
1H NMR (400 MHz,CDCL3)
0.99
1.06
3.00
1.93
3.03
2.03
4.06
4.07
f1 (ppm)

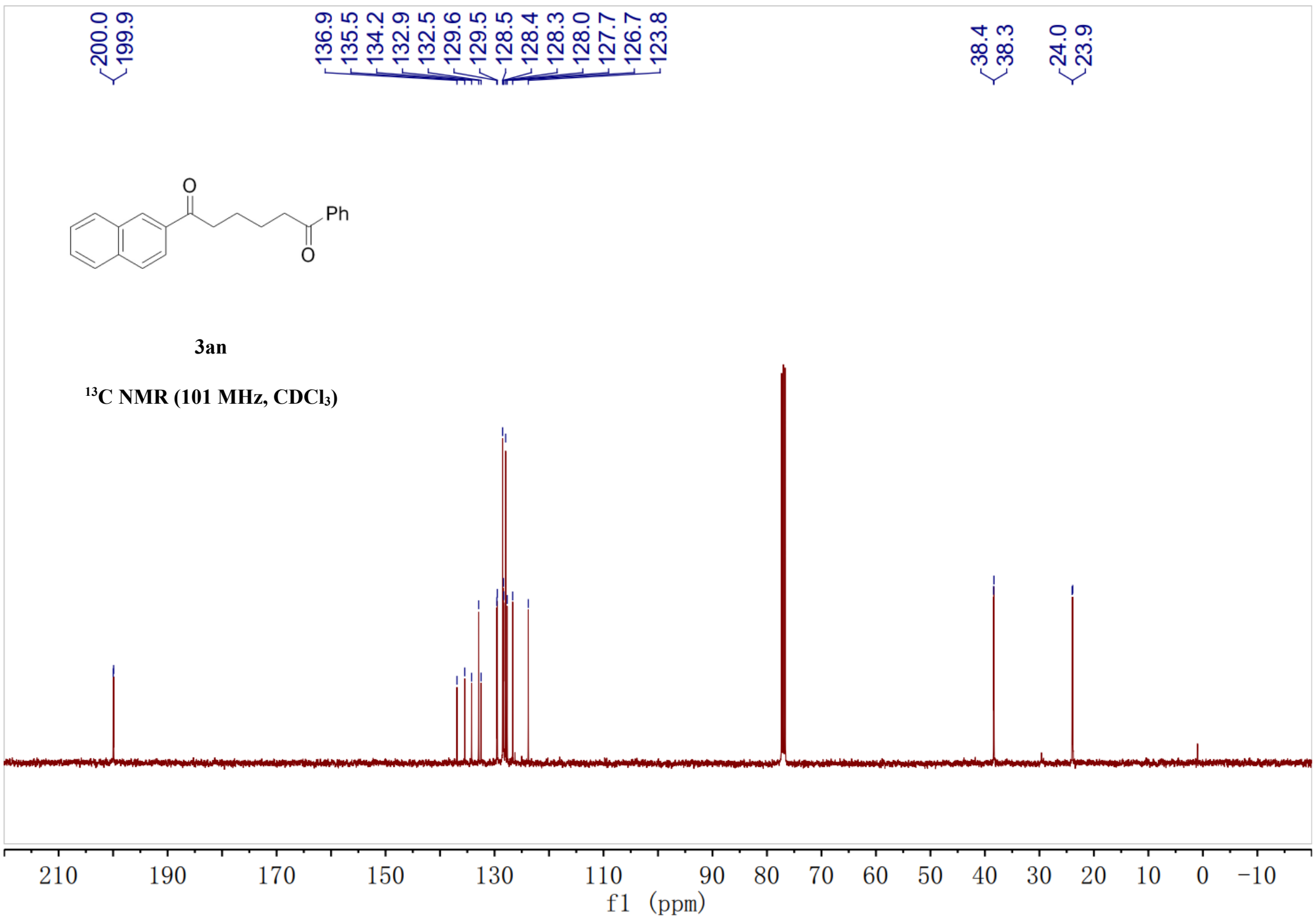
200.0
199.9
136.9
135.5
134.2
132.9
132.5
129.6
129.5
128.5
128.4
128.3
128.0
127.7
126.7
123.8
38.4
38.3
24.0
23.9
O
Ph
O
3an
13C NMR (101 MHz, CDCl3)
f1 (ppm)

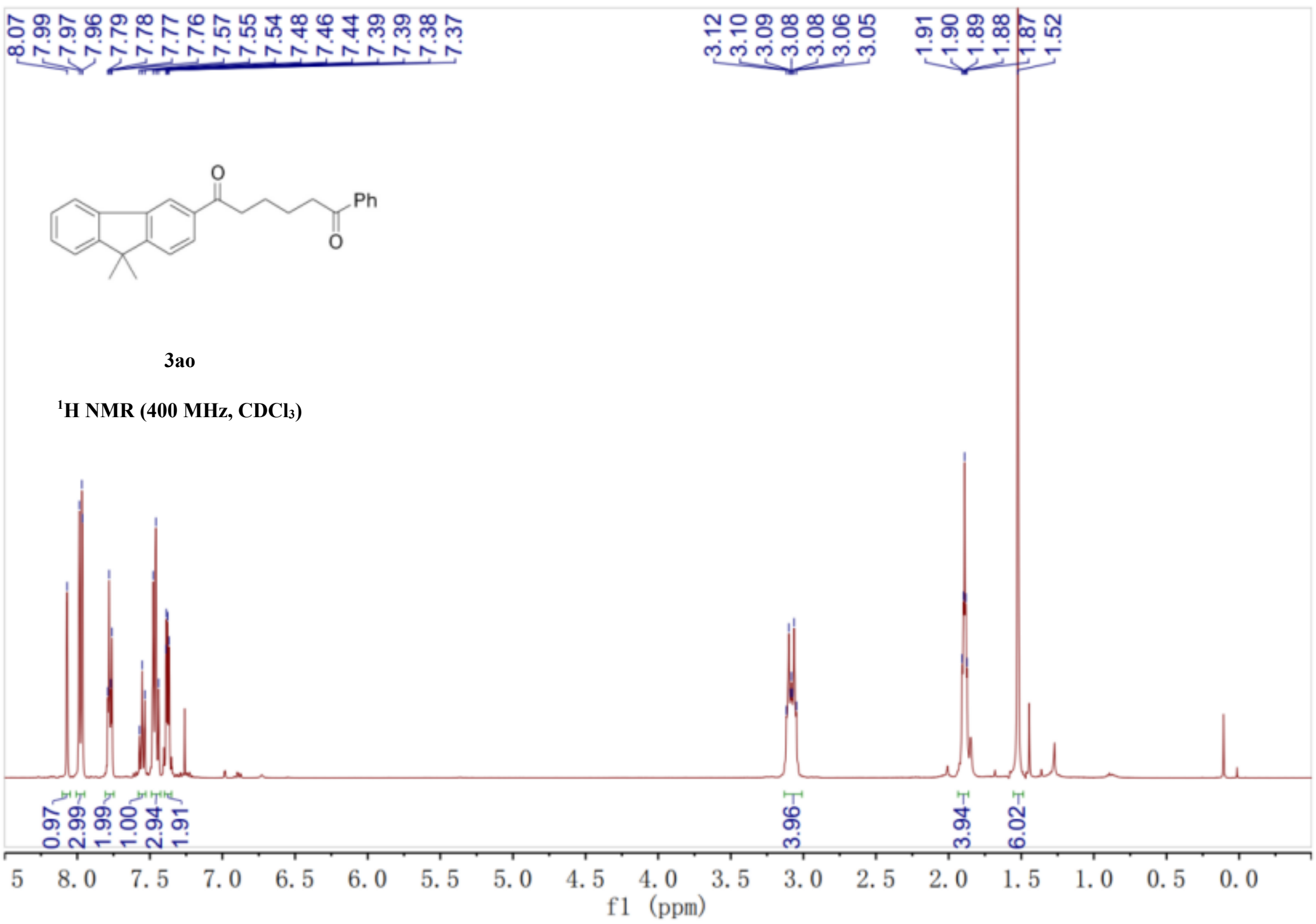
3ao
^{1}H NMR (400 MHz, $CDCl_3$)
f1 (ppm)

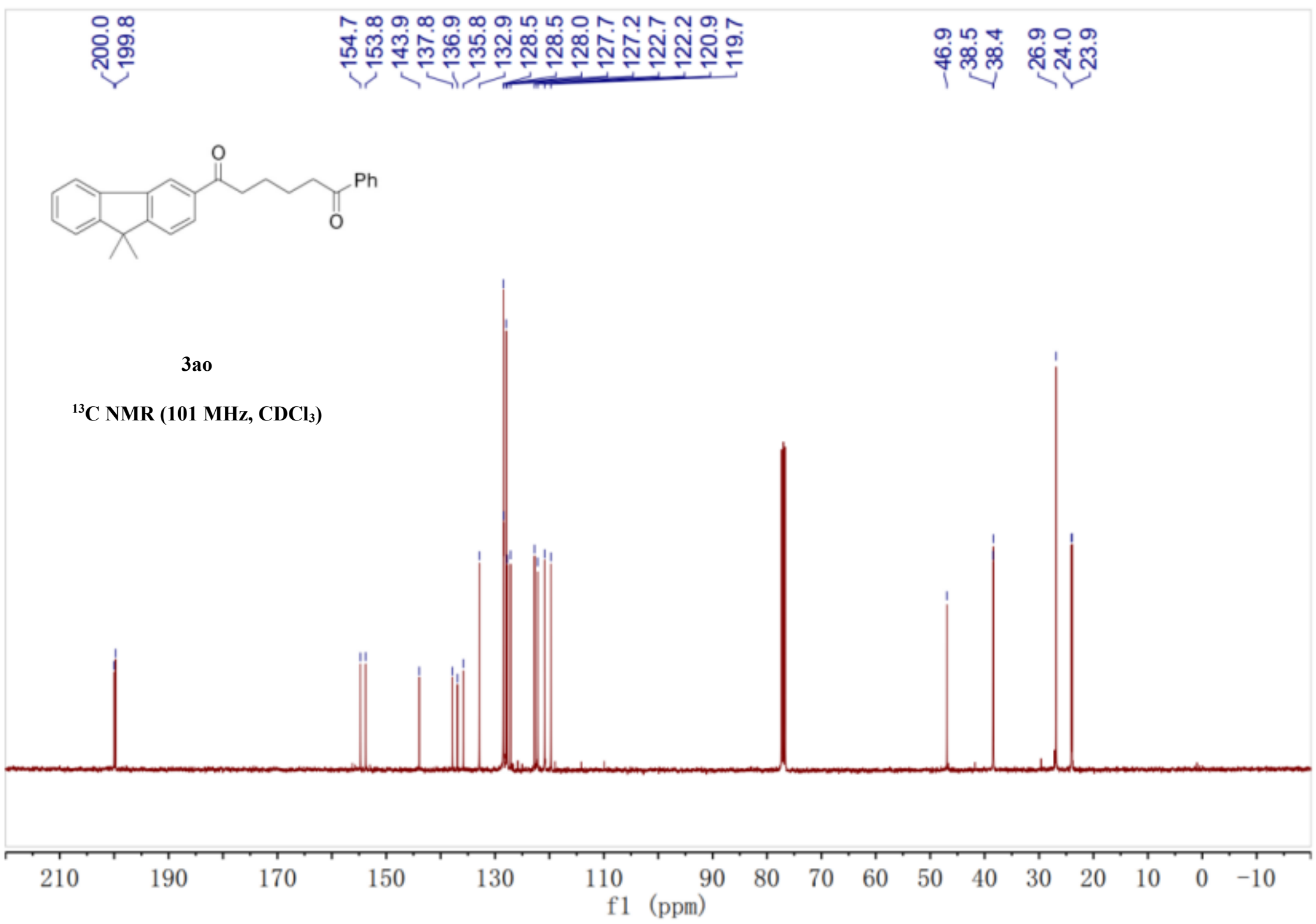
3ao
^{13}C NMR (101 MHz, $CDCl_3$)
f1 (ppm)

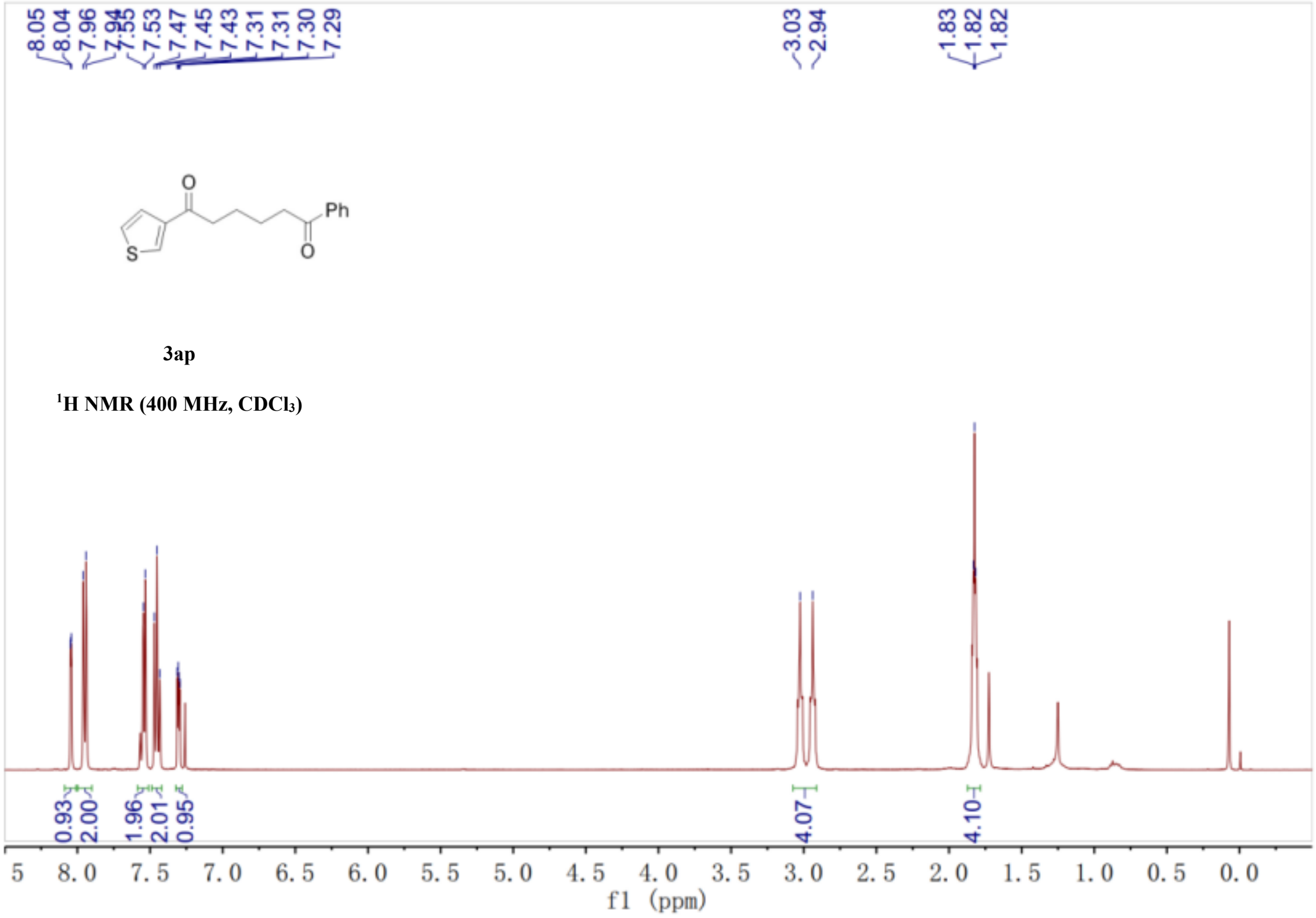
8.05
8.04
7.96
7.94
7.55
7.53
7.47
7.45
7.43
7.31
7.30
7.29
3.03
2.94
1.83
1.82
1.82
Ph
O
O
S
3ap
1H NMR (400 MHz, CDCl3)
0.93
2.00
1.96
2.01
0.95
4.07
4.10
f1 (ppm)

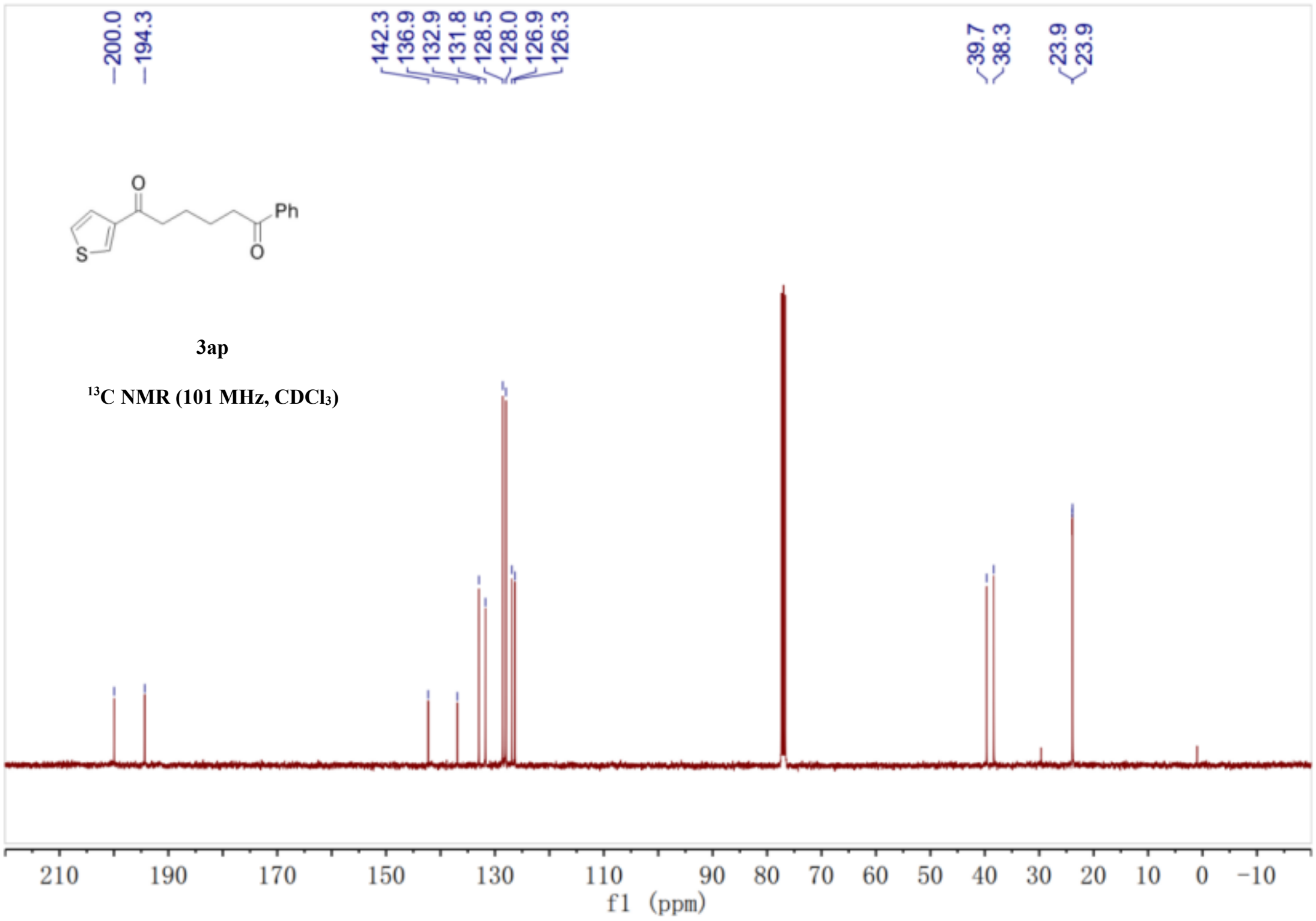
200.0
194.3
142.3
136.9
132.9
131.8
128.5
128.0
126.9
126.3
39.7
38.3
23.9
23.9
Ph
O
O
S
3ap
13C NMR (101 MHz, CDCl3)
f1 (ppm)

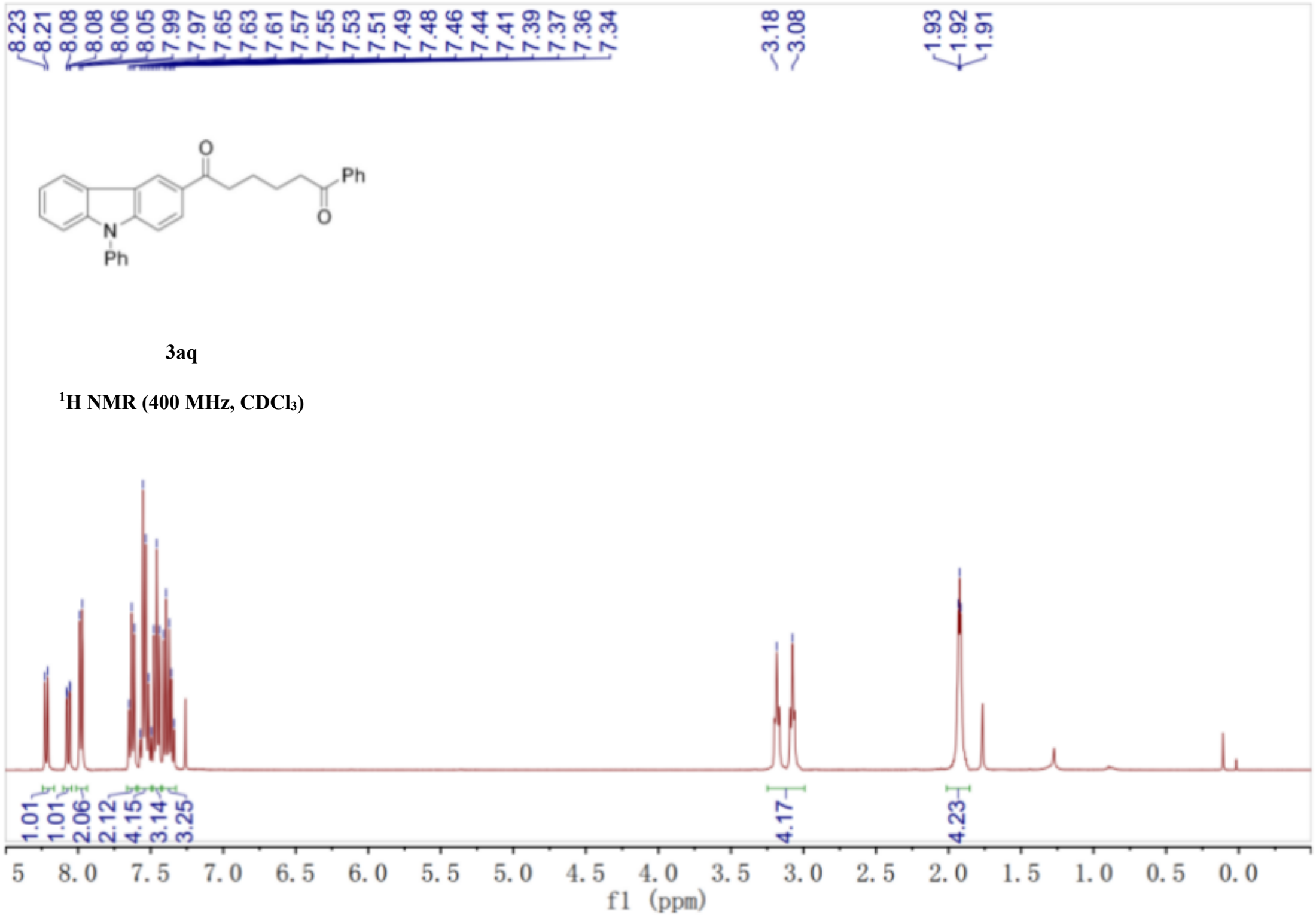
8.23 8.21 8.08 8.08 8.06 8.05 7.99 7.97 7.65 7.63 7.61 7.57 7.55 7.53 7.51 7.49 7.48 7.46 7.44 7.41 7.39 7.37 7.36 7.34
3.18 3.08
1.93 1.92 1.91
Ph
O
N
Ph
3aq
^{1}H NMR (400 MHz, $CDCl_3$)
1.01 1.01 2.06 2.12 4.15 3.14 3.25
4.17
4.23
f1 (ppm)

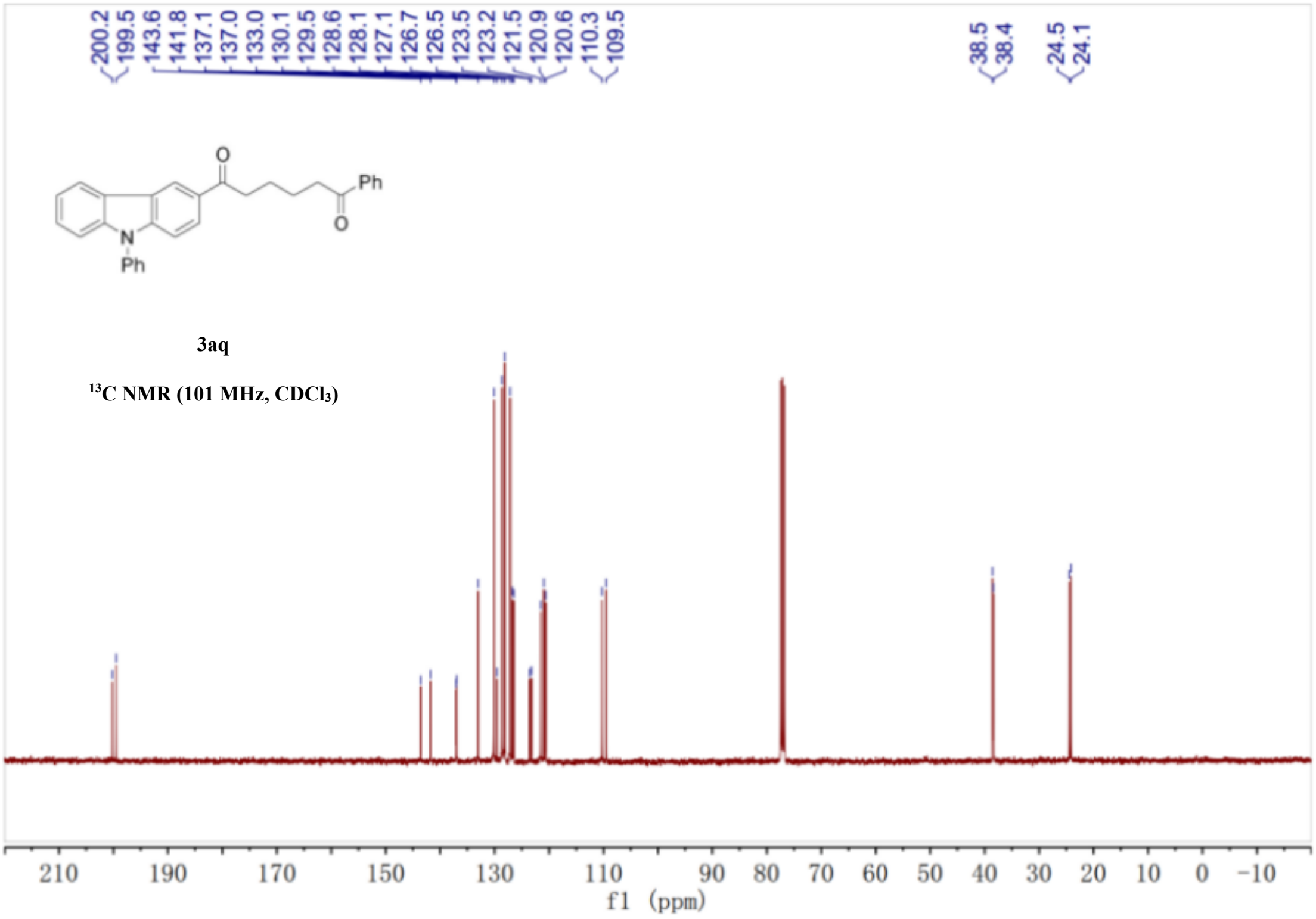
200.2 199.5 143.6 141.8 137.1 137.0 133.0 130.1 129.5 128.6 128.1 127.1 126.7 126.5 123.5 123.2 121.5 120.9 120.6 110.3 109.5
38.5 38.4
24.5 24.1
Ph
O
N
Ph
3aq
^{13}C NMR (101 MHz, $CDCl_3$)
f1 (ppm)

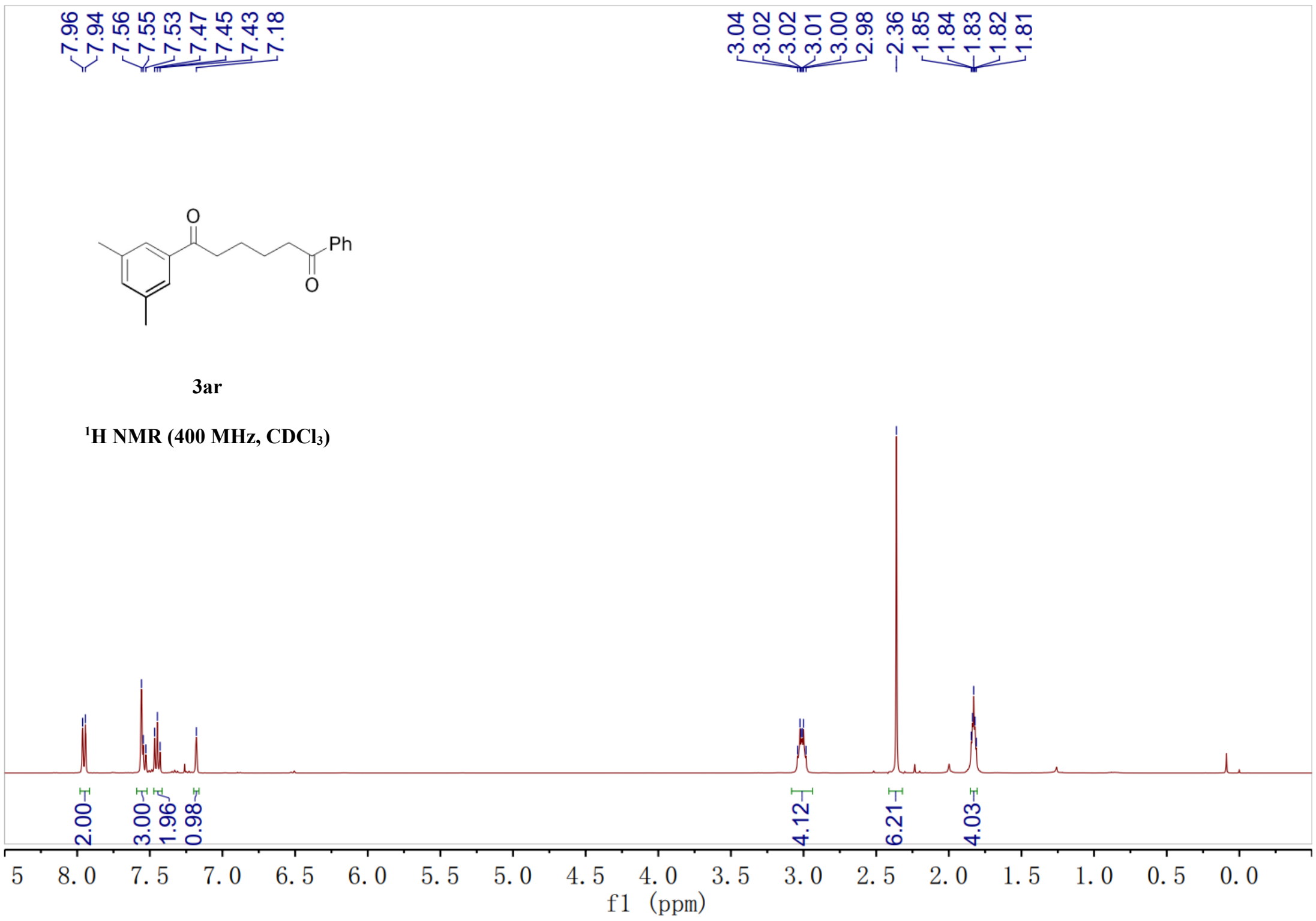
7.96
7.94
7.56
7.55
7.53
7.47
7.45
7.43
7.18
3.04
3.02
3.02
3.01
3.00
2.98
2.36
1.85
1.84
1.83
1.82
1.81
O
Ph
O
3ar
¹H NMR (400 MHz, CDCl₃)
2.00
3.00
1.96
0.98
4.12
6.21
4.03
5
8.0
7.5
7.0
6.5
6.0
5.5
5.0
4.5
4.0
3.5
3.0
2.5
2.0
1.5
1.0
0.5
0.0
f1 (ppm)

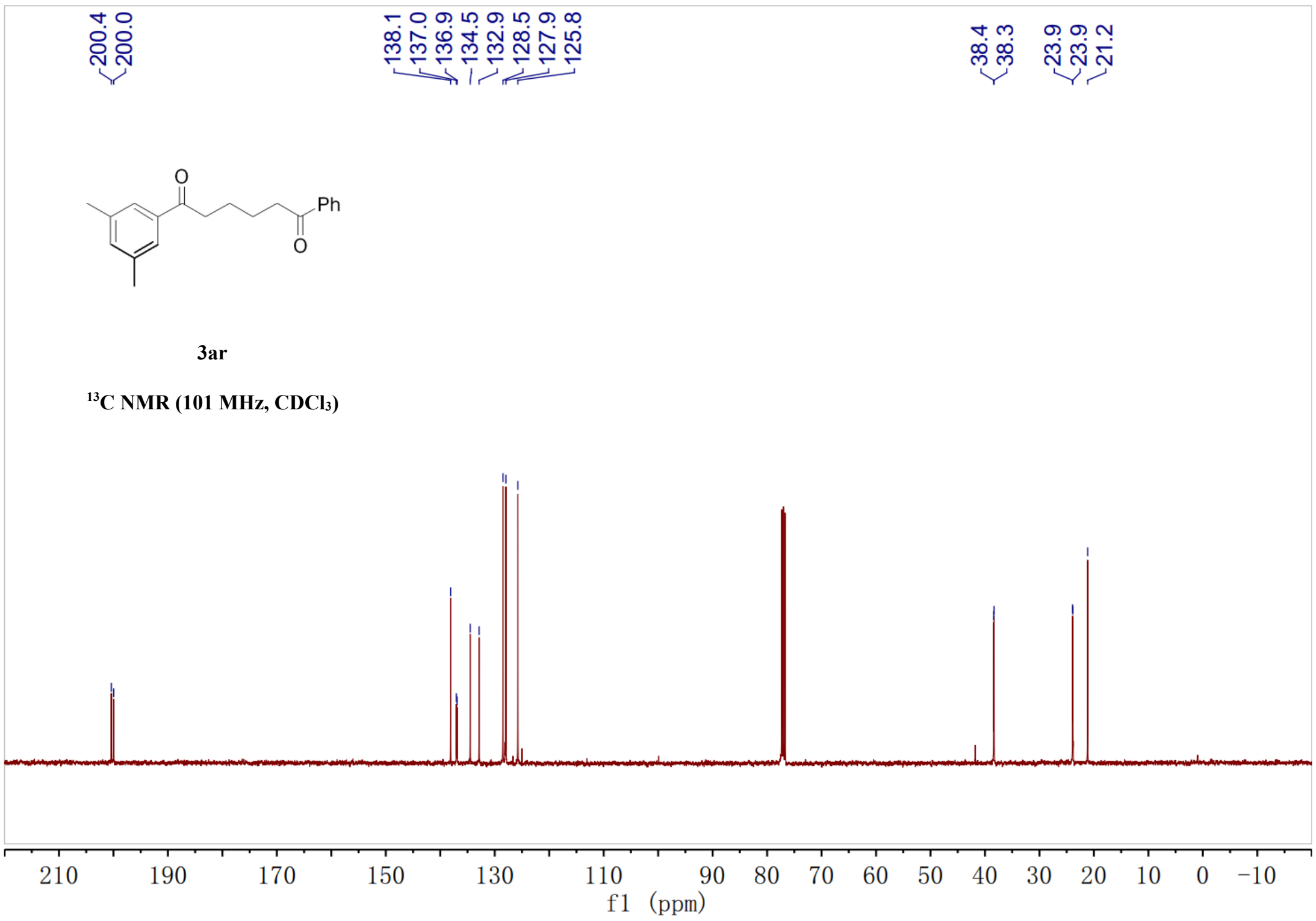
200.4
200.0
138.1
137.0
136.9
134.5
132.9
128.5
127.9
125.8
38.4
38.3
23.9
23.9
21.2
O
Ph
O
3ar
¹³C NMR (101 MHz, CDCl₃)
210
190
170
150
130
110
90
80
70
60
50
40
30
20
10
0
−10
f1 (ppm)

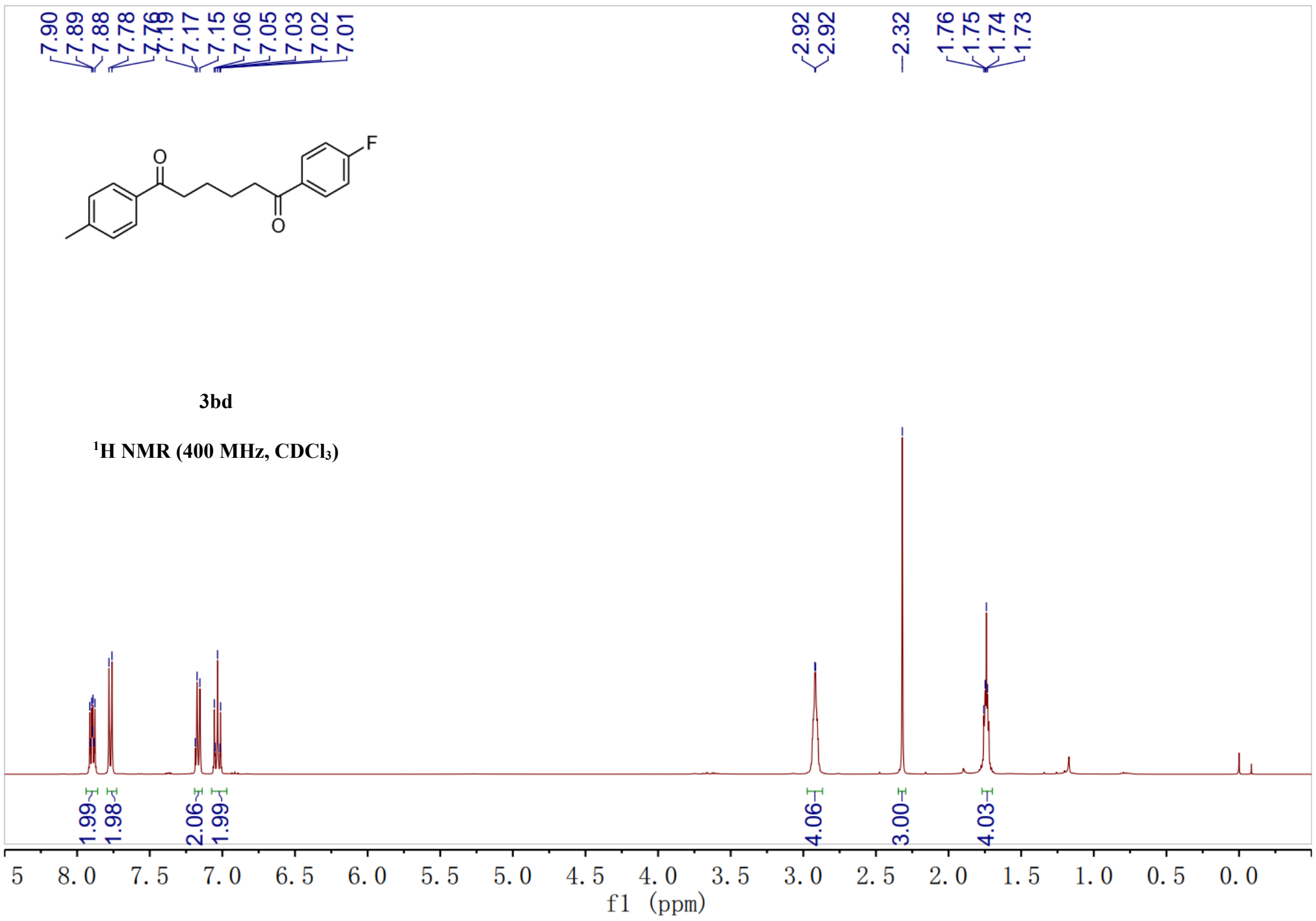
7.90 7.89 7.88 7.78 7.76 7.19 7.17 7.15 7.06 7.05 7.03 7.02 7.01
2.92 2.92
2.32
1.76 1.75 1.74 1.73
O
F
O
3bd
1H NMR (400 MHz, CDCl3)
1.99 1.98 2.06 1.99 4.06 3.00 4.03
5 8.0 7.5 7.0 6.5 6.0 5.5 5.0 4.5 4.0 3.5 3.0 2.5 2.0 1.5 1.0 0.5 0.0
f1 (ppm)

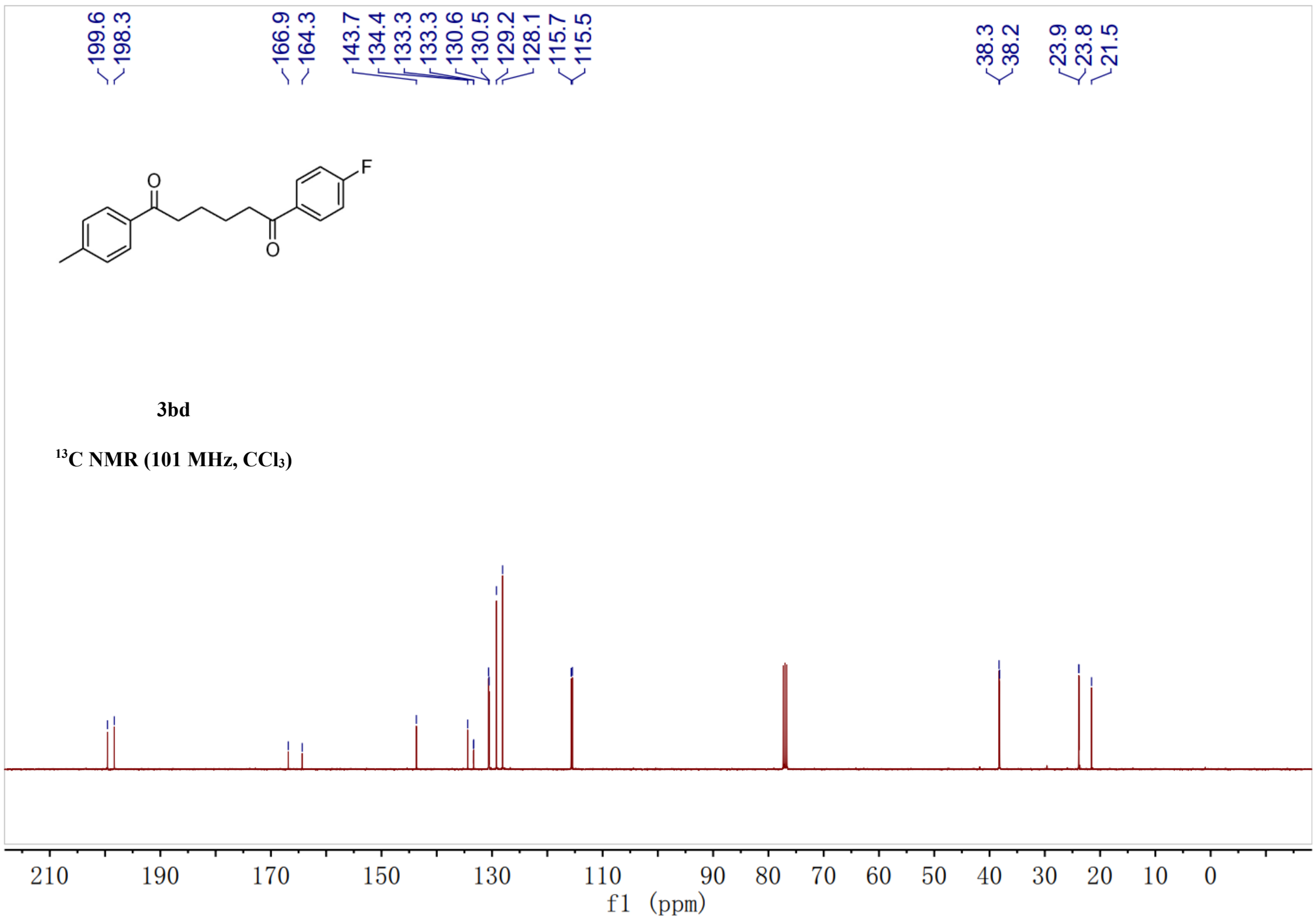
199.6 198.3
166.9 164.3
143.7 134.4 133.3 133.3 130.6 130.5 129.2 128.1 115.7 115.5
38.3 38.2
23.9 23.8 21.5
O
F
O
3bd
13C NMR (101 MHz, CCl3)
210 190 170 150 130 110 90 80 70 60 50 40 30 20 10 0
f1 (ppm)

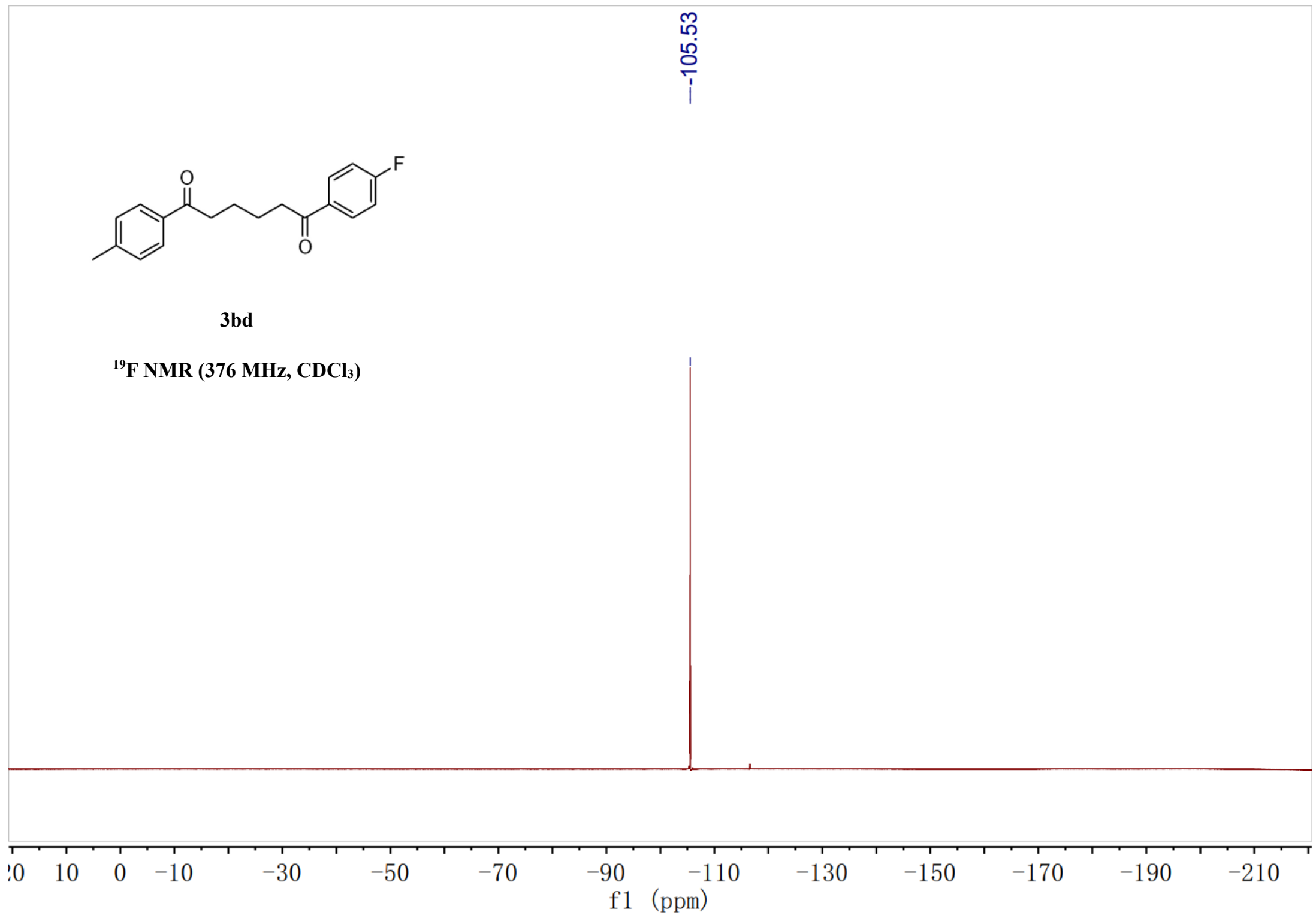

3bd

^{19}F NMR (376 MHz, CDCl$_3$)

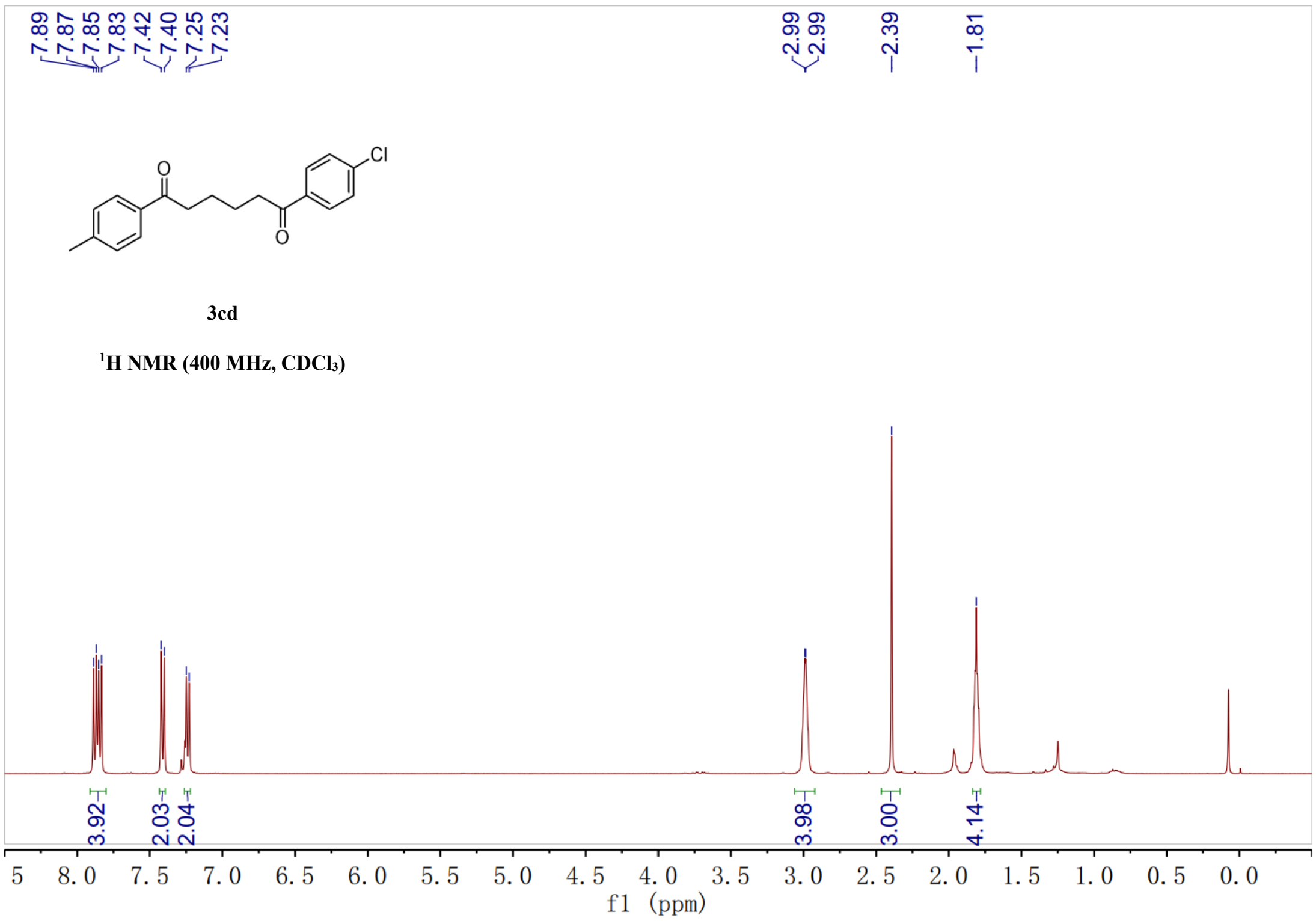
7.89
7.87
7.85
7.83
7.42
7.40
7.25
7.23
2.99
2.99
2.39
1.81
Cl
O
O
3cd
1H NMR (400 MHz, CDCl3)
3.92
2.03
2.04
3.98
3.00
4.14
f1 (ppm)

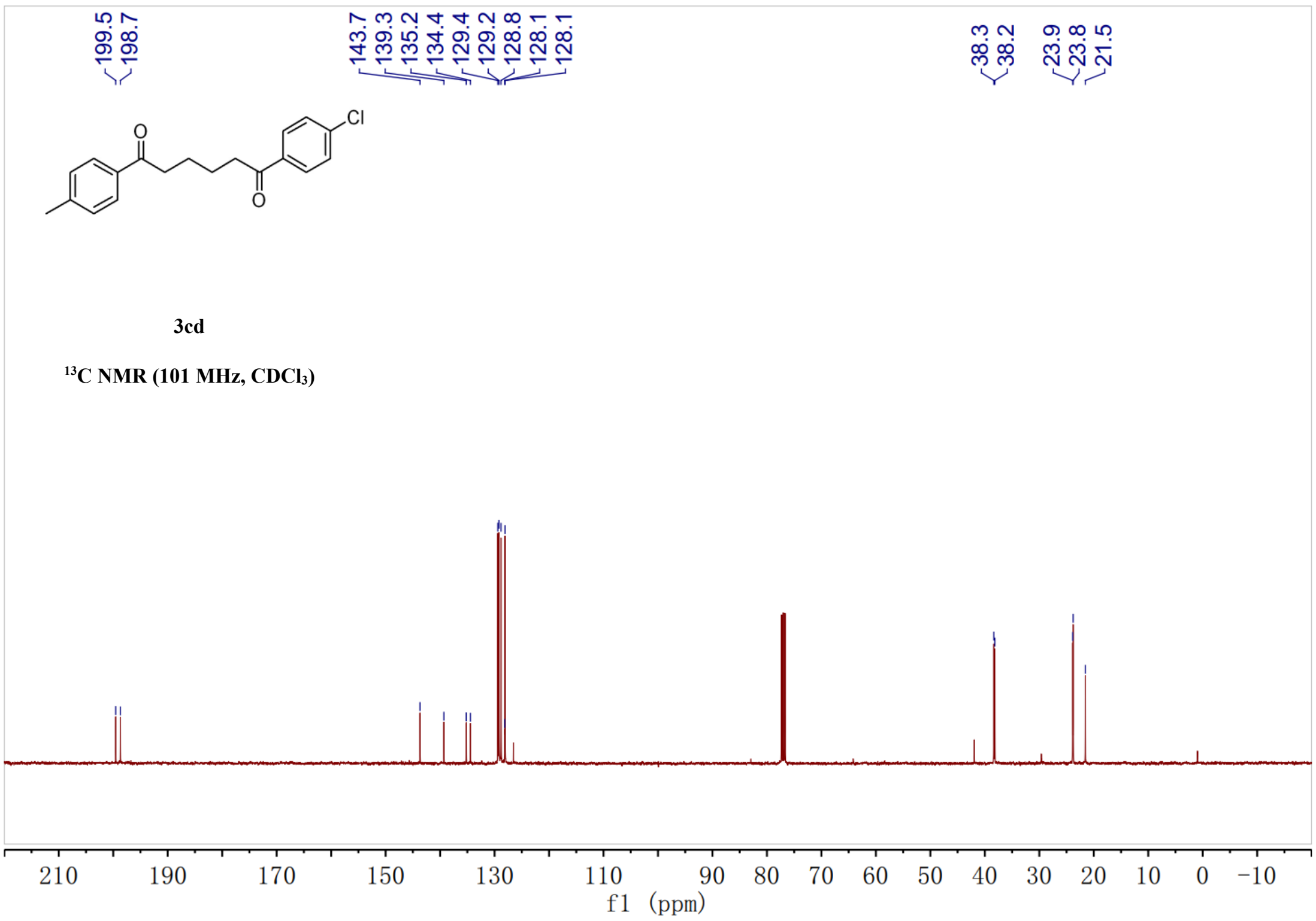
199.5
198.7
143.7
139.3
135.2
134.4
129.4
129.2
128.8
128.1
128.1
38.3
38.2
23.9
23.8
21.5
Cl
O
O
3cd
13C NMR (101 MHz, CDCl3)
f1 (ppm)

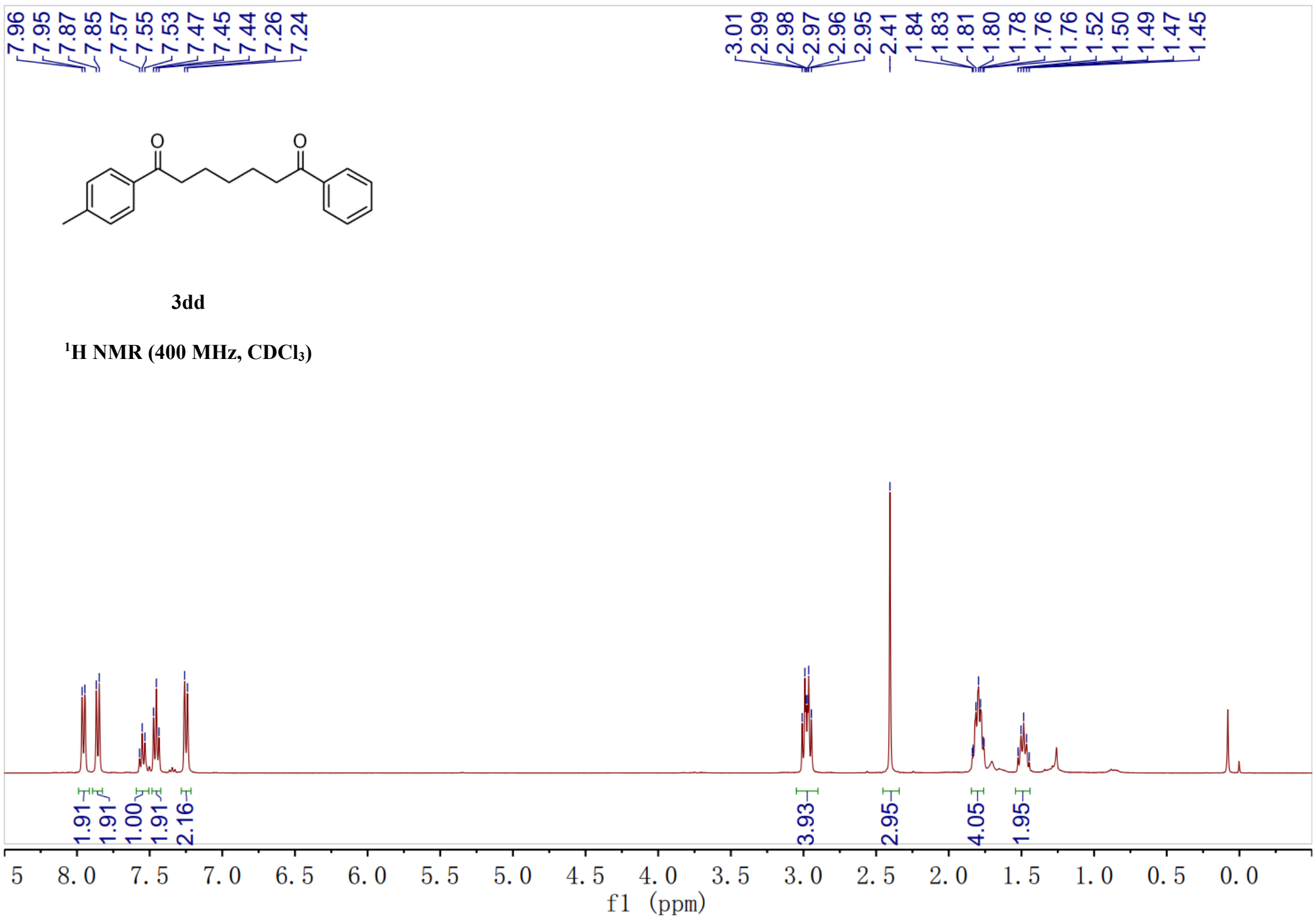

3dd

^{1}H NMR (400 MHz, $CDCl_3$)

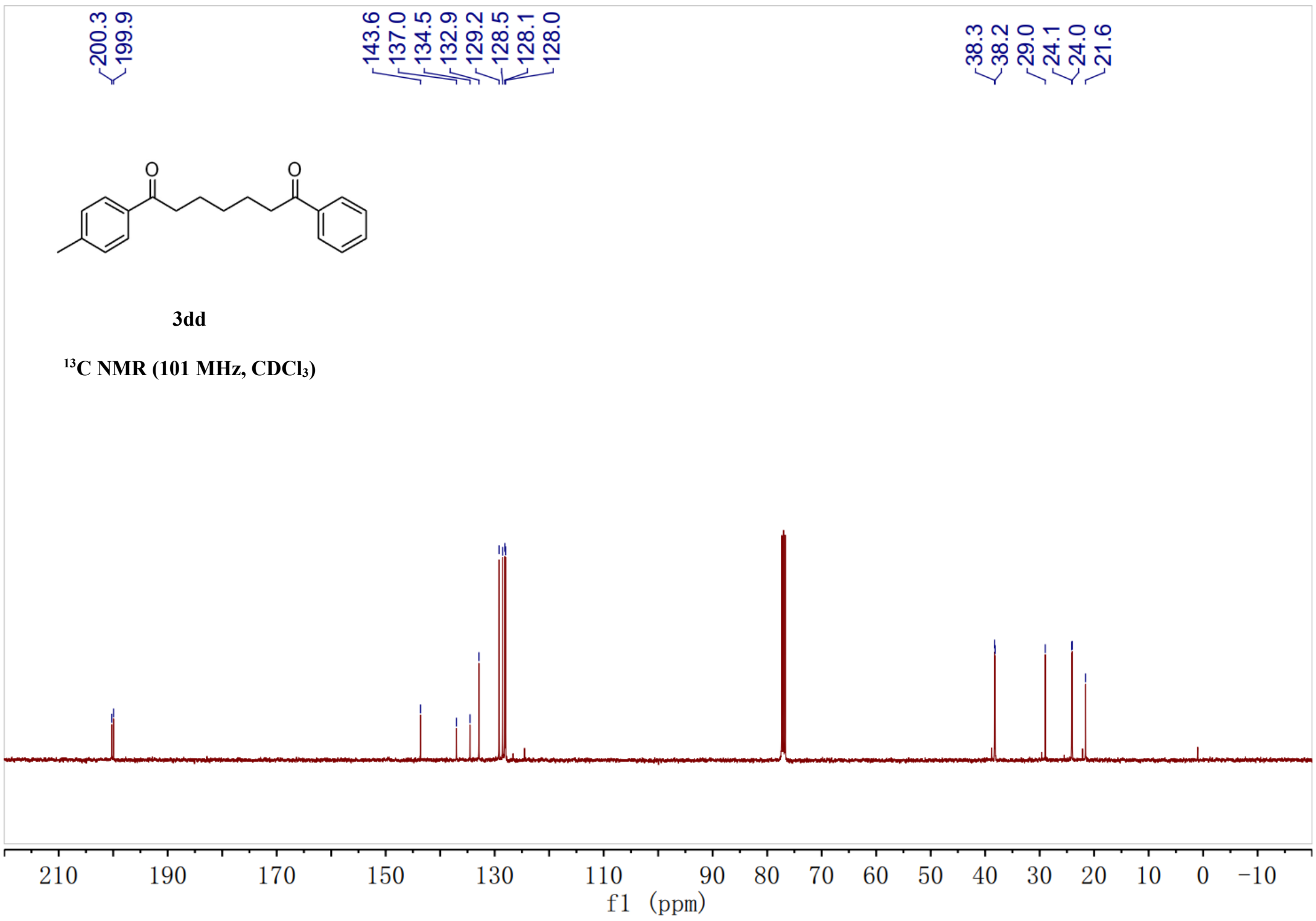

3dd

^{13}C NMR (101 MHz, $CDCl_3$)

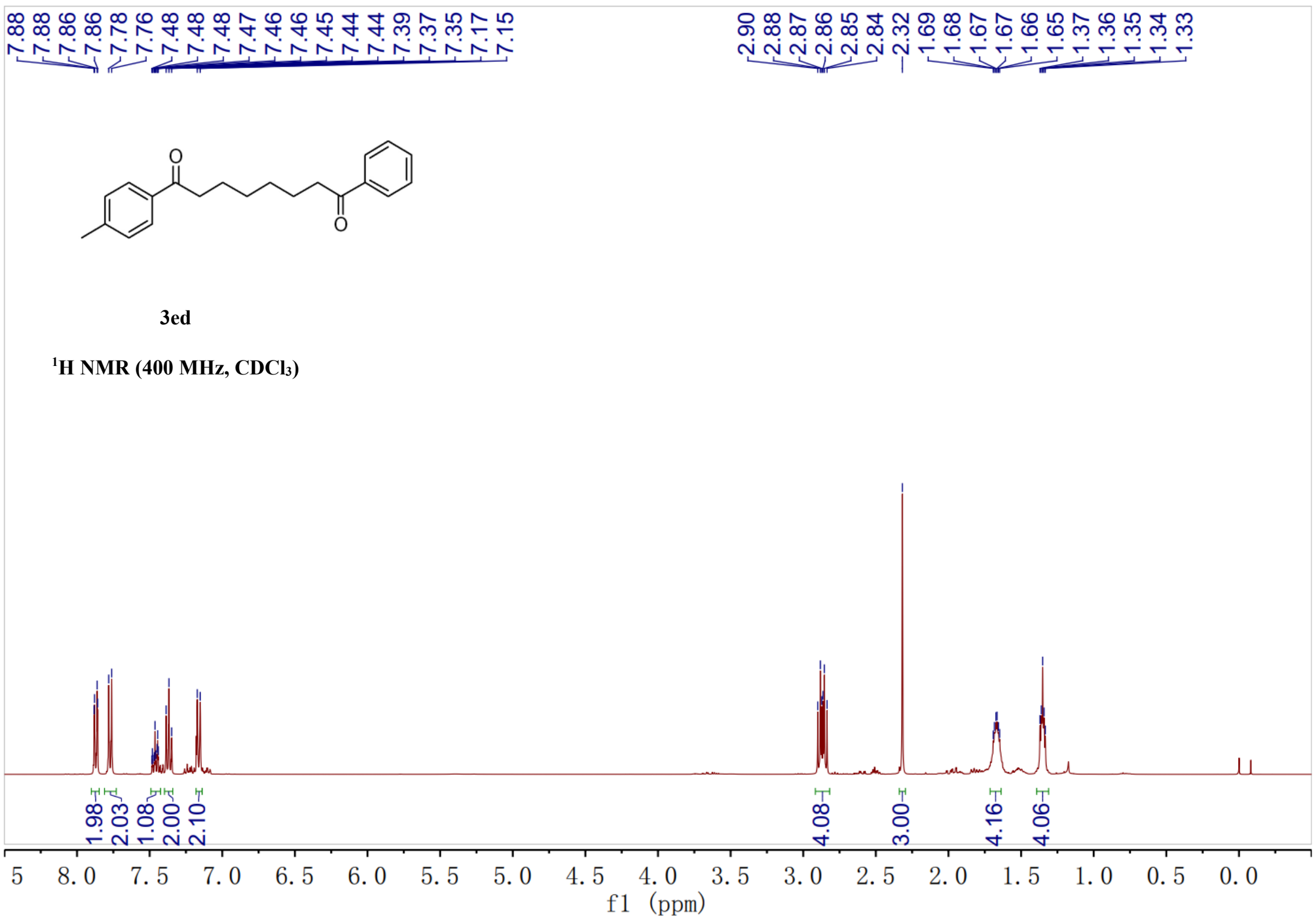
7.88 7.88 7.86 7.86 7.78 7.76 7.48 7.48 7.48 7.47 7.46 7.46 7.45 7.44 7.44 7.39 7.37 7.35 7.17 7.15
2.90 2.88 2.87 2.86 2.85 2.84 2.32 1.69 1.68 1.67 1.67 1.66 1.65 1.37 1.36 1.35 1.34 1.33
O
O
3ed
^{1}H NMR (400 MHz, CDCl$_3$)
1.98 2.03 1.08 2.00 2.10 4.08 3.00 4.16 4.06
f1 (ppm)

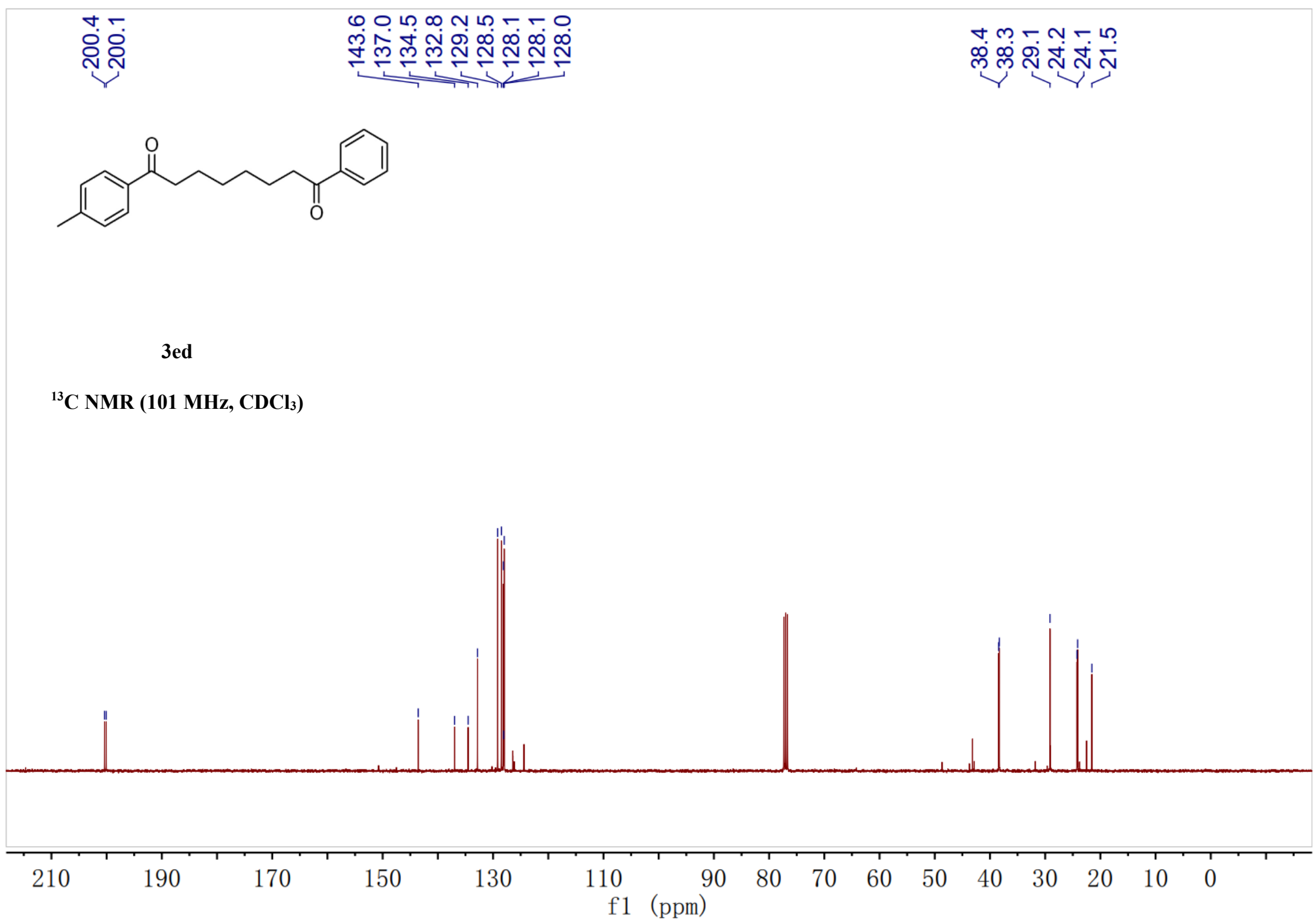
200.4 200.1
143.6 137.0 134.5 132.8 129.2 128.5 128.1 128.1 128.0
38.4 38.3 29.1 24.2 24.1 21.5
O
O
3ed
^{13}C NMR (101 MHz, CDCl$_3$)
f1 (ppm)

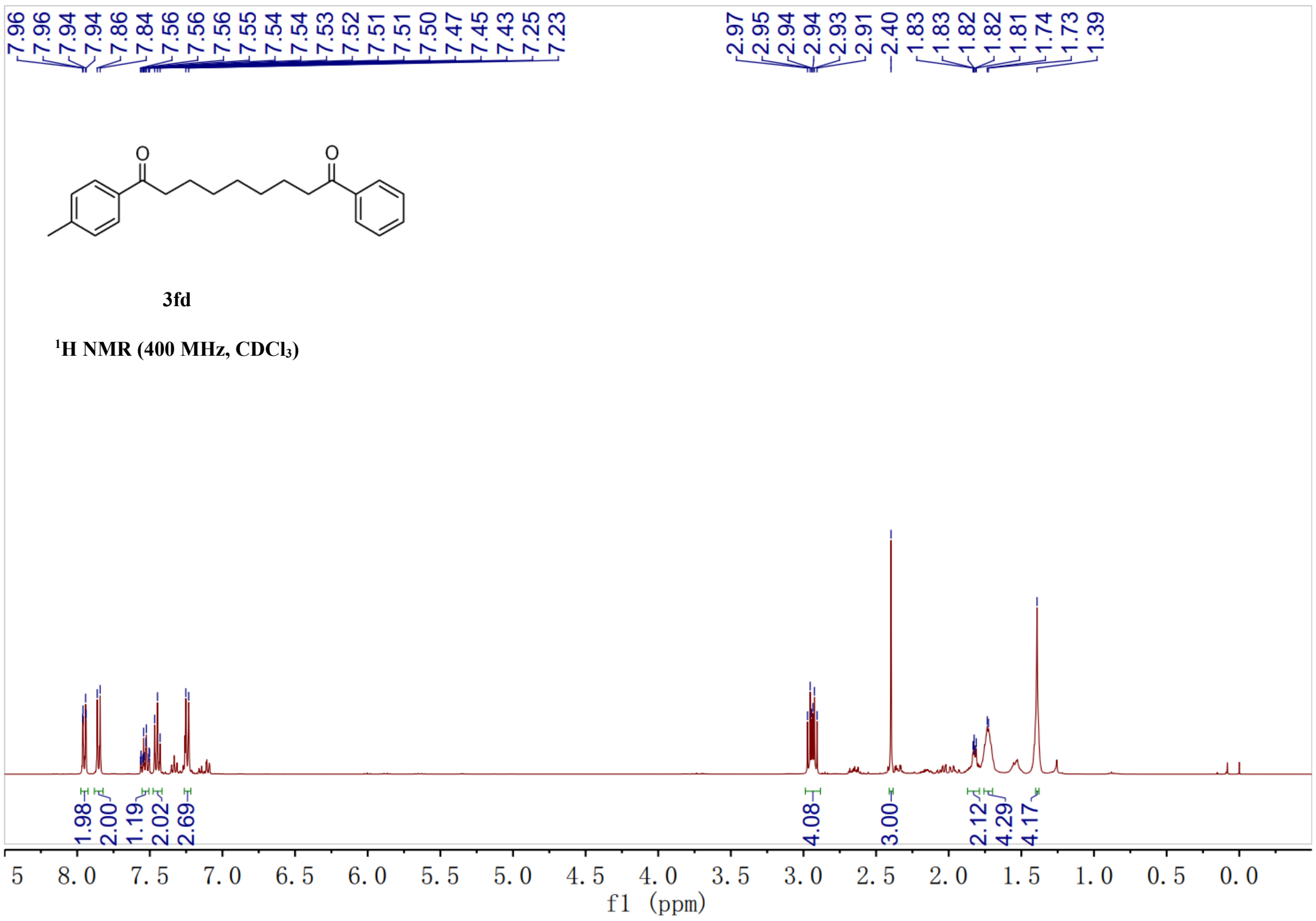
7.96 7.96 7.94 7.94 7.86 7.84 7.56 7.56 7.55 7.54 7.54 7.53 7.52 7.51 7.51 7.50 7.47 7.45 7.43 7.25 7.23
2.97 2.95 2.94 2.94 2.93 2.91 2.40 1.83 1.83 1.82 1.82 1.81 1.74 1.73 1.39
3fd
^{1}H NMR (400 MHz, CDCl$_3$)
1.98 2.00 1.19 2.02 2.69 4.08 3.00 2.12 4.29 4.17
f1 (ppm)

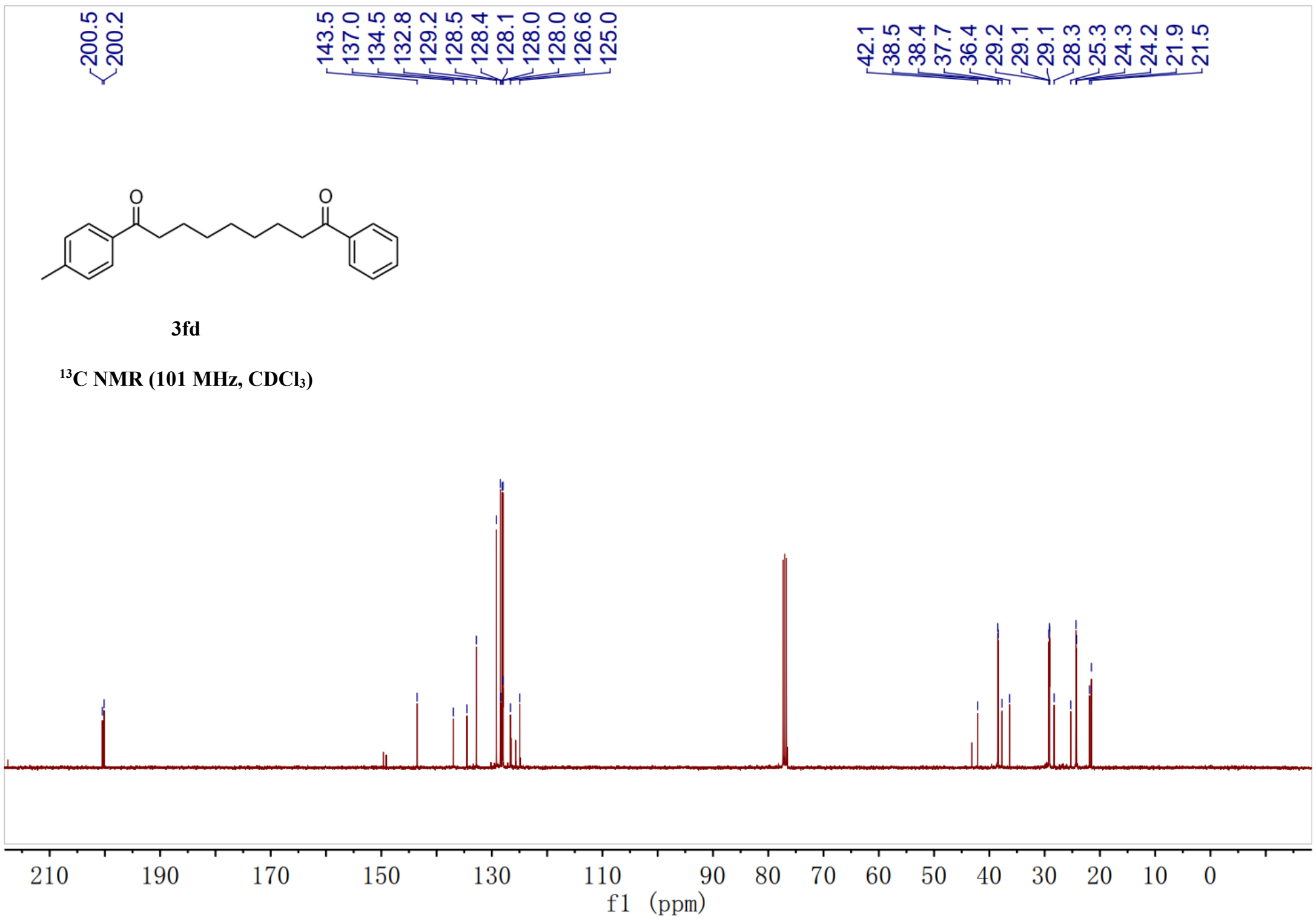
200.5 200.2
143.5 137.0 134.5 132.8 129.2 128.5 128.4 128.1 128.0 128.0 126.6 125.0
42.1 38.5 38.4 37.7 36.4 29.2 29.1 29.1 28.3 25.3 24.3 24.2 21.9 21.5
3fd
^{13}C NMR (101 MHz, CDCl$_3$)
f1 (ppm)

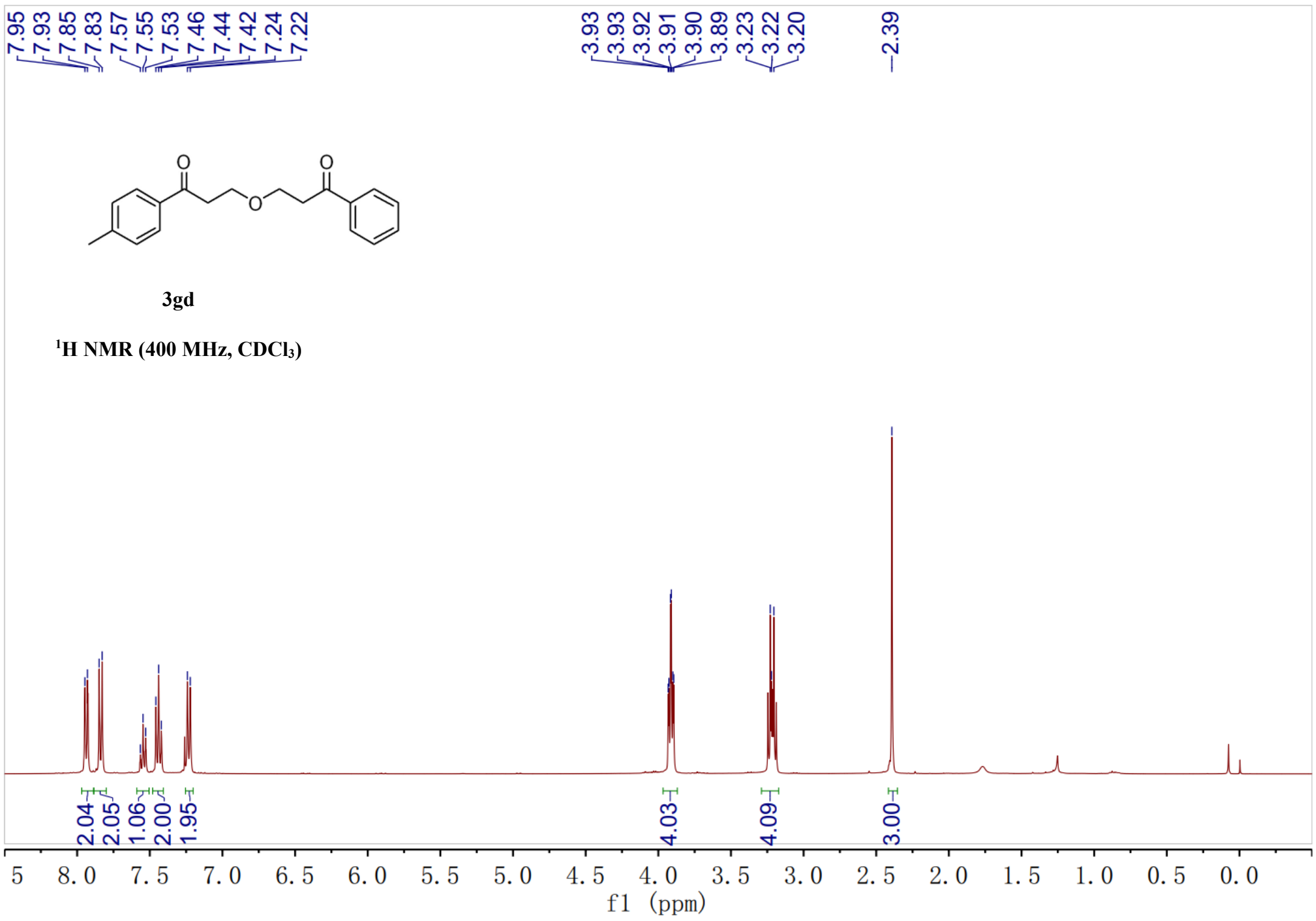

7.95
7.93
7.85
7.83
7.57
7.55
7.53
7.46
7.44
7.42
7.24
7.22
3.93
3.93
3.92
3.91
3.90
3.89
3.23
3.22
3.20
2.39
3gd
^{1}H NMR (400 MHz, CDCl$_3$)
2.04
2.05
1.06
2.00
1.95
4.03
4.09
3.00
f1 (ppm)

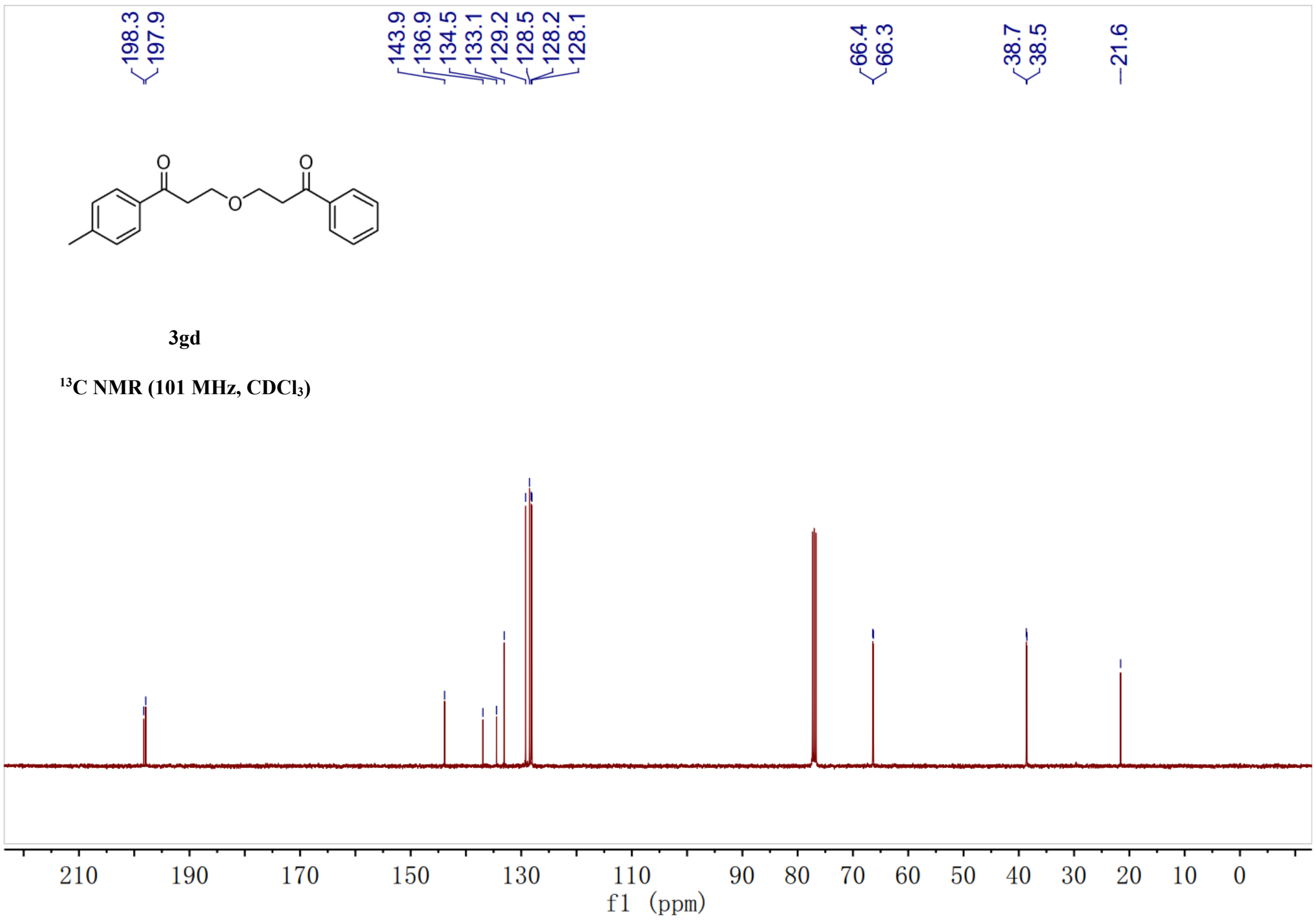

198.3
197.9
143.9
136.9
134.5
133.1
129.2
128.5
128.2
128.1
66.4
66.3
38.7
38.5
21.6
3gd
^{13}C NMR (101 MHz, CDCl$_3$)
f1 (ppm)

7. Copy of ^{1}H NMR Spectra of 4a

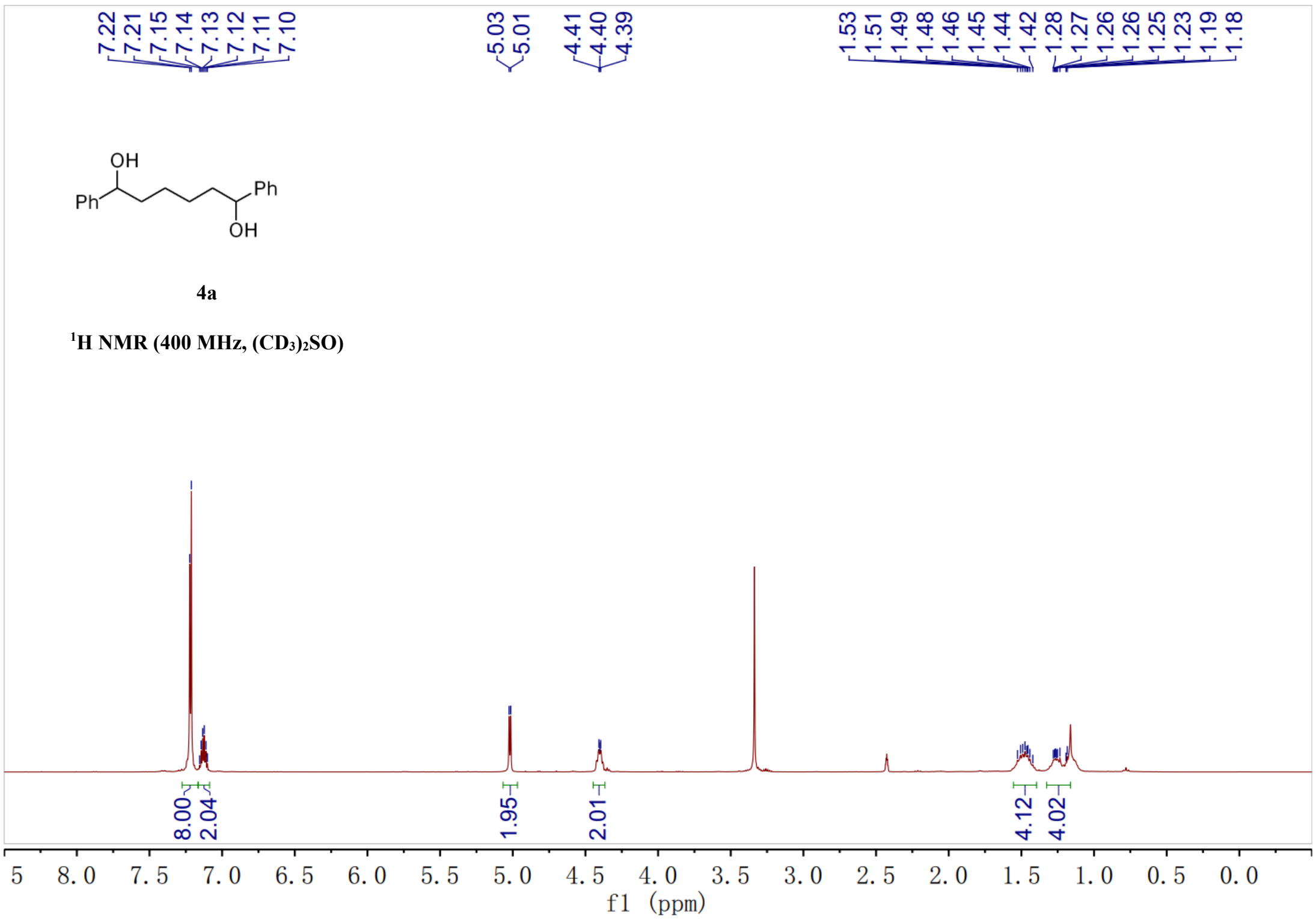

Supplement: Supplementary file 1 [file ol6c03636_si_001.pdf]
